# Aza-Cibalackrot: Turning on Singlet Fission Through Crystal Engineering

**DOI:** 10.1021/jacs.3c00971

**Published:** 2023-05-03

**Authors:** Michael Purdy, Jessica R. Walton, Kealan J. Fallon, Daniel T. W. Toolan, Peter Budden, Weixuan Zeng, Merina K. Corpinot, Dejan-Krešimir Bučar, Lars van Turnhout, Richard Friend, Akshay Rao, Hugo Bronstein

**Affiliations:** † Yusuf Hamied Department of Chemistry, University of Cambridge, Lensfield Rd, Cambridge CB2 1EW, U.K.; ‡ Department of Physics, Cavendish Laboratory, JJ Thomson Avenue, Cambridge CB3 0HE, U.K.; § Department of Chemistry, University College London, 20 Gordon Street, London WC1H 0AJ, U.K.; ∥ Department of Chemistry, University of Sheffield, Dainton Building, Brook Hill, Sheffield S3 7HF, U.K.

## Abstract

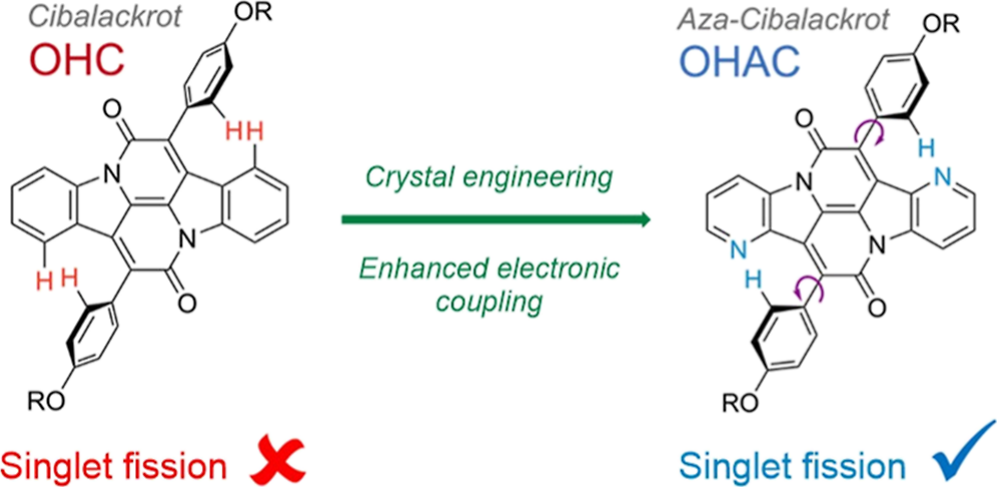

Singlet fission is a photophysical process that provides a pathway for more efficient harvesting of solar energy in photovoltaic devices. The design of singlet fission candidates is non-trivial and requires careful optimization of two key criteria: (1) correct energetic alignment and (2) appropriate intermolecular coupling. Meanwhile, this optimization must not come at the cost of molecular stability or feasibility for device applications. Cibalackrot is a historic and stable organic dye which, although it has been suggested to have ideal energetics, does not undergo singlet fission due to large interchromophore distances, as suggested by single crystal analysis. Thus, while the energetic alignment is satisfactory, the molecule does not have the desired intermolecular coupling. Herein, we improve this characteristic through molecular engineering with the first synthesis of an aza-cibalackrot and show, using ultrafast transient spectroscopy, that singlet fission is successfully “turned on.”

## Introduction

Singlet fission (SF) is a phenomenon that is of particular interest for applications in photovoltaics, as it offers the opportunity to circumvent the Shockley–Queisser limit (29% efficiency^1^) of silicon solar cells by mediating thermal losses through optical means, with a proposed increased efficiency of ∼6%.^2−4^ A material that exhibits singlet fission can be coupled with a silicon solar cell such that energy from the triplet states (if above the band-gap, 1.1 eV) can be harvested by silicon for extraction as electrical energy. This can be achieved through direct transfer of free charges into the band gap or through indirect optical means when coupled with an emitter, such as a quantum dot, a scheme proposed as singlet fission photon-multiplier photovoltaics (SF-PMPVs).^2,4^ This SF-PMPV technology could be readily applied to both old and new silicon solar devices^4^ and may also improve the device lifetime.^5^

SF is an exciton multiplication process, whereby two triplet excitons (T_1_) are formed from a singlet exciton (S_1_) in a spin conserved manner. The process requires an initial electronic interaction between two chromophores in a S_1_ state and ground (S_0_) state, where subsequent energy transfer creates a spin correlated intermediate ^1^(TT) state, which ultimately separates into two independent triplet excitons via a weakly coupled intermediate ^1^(T...T)^6−10^
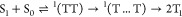
1.1

The energy of the T_1_ state must be approximately half of the S_1_ state, and the material requires a morphology that enables sufficient intermolecular electronic coupling. The solid-state packing of singlet fission materials can be determined via X-ray crystallography of molecular single crystals.^11,12^ Short interchromophore distances and a slip-stacked orientation (along with others) have been shown to facilitate sufficient intermolecular coupling for rapid SF.^12−16^ Despite the importance of the chromophore crystal packing to SF, the optimization of solid-state interactions has rarely been controlled a priori through crystal engineering techniques, which could allow for tuning of intermolecular electronic coupling and enable singlet fission in systems where it otherwise would not occur.^13−16^

The progress of singlet fission technology has been hindered by the limited number of available singlet-fission-active chromophores due to strict energetic and morphological requirements. This is compounded by their molecular instability, which reduces feasibility for device applications. Known molecular systems that adhere to the stipulated energetic and morphological demands include polyacenes,^17−36^ rylene dyes,^11,37−40^ donor–acceptor polymers,^41−47^ and carotenoids,^48,49^ many of which have exhibited ultrafast rates of singlet fission and quantum yields above 100% for the generation of triplets.

Recent work by our group^50,51^ showed that indolonaphthyridine thiophenes (INDTs), an annulated indigo derivative with thiophene groups flanking an aromatic core, have singlet fission propensity when substituted with electron withdrawing groups (such as Br or CN). They also boast superior photo- and ambient stability compared to other popular SF systems. The triplet excitons produced in INDT variants are below the 1.1 eV benchmark required for utilization with silicon photovoltaics. Replacing the thiophene groups of INDT with phenyl groups hypsochromically shifts the triplet state to above 1.1 eV, yielding a molecule known as cibalackrot.^52^ Cibalackrots are highly attractive singlet fission candidates for SF-PMPVs, due to ideal T_1_ energy, a T_1_/S_1_ ratio of ∼0.5, high extinction coefficient, and robust molecular stability.^53,54^ However, a recent study by Ryerson et al.^54^ confirmed that cibalackrot does not undergo efficient singlet fission, as it is hypothesized that the packing arrangement of cibalackrot encouraged other photophysical processes, such as rapid evolution of charge transfer states and excimers, that outcompete singlet fission.^54^

**Figure 1:**
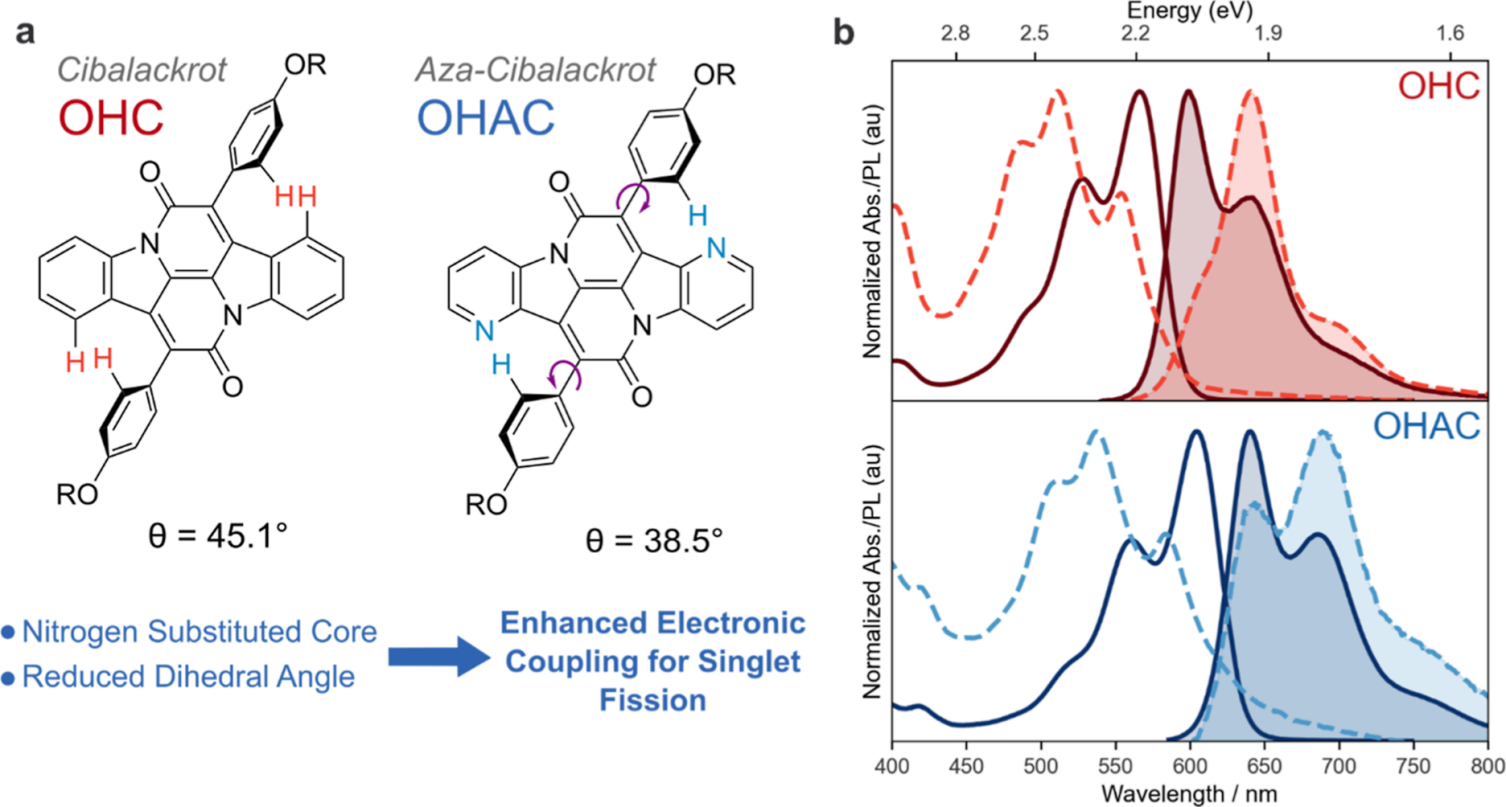
(a) Molecular structures of cibalackrot (R = octyl-hexyl, OHC, shown in red) and novel aza-cibalackrot (OHAC, blue). Inclusion of nitrogen atoms in the aromatic core reduces torsion of the peripheral phenoxy rings while maintaining a favorable T_1_:S_1_ ratio. Increased planarity of the phenoxy rings with respect to the aromatic core from θ = 45.1° to θ = 38.5° (DFT-calculated ground-state-optimized structure at the M062X/G** level) in OHAC is postulated to improve the intermolecular orbital overlap efficiency required for singlet fission. (b) Absorbance (line) and photoluminescence (shaded) spectra of OHC (red) and OHAC (blue) in chlorobenzene solution (darker, solid line) and thin-films (dashed line) prepared by drop casting.

**Figure 2:**
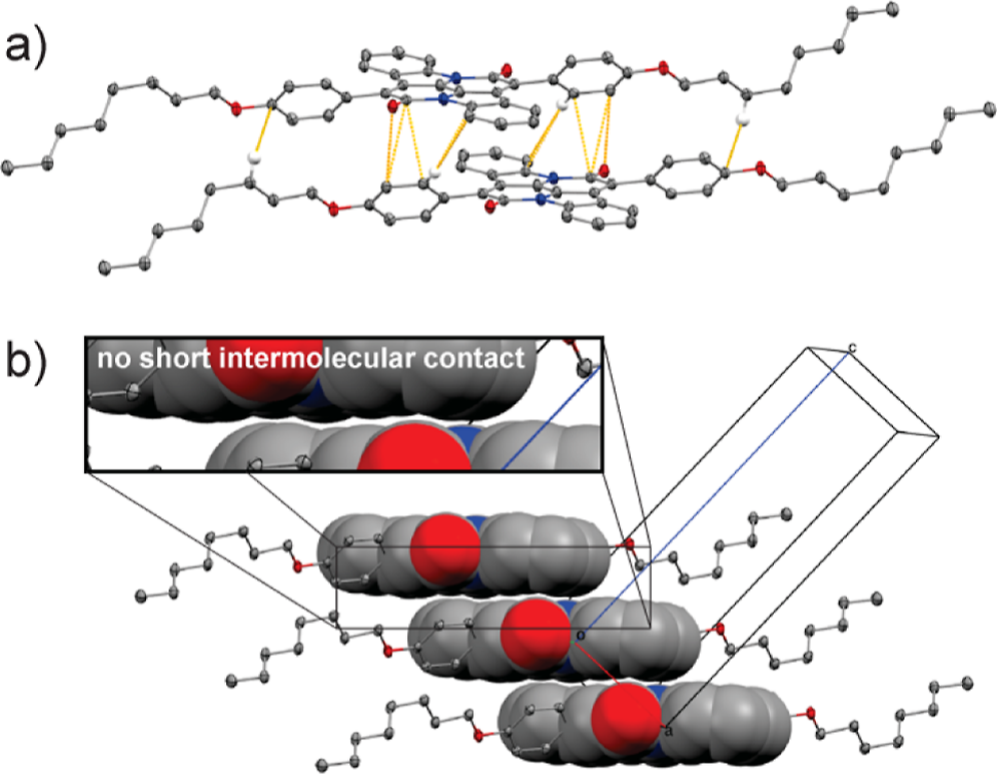
Perspective views of stacked cibalackrot molecules in the crystal structure of OC highlighting: (a) short contacts between the twisted outer phenoxy groups and chromophore cores of neighboring cibalackrot molecules and (b) the lack of short intermolecular contacts between the cibalackrot chromophore within the OC stacks. The thermal ellipsoids are drawn at the 50% probability level, while selected hydrogen atoms are omitted for clarity. Color scheme: carbon–grey, nitrogen–blue, oxygen–red.

A crystallographic analysis of a cibalackrot derivative (Figure 2) revealed that there is a torsion of the peripheral phenyl rings out of the molecular plane (Figure 1a) due to a steric clash between benzylic protons on the molecular core and outer phenyl groups. This results in an increase of the interchromophore distances, inhibiting short-contact interactions by blocking close approach of the aromatic cores. We postulate that this torsion of the phenyl rings is the inhibitive factor for singlet fission in cibalackrot systems. We present a simple chemical alteration to reduce the torsional angle of the phenyl rings by substituting two carbons of the aromatic core with nitrogen atoms (Figure 1a, where the indole moiety is replaced with 4-azaindole), introducing a structurally modified aza-cibalackrot. We, in turn, expect this to reduce the interchromophore distance and increase the intermolecular orbital overlap between the aromatic cores required for singlet fission to be realized in cibalackrot systems. The energetic alignment, solid-state interactions, and photophysics of aza-cibalackrot are directly compared with cibalackrot, and we present evidence of increased singlet fission activity in aza-cibalackrot, observed through ultrafast transient absorption (TA) spectroscopy. We thus demonstrate the effectiveness of our novel crystal engineering strategy and show that singlet fission can be successfully “turned on” by simple modification of the molecular geometry.

### Crystal Structure and Synthetic Design

Octyl-cibalackrot (OC) was subjected to single crystal X-ray diffraction studies to gain insight into its photophysical solid-state properties. The structural analysis has shown that OC crystallizes in the *P* 1̅ space group with one-half of OC in the asymmetric unit. The planar chromophore core of the centrosymmetric cibalackrot closes a torsion angle of 41.6° with its flanking phenoxy groups (Figure 2a). The *n*-octyl side chain of the phenoxy moiety exhibits a non-linear conformation and features one kink in the chain (Figure 2a). The cibalackrot molecules stack in an offset manner along the crystallographic *a* axis (Figure 2b). Within these stacks, the cibalackrot molecules interact through short contacts between the twisted outer phenoxy groups and chromophore cores of neighboring cibalackrot molecules, while no π···π interactions were observed between the chromophore cores (Figure 2b, inset). The crystal structure suggests that the high torsion of the phenoxy groups could be caused by protic clash between the phenoxy group and the main aromatic core, thereby preventing the cibalackrot cores from stacking at a distance that would allow sufficient overlap of the central π systems required for singlet fission.

A number of crystal engineering strategies have been proposed to promote the stacking of conjugated molecules in the crystalline state through π···π interactions.^55^ These include the formation of multi-component crystal forms (such as cocrystals), whereby π-stacking is enhanced through the addition of other conjugated molecules with complementary sizes, shapes, and charge distributions^56,57^ or through the use of hydrogen-bonding molecules that enforce face-to-face stacking of aromatics.^58^ Other popular crystal engineering techniques for the promotion of solid-state π-stacking of chromophores rely on their synthetic modifications.^55^ The most popular approaches are: (1) the addition of bulky auxiliary groups (to prevent edge-to-face stacking),^59^ (2) the halogenation of the chromophore (to steer the formation of structures with desired antiparallel cofacial stacking motifs),^60^ and (3) the installation of heteroatoms into the chromophore structure (to fine-tune Coulomb-attractive interactions that facilitate π-stacking).^61^

We argue that a synthetic modification of cibalackrot is the most promising route to solids with suitably stacked chromophores. The formation of cocrystals and other alternative crystal forms (e.g., polymorphs and solvates) is unlikely to eliminate the protic clash between the phenoxy groups and the main aromatic core and reduce the torsional twist that prohibits the close contacts between the phenoxy groups and the main aromatic core.

Analysis of the optimized OHC ground state structure using density functional theory (DFT, M062X/G** level) showed that heteroaromatic substitution of the cibalackrot core with nitrogen atoms at the 4,4′-positions enables lowering of the dihedral angle by approximately 7° through a reduction of the previously discussed protic clash. We, therefore, develop the first heteroaromatic substituted cibalackrot core, aza-cibalackrot.

### Synthesis

**Scheme 1:**
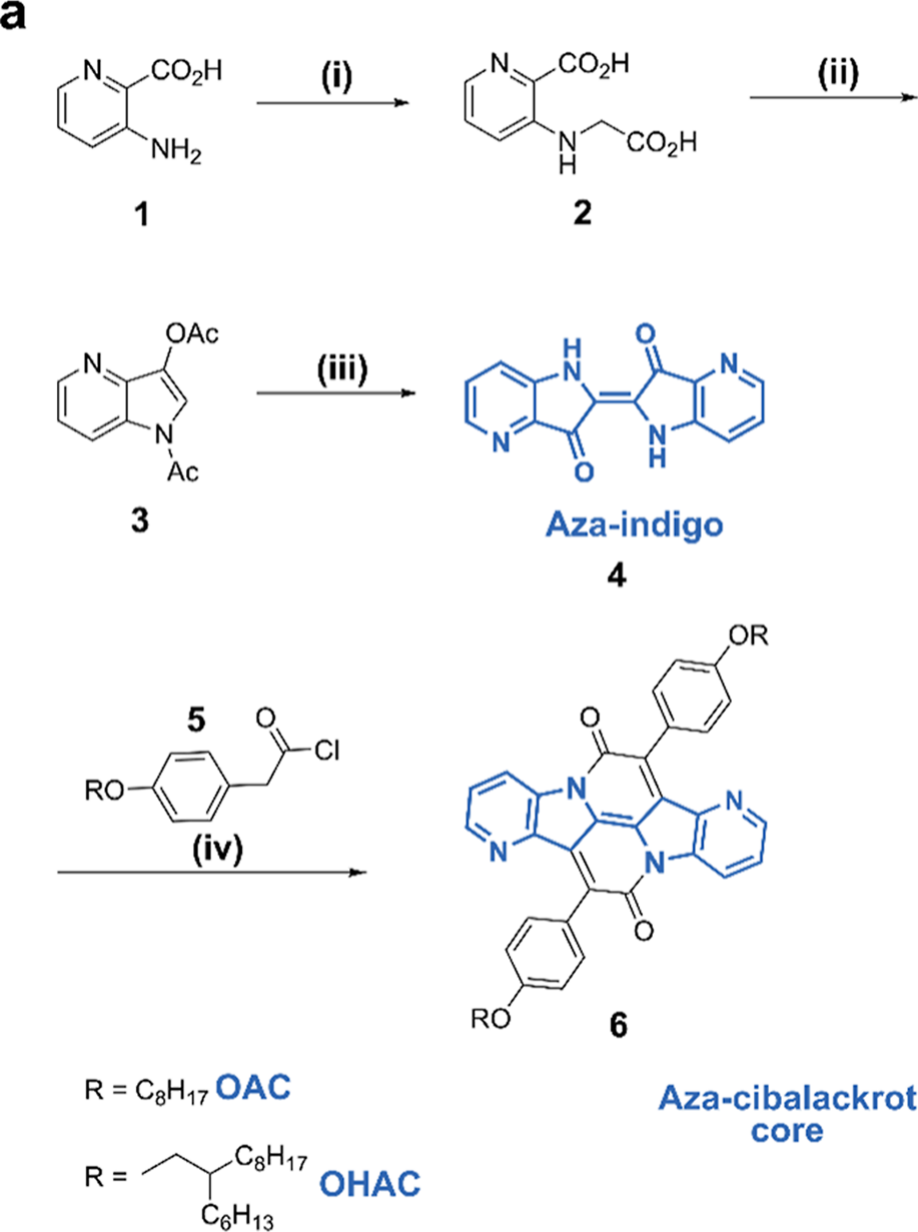
Synthesis of Aza-indigo and OHAC Reagents and conditions: (a) (i) 2-chloroacetic acid (1.1 equiv), K_2_CO_3_ (1.5 equiv), H_2_O, 100° C, 24 h (18%). (ii) fused CH_3_CO_2_K (2.5 equiv), acetic anhydride, 130° C, 1 h (83%). (iii) NH_4_OH (25 mL/g), r.t., 12 h (20%). (iv) 5 (10 equiv), xylenes, 165° C, 12 h (OAC = 0.4%, OHAC = 0.6%)

The procedure presented in Scheme 1 for the synthesis of aza-indigo **4** is adapted from a procedure developed by Sucharda in 1925, and full synthetic procedures and characterization are available in the Supporting Information.^62^ Under basic conditions, 3-picolinic acid is converted to **2** via nucleophilic substitution with 2-chloroacetic acid. The dicarboxylic acid consequently undergoes a ring-closing reaction with fused potassium acetate yielding indole **3**. Finally, an oxidative self-coupling reaction of indole **3** using ammonium hydroxide solution in open air yields aza-indigo **4**. The novel aza-cibalackrots are synthesized by reacting aza-indigo **4** with phenoxy acetyl chloride **5** in a condensation annulation reaction. This procedure offers facile synthesis of aza-indigo, a chromophore that could be used for a plethora of applications, on a multigram scale. The reaction yields of the aza-cibalackrot synthesis were low. However, this could be improved through reaction optimization. One strategy could involve N-substitution of aza-indigo with acetyl groups, as this has proven successful in improving the yields of indigo bay–annulation reactions.^63^

## Results and Discussion

### Characterization

In Figure 1b, the solution-state absorption spectra of OHC and OHAC (solid lines) share similar vibrational progressions, with OHAC redshifted with respect to OHC. To investigate the origin of the redshift, we performed frontier molecular orbital analysis, which showed similar distributions for OHC and OHAC (Supporting information, Figure S26). We observe increased LUMO distribution at the N-substitution positions (4,4′-positions) relative to the HOMO, indicating that our synthetic modifications selectively stabilize the LUMO of OHAC leading to redshifted absorption. Despite the redshift, OHAC still boasts bluer absorbance than our previously reported INDTs.^50^ Excited-state geometry calculations reveal that OHAC has more planar S_1_ and T_1_ states relative to OHC leading to an increase in the excited state conjugation and spin population density of the delocalized diradical on the outer phenoxy groups for OHAC (18%) relative to OHC (12%) (Supporting information, Table S1). This suggests that the increased planarity of the aza-derivative is present in both the ground and excited states, which is the key interaction that governs the rate of singlet fission. Comparison of the absorbance of the solution and thin-film states (Figure 1b, solid vs dashed lines) shows a hypsochromic shift of λ_max_, indicative of preferential slipped stack H-aggregate formation in thin films.^64,65^ The π–π stacking of the aromatic cores provides essential intermolecular orbital coupling required for singlet fission. We were unable to grow aza-cibalackrot single crystals (see the Supporting information for details of crystallization experiments); therefore, grazing-incidence wide-angle X-ray scattering (GIWAXS) was employed to gain further insight into the microstructure of both cibalackrot and aza-cibalackrot. GIWAXS was performed on OC and OAC (octyl aza-cibalackrot) thin films drop-cast from chlorobenzene. The GIWAXS data (presented in the Supporting information, Figure S3) indicates that both OC and OAC films are highly crystalline with several large, randomly orientated crystal grains. For the OC film, the crystal structure observed via GIWAXS is consistent with the single-crystal X-ray diffraction studies. The modifications of OAC have a considerable impact on crystal packing. We speculate that reducing the torsion of the peripheral phenoxy rings alters how the octyl groups are arranged in the unit cell, with octyl groups being interdigitated for OC and non-interdigitated for OAC. GIWAXS analysis was performed on drop-cast thin films of cibalackrot (OHC) and aza-cibalackrot (OHAC) substituted with 2-hexyldecane chains. However, due to the large-branched alkyl chains present on the structures, the thin-films were highly amorphous, and it was difficult to infer anything significant about the microstructure other than both exhibited lamellae packing (Supporting information, Figure S2). Measurements of the photoluminescence quantum efficiency (PLQE) showed highly emissive solutions for OHAC (74%) and OHC (86%) and a large quenching of photoluminescence (PL) in thin film for both, with PLQEs of 0.5 and 4%, respectively. This quenching can be attributed to both sub-radiance due to H-aggregation^64^ and new non-radiative pathways becoming active in the solid state, such as singlet fission. The PL spectra obtained by PLQE measurements show a suppressed 0–0 emission peak in thin film, which is attributed to both H-aggregation effects and self-reabsorption. Time-resolved PL measurements of OHAC indicated a rapid PL decay of 7.5 ± 0.5 ns in solution, which is reduced to 2.53 ± 0.03 ns in thin film (Supporting information, Figure S4). Due to the very low PLQE of the OHAC thin film (0.5%), it is likely that most photogenerated singlet excitons have a different, non-radiative fate.

### Evidence for Singlet Fission

**Figure 3:**
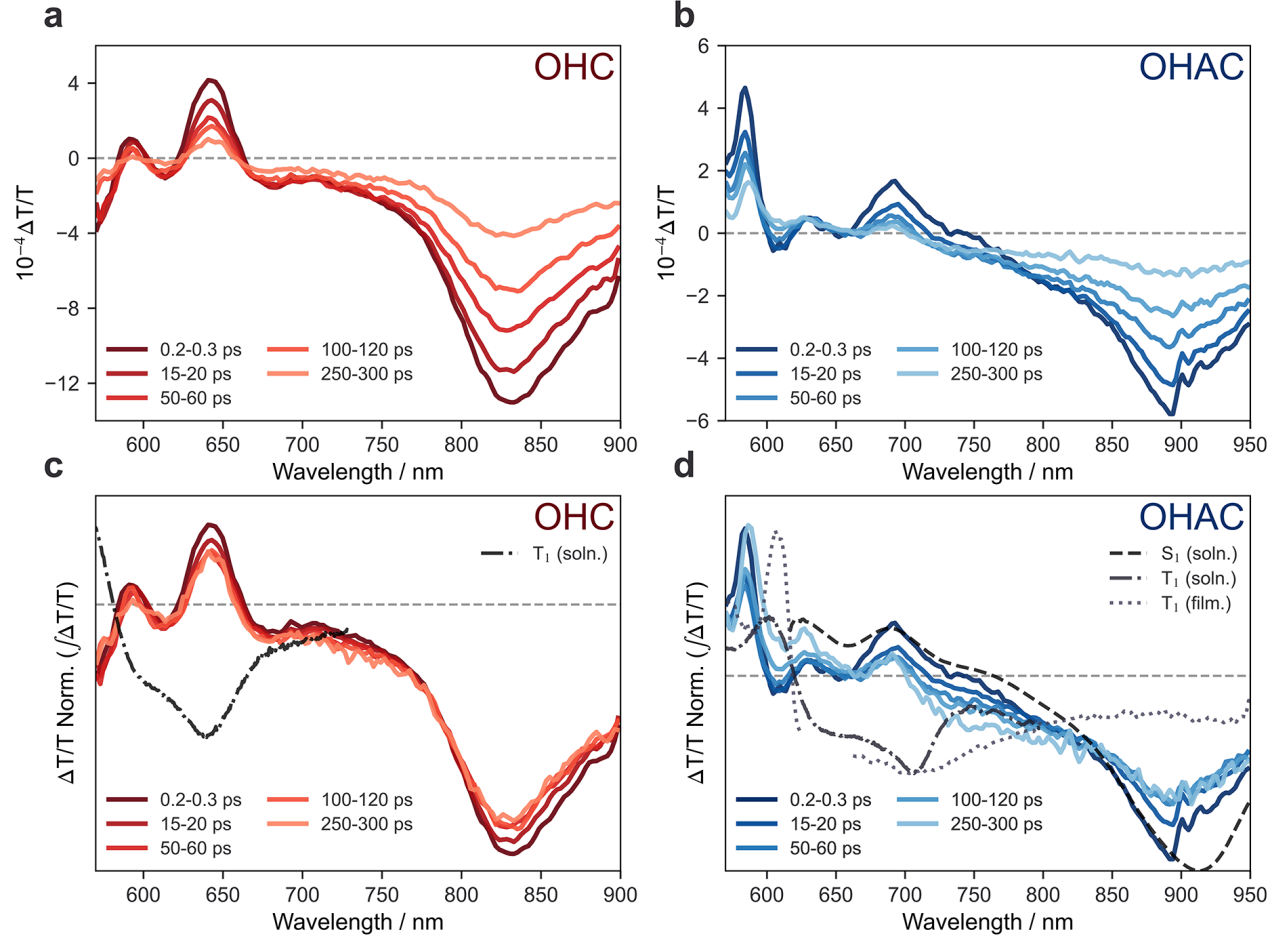
Ultrafast dynamics of OHC (red, a) and OHAC (blue, b), performed in thin films (TA spectroscopy, 550 nm excitation). Spectra normalized to integral Δ*T*/*T* shown in (**c**,d). The OHC signal is indicative of single species, whereas OHAC indicates the presence of two species. Results concordant with those seen by Fallon et al.,^50^ attributed to the formation of a geminate TT state, indicative of singlet fission. Triplet sensitization spectra (dot-dashed/dotted) attributed to free triplets in solution (anthracene sensitizer in chlorobenzene, 355 nm excitation) and free triplets in thin film (*meso*-tetraphenyl-tetrabenzoporphine palladium complex sensitizer, 650 nm excitation) are added for reference (Supporting information Figures S6–S13).

We investigate the singlet fission capability of OHAC in comparison with OHC, known to be singlet fission inactive,^54^ using transient absorption spectroscopy. Figure 3 shows the Δ*T*/*T* spectra for OHC (Figure 3a) and OHAC (Figure 3b) measured in thin films under 550 nm photoexcitation. Negative signals correspond to photoinduced absorption (PIA) of the excited states, and positive signals correspond to ground state bleach (GSB) or stimulated emission. The spectra of both materials are dominated by a strong PIA from the singlet states, with a maximum at 840 and 890 nm for OHC and OHAC, respectively. In Figure 3c,d, we normalize the TA spectra shown in Figure 3a,b, respectively, such that the integrated area under the curves is equivalent. This allows for the change in spectral shapes as a function of time to be better visualized. As can be seen in Figure 3c, the spectra of OHC showed little spectral evolution in the first 300 ps, indicating that no new species are formed from the singlet state. This is consistent with previous reports that singlet fission does not occur in these systems.

In contrast, the normalized spectra of OHAC (Figure 3d) show pronounced spectral shifts in the first 300 ps. A new PIA is seen to grow in, centered at 740 nm, concomitant with a decay of the PIA at 890 nm. For comparison, we overlay the singlet spectra measured in dilute solutions (black dashed line), the triplet spectra measured via sensitization in solution (black dot-dashed line), and the triplet spectra measured in thin film blends with a triplet sensitizer (black dotted line). The full solution and sensitization spectra can be found in the Supporting information (Figures S6–S13).

Comparison with these spectra show that the early time OHAC spectrum, which we label Species 1, is well matched with the singlet spectrum from dilute solutions, with a PIA centered at 900 nm and a stimulated emission band at 700 nm. Hence, we assign Species 1 to the singlet exciton, S_1_, in OHAC. The S_1_ state is then quenched, and a new species, which we label Species 2, grows in with a broad PIA in the region relevant to the triplet species, as seen from the comparison between TA spectra of free triplets in OHAC solution and film blend. Thus, we conjecture that Species 2 must be connected either with free triplets or the intermediate^1^(TT). We note that the spectra of free triplets in solution and film sensitization closely resemble each other in both GSB and PIA, while the pristine OHAC film exhibits both a blueshifted GSB and a redshifted triplet region. This suggests that the introduction of the triplet sensitizer may disrupt the crystalline packing of OHAC and yield free triplets in amorphous environments in the film (more closely resembling free triplets in solution). The film sensitization spectrum is thus not completely representative of free triplets in a highly crystalline OHAC region but nonetheless gives an indication of the spectral shape to be expected. Thus, the apparent redshift of the Species 2 ^1^(TT) PIA in OHAC film is consistent with greater aggregation in the pristine film, resulting in broadening, as observed in other organic systems.^50,66^ Similar PIAs and apparent redshifting of the triplet regions were observed in our previous measurements of INDT systems, where triplets formed rapidly via singlet fission (summarized in Supporting information Table S5).

**Figure 4:**
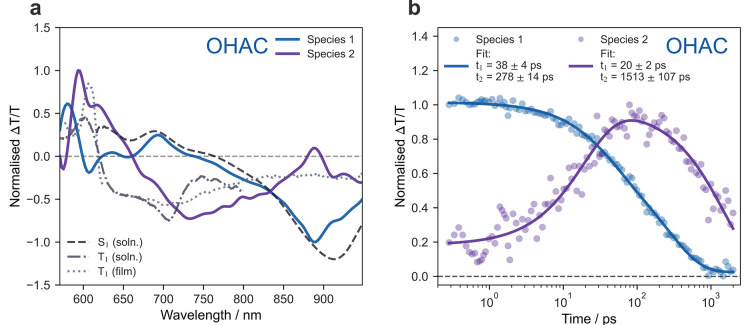
(a) Two-species spectral decomposition of OHAC ps-TA data (from Figure 3b), using a genetic algorithm (see Supporting information Figure S17). Spectra are compared to the singlet S_1_ spectrum in solution and spectra of free triplets, T_1_, in solution and thin film from sensitization experiments (Supporting information Figures S6–S13). We assign Species 1 as the S_1_ state and Species 2 as the ^1^TT state. (b) Normalized kinetics of species. Species 1 decays with concomitant growth of Species 2. Decay of species 2 is independent of excitation density (Supporting information Figure S5), indicating geminate TT recombination.

To gain further insights into the timescale for the growth of Species 2 in OHAC, a spectral decomposition was performed with a genetic algorithm (GA). Figure 4a shows the spectra of the two species retrieved from the GA. Figure 4b shows the normalized deconvoluted kinetics associated with Species 1 and 2 (data points), fitted to exponential decays (solid line, rates shown in Figure 4b). Species 1 (S_1_) rapidly decays with a lifetime of 38 ± 4 ps, with approximately concomitant growth of Species 2 (^1^TT, 21 ± 2 ps). Species 1 transforms into Species 2 with an approximate yield of 29–32% (Supporting information, Table S8), a value that is concurrent with additional methods to determine the yield (29–60%, Supporting information, Table S9). The GA results were compared to other relevant decomposition methods (see Supporting information Figures S16–S22). Alternative decomposition methods may indicate that the formation of Species 2 is reversible, with an equilibrium between the states (Supporting information, Figure S22). The remaining Species 1 population undergoes non-radiative decay, with a lifetime of 278 ± 14 ps. Species 2 decays with a longer lifetime of 1513 ± 107 ps. This decay was also found to be fluence independent (Supporting information, Figure S5). The short lifetime and fluence independence indicate that Species 2 is a geminate TT state. The rapid decay of Species 2 yields a lifetime too short to confirm its identity with electron spin resonance. However, we rule out that Species 2 is formed from intersystem crossing due to the clear absence of a triplet signature in the TA of OHAC solution (Supporting information, Figure S6 and S7).

The rapid decay of Species 2 suggests that the geminate TT state cannot dissociate to free triplets with the current film preparation. It is possible that the increase in electronic coupling between the chromophores reduces the ability for the triplets to dissociate. Overall, the presence of TT states indicates greater propensity for singlet fission in OHAC than the OHC. Time-dependent DFT calculations suggest that OHAC and OHC have a T_1_/S_1_ ratio of 0.50 and 0.53, respectively; therefore, singlet fission is predicted to be isothermic in both cases. Therefore, the increased ability for OHAC to undergo singlet fission is a result of heightened electronic coupling, as opposed to improved energy-level alignment. Electronic coupling to TT states can often be charge-transfer mediated in singlet fission, and a previous study by our group showed that INDT derivatives have charge-transfer contribution to the photoexcited S_1_ state.^67^ Therefore, while we did not observe direct evidence of charge transfer states in the TA data, it is possible the singlet fission mechanism may be charge-transfer mediated in OHAC.

## Conclusions

In this work, we have shown that the search for singlet fission chromophores suitable for photovoltaic applications can be adapted by considering crystal structure engineering, beyond the scope of side-chain engineering, to enhance intermolecular couplings. These strategies can be applied to materials previously identified as SF candidates that did not fulfill the potential of their favorable energetic alignments. We introduce a novel molecule, aza-cibalackrot, through these crystal engineering strategies, that has been examined in comparison to its parent species, cibalackrot. We applied ultrafast spectroscopy to confirm that the singlet fission behavior is enhanced due to our change in molecular design, shown by the formation of a geminate TT pair in aza-cibalackrot. DFT confirmed that our energy level alignment has not been significantly altered, indicating that the change in singlet fission activity is a direct result of increased intermolecular coupling. We believe that the same strategies and considerations can be applied for other organic chromophore systems in the future, where singlet fission seems energetically viable, and yet the propensity for singlet fission is elusive.

## Methods

Thin films were generated by drop-casting from chlorobenzene in an inert atmosphere, encapsulated with glass sealed by epoxy resin. Steady-state UV–vis measurements of films and solutions (chlorobenzene, 0.1 mM concentration) were performed with a Shimadzu UV3600Plus spectrometer. Steady-state PL measurements were performed using a home built integrating sphere PLQE apparatus, using methods described by de Mello et al.,^68^ with 520 nm excitation at 1 mW power. Transient PL measurements were performed using time-correlated single photon counting (TCSPC) with 510 nm excitation with Edinburgh Instruments FLS1000-DD-stm. Ultrafast TA measurements were performed using previously reported methods^50^ discussed in the Supporting Information. A short excitation pulse creates a change in the population of states, the transitions between which are sampled with a probe pulse at multiple time delays, achieved with a highly controllable mechanical delay stage. Samples were interrogated with 40 kHz 550 nm excitation with 646 μm spot size at 825 μW (12.6 μJ cm^–2^). Triplet sensitization measurements were performed in solution with anthracene^69,70^ triplet sensitizer with 355 nm excitation and a *meso*-tetraphenyl-tetrabenzoporphine palladium complex sensitizer in solution and thin film, with 650 nm excitation. Spectral deconvolution with a GA was performed with methods reported previously.^71,72^

### Computational Methods

Quantum calculations were performed on each structure to investigate the effect that our structural modifications had on the geometry, excited-state energies, and spin population density of OHC and OHAC. All S_0_- and T_1_-optimized geometries were obtained using the M062X^73^ functional together with the 6-311G** basis set.^74^ Based on the optimized ground state geometries, the vertical excitation energy levels were evaluated at the TD-DFT B3LYP/G** level.^50^ The adiabatic excitation of the T_1_ states were evaluated with a ΔSCF procedure at the DFT M062X/G** level, which manually adjusts the spin multiplicities for both vertical and adiabatic geometries. The electron spin density distributions were analyzed with Multiwfn 3.7^75^ based on the relaxed T_1_ state geometries. The spin population on the outer phenoxy groups (ηPh) was obtained by performing Mulliken population analysis in Multiwfn.

## Supporting Information Available

Synthetic methods, structural characterization, crystal data, spectroscopic characterization, and computational analysis (PDF)

## Abbreviations

SF: singlet fission
OHC: octyl-hexyl-cibalackrot
OHAC: octyl-hexyl-aza-cibalackrot
OC: octyl-cibalackrot
OAC: octyl-aza-cibalackrot

## References

[ref1] Shockley W.; Queisser H. J. Detailed Balance Limit of Efficiency of P-n Junction Solar Cells. J. Appl. Phys. 1961, 32, 510–519. 10.1063/1.1736034.

[ref2] Rao A.; Friend R. H. Harnessing Singlet Exciton Fission to Break the Shockley–Queisser Limit. Nat. Rev. Mater. 2017, 2, 17063 10.1038/natrevmats.2017.63.

[ref3] Allardice J. R.; Thampi A.; Dowland S.; Xiao J.; Gray V.; Zhang Z.; Budden P.; Petty A. J.; Davis N. J. L. K.; Greenham N. C.; Anthony J. E.; Rao A. Engineering Molecular Ligand Shells on Quantum Dots for Quantitative Harvesting of Triplet Excitons Generated by Singlet Fission. J. Am. Chem. Soc. 2019, 141, 12907–12915. 10.1021/jacs.9b06584. 31336046 PMC7007228

[ref4] Futscher M. H.; Rao A.; Ehrler B. The Potential of Singlet Fission Photon Multipliers as an Alternative to Silicon-Based Tandem Solar Cells. ACS Energy Lett. 2018, 3, 2587–2592. 10.1021/acsenergylett.8b01322. 30345370 PMC6189909

[ref5] Jiang Y.; Nielsen M. P.; Baldacchino A. J.; Green M. A.; McCamey D. R.; Tayebjee M. J. Y.; Schmidt T. W.; Ekins-Daukes N. J. Singlet Fission and Tandem Solar Cells Reduce Thermal Degradation and Enhance Lifespan. Prog. Photovoltaics Res. Appl. 2021, 29, 899–906. 10.1002/pip.3405.

[ref6] Musser A. J.; Clark J. Triplet-Pair States in Organic Semiconductors. Annu. Rev. Phys. Chem. 2019, 70, 323–351. 10.1146/annurev-physchem-042018-052435. 31174458

[ref7] Wang Z.; Liu H.; Xie X.; Zhang C.; Wang R.; Chen L.; Xu Y.; Ma H.; Fang W.; Yao Y.; Sang H.; Wang X.; Li X.; Xiao M. Free-Triplet Generation with Improved Efficiency in Tetracene Oligomers through Spatially Separated Triplet Pair States. Nat. Chem. 2021, 13, 559–567. 10.1038/s41557-021-00665-7. 33833447

[ref8] Miyata K.; Conrad-Burton F. S.; Geyer F. L.; Zhu X. Y. Triplet Pair States in Singlet Fission. Chem. Rev. 2019, 119, 4261–4292. 10.1021/acs.chemrev.8b00572. 30721032

[ref9] Alagna N.; Han J.; Wollscheid N.; Perez Lustres J. L.; Herz J.; Hahn S.; Koser S.; Paulus F.; Bunz U. H. F.; Dreuw A.; Buckup T.; Motzkus M. Tailoring Ultrafast Singlet Fission by the Chemical Modification of Phenazinothiadiazoles. J. Am. Chem. Soc. 2019, 141, 8834–8845. 10.1021/jacs.9b01079. 31063690

[ref10] Bossanyi D. G.; Matthiesen M.; Wang S.; Smith J. A.; Kilbride R. C.; Shipp J. D.; Chekulaev D.; Holland E.; Anthony J. E.; Zaumseil J.; Musser A. J.; Clark J. Emissive Spin-0 Triplet-Pairs Are a Direct Product of Triplet–Triplet Annihilation in Pentacene Single Crystals and Anthradithiophene Films. Nat. Chem. 2021, 13, 163–171. 10.1038/s41557-020-00593-y. 33288892

[ref11] Le A. K.; Bender J. A.; Arias D. H.; Cotton D. E.; Johnson J. C.; Roberts S. T. Singlet Fission Involves an Interplay between Energetic Driving Force and Electronic Coupling in Perylenediimide Films. J. Am. Chem. Soc. 2018, 140, 814–826. 10.1021/jacs.7b11888. 29240411

[ref12] Smith M. B.; Michl J. Recent Advances in Singlet Fission. Annu. Rev. Phys. Chem. 2013, 64, 361–386. 10.1146/annurev-physchem-040412-110130. 23298243

[ref13] Buchanan E. A.; Kaleta J.; Wen J.; Lapidus S. H.; Císařová I.; Havlas Z.; Johnson J. C.; Michl J. Molecular Packing and Singlet Fission: The Parent and Three Fluorinated 1,3-Diphenylisobenzofurans. J. Phys. Chem. Lett. 2019, 10, 1947–1953. 10.1021/acs.jpclett.8b03875. 30883125

[ref14] Papadopoulos I.; Zirzlmeier J.; Hetzer C.; Bae Y. J.; Krzyaniak M. D.; Wasielewski M. R.; Clark T.; Tykwinski R. R.; Guldi D. M. Varying the Interpentacene Electronic Coupling to Tune Singlet Fission. J. Am. Chem. Soc. 2019, 141, 6191–6203. 10.1021/jacs.8b09510. 30854854

[ref15] Felter K. M.; Grozema F. C. Singlet Fission in Crystalline Organic Materials: Recent Insights and Future Directions. J. Phys. Chem. Lett. 2019, 10, 7208–7214. 10.1021/acs.jpclett.9b00754. 31689105 PMC6875870

[ref16] Yost S. R.; Lee J.; Wilson M. W. B.; Wu T.; McMahon D. P.; Parkhurst R. R.; Thompson N. J.; Congreve D. N.; Rao A.; Johnson K.; Sfeir M. Y.; Bawendi M. G.; Swager T. M.; Friend R. H.; Baldo M. A.; Van Voorhis T. A Transferable Model for Singlet-Fission Kinetics. Nat. Chem. 2014, 6, 492–497. 10.1038/nchem.1945. 24848234

[ref17] Singh S.; Jones W. J.; Siebrand W.; Stoicheff B. P.; Schneider W. G. Laser Generation of Excitons and Fluorescence in Anthracene Crystals. J. Chem. Phys. 1965, 42, 330–342. 10.1063/1.1695695.

[ref18] Klein G.; Voltz R.; Schott M. Magnetic Field Effect on Prompt Fluorescence in Anthracene: Evidence for Singlet Exciton Fission. Chem. Phys. Lett. 1972, 16, 340–344. 10.1016/0009-2614(72)80288-4.

[ref19] Bouchriha H.; Ern V.; Fave J. L.; Guthmann C.; Schott M. Magnetic Field Dependence of Singlet Exciton Fission and Fluorescence in Crystalline Tetracene At 300 K. J. Phys. 1978, 39, 257–271. 10.1051/jphys:01978003903025700.

[ref20] Arnold S.; Swenberg C. E.; Pope M. Triplet Exciton Caging in Two Dimensions: Magnetic Field Effects. J. Chem. Phys. 1976, 64, 5115–5120. 10.1063/1.432185.

[ref21] Marciniak H.; Pugliesi I.; Nickel B.; Lochbrunner S. Ultrafast Singlet and Triplet Dynamics in Microcrystalline Pentacene Films. Phys. Rev. B: Condens. Matter Mater. Phys. 2009, 79, 235318 10.1103/physrevb.79.235318.

[ref22] Marciniak H.; Fiebig M.; Huth M.; Schiefer S.; Nickel B.; Selmaier F.; Lochbrunner S. Ultrafast Exciton Relaxation in Microcrystalline Pentacene Films. Phys. Rev. Lett. 2007, 99, 176402 10.1103/physrevlett.99.176402. 17995352

[ref23] Thorsmølle V. K.; Averitt R. D.; Demsar J.; Smith D. L.; Tretiak S.; Martin R. L.; Chi X.; Crone B. K.; Ramirez A. P.; Taylor A. J. Photoexcited Carrier Relaxation Dynamics in Pentacene Probed by Ultrafast Optical Spectroscopy: Influence of Morphology on Relaxation Processes. Phys. B 2009, 404, 3127–3130. 10.1016/j.physb.2009.07.063.

[ref24] Rao A.; Wilson M. W. B.; Albert-Seifried S.; Di Pietro R.; Friend R. H. Photophysics of Pentacene Thin Films: The Role of Exciton Fission and Heating Effects. Phys. Rev. B: Condens. Matter Mater. Phys. 2011, 84, 195411 10.1103/physrevb.84.195411.

[ref25] Wilson M. W. B.; Rao A.; Clark J.; Kumar R. S. S.; Brida D.; Cerullo G.; Friend R. H. Ultrafast Dynamics of Exciton Fission in Polycrystalline Pentacene. J. Am. Chem. Soc. 2011, 133, 11830–11833. 10.1021/ja201688h. 21755937

[ref26] Johnson J. C.; Reilly T. H.; Kanarr A. C.; Van De Lagemaat J. The Ultrafast Photophysics of Pentacene Coupled to Surface Plasmon Active Nanohole Films. J. Phys. Chem. C 2009, 113, 6871–6877. 10.1021/jp901419s.

[ref27] Jundt C.; Klein G.; Sipp B.; Le Moigne J.; Joucla M.; Villaeys A. A. Exciton Dynamics in Pentacene Thin Films Studied by Pump-Probe Spectroscopy. Chem. Phys. Lett. 1995, 241, 84–88. 10.1016/0009-2614(95)00603-2.

[ref28] Budden P. J.; Weiss L. R.; Müller M.; Panjwani N. A.; Dowland S.; Allardice J. R.; Ganschow M.; Freudenberg J.; Behrends J.; Bunz U. H. F.; Friend R. H. Singlet Exciton Fission in a Modified Acene with Improved Stability and High Photoluminescence Yield. Nat. Commun. 2021, 12, 1527 10.1038/s41467-021-21719-x. 33750774 PMC7943798

[ref29] Arnold S. Optically Induced Fission Spectrum in Anthracene. J. Chem. Phys. 1974, 61, 431–432. 10.1063/1.1681662.

[ref30] Burdett J. J.; Müller A. M.; Gosztola D.; Bardeen C. J. Excited State Dynamics in Solid and Monomeric Tetracene: The Roles of Superradiance and Exciton Fission. J. Chem. Phys. 2010, 133, 144506 10.1063/1.3495764. 20950016

[ref31] Burdett J. J.; Gosztola D.; Bardeen C. J. The Dependence of Singlet Exciton Relaxation on Excitation Density and Temperature in Polycrystalline Tetracene Thin Films: Kinetic Evidence for a Dark Intermediate State and Implications for Singlet Fission. J. Chem. Phys. 2011, 135, 214508 10.1063/1.3664630. 22149803

[ref32] Arnold S.; Whitten W. B. Temperature Dependence of the Triplet Exciton Yield in Fission and Fusion in Tetracene. J. Chem. Phys. 1981, 75, 1166–1169. 10.1063/1.442164.

[ref33] Vaubel G.; Baessler H. Excitation Spectrum of Crystalline Tetracene Fluorescence: A Probe for Optically-Induced Singlet-Exciton Fission. Mol. Cryst. Liq. Cryst. 1971, 15, 15–25. 10.1080/15421407108083220.

[ref34] Vaubel G.; Baessler H. Diffusion of Singlet Excitons in Tetracene Crystals. Mol. Cryst. 1970, 12, 47–56. 10.1080/15421407008082759.

[ref35] Tomkiewicz Y.; Groff R. P.; Avakian P. Spectroscopic Approach to Energetics of Exciton Fission and Fusion in Tetracene Crystals. J. Chem. Phys. 1971, 54, 4504–4507. 10.1063/1.1674702.

[ref36] Swenberg C. E.; Ratner M. A.; Geacintov N. E. Energy Dependence of Optically Induced Exciton Fission. J. Chem. Phys. 1974, 60, 2152–2157. 10.1063/1.1681326.

[ref37] Carlotti B.; Madu I. K.; Kim H.; Cai Z.; Jiang H.; Muthike A. K.; Yu L.; Zimmerman P. M.; Goodson T. Activating Intramolecular Singlet Exciton Fission by Altering π-Bridge Flexibility in Perylene Diimide Trimers for Organic Solar Cells. Chem. Sci. 2020, 11, 8757–8770. 10.1039/d0sc03271a. 34123128 PMC8163386

[ref38] Schierl C.; Niazov-Elkan A.; Shimon L. J. W.; Feldman Y.; Rybtchinski B.; Guldi D. M. Singlet Fission in Self-Assembled PDI Nanocrystals. Nanoscale 2018, 10, 20147–20154. 10.1039/c8nr04155e. 30221262

[ref39] Farag M. H.; Krylov A. I. Singlet Fission in Perylenediimide Dimers. J. Phys. Chem. C 2018, 122, 25753–25763. 10.1021/acs.jpcc.8b05309.

[ref40] Margulies E. A.; Miller C. E.; Wu Y.; Ma L.; Schatz G. C.; Young R. M.; Wasielewski M. R. Enabling Singlet Fission by Controlling Intramolecular Charge Transfer in π-Stacked Covalent Terrylenediimide Dimers. Nat. Chem. 2016, 8, 1120–1125. 10.1038/nchem.2589. 27874873

[ref41] Blaskovits J. T.; Fumanal M.; Vela S.; Fabregat R.; Corminboeuf C. Identifying the Trade-off between Intramolecular Singlet Fission Requirements in Donor-Acceptor Copolymers. Chem. Mater. 2021, 33, 2567–2575. 10.1021/acs.chemmater.1c00057.

[ref42] Hu J.; Xu K.; Shen L.; Wu Q.; He G.; Wang J. Y.; Pei J.; Xia J.; Sfeir M. Y. New Insights into the Design of Conjugated Polymers for Intramolecular Singlet Fission. Nat. Commun. 2018, 9, 2999 10.1038/s41467-018-05389-w. 30065295 PMC6068183

[ref43] Wang L.; Liu X.; Shi X.; Anderson C. L.; Klivansky L. M.; Liu Y.; Wu Y.; Chen J.; Yao J.; Fu H. Singlet Fission in a Para-Azaquinodimethane-Based Quinoidal Conjugated Polymer. J. Am. Chem. Soc. 2020, 142, 17892–17896. 10.1021/jacs.0c06604. 33044060

[ref44] Zhai Y.; Sheng C.; Vardeny Z. V. Singlet Fission of Hot Excitons in π-Conjugated Polymers. Philos. Trans. R. Soc., A 2015, 373, 20140327 10.1098/rsta.2014.0327. PMC4455724 25987576

[ref45] Fumanal M.; Corminboeuf C. Direct, Mediated, and Delayed Intramolecular Singlet Fission Mechanism in Donor-Acceptor Copolymers. J. Phys. Chem. Lett. 2020, 11, 9788–9794. 10.1021/acs.jpclett.0c03076. 33147966

[ref46] Masoomi-Godarzi S.; Liu M.; Tachibana Y.; Goerigk L.; Ghiggino K. P.; Smith T. A.; Jones D. J. Solution-Processable, Solid State Donor–Acceptor Materials for Singlet Fission. Adv. Energy Mater. 2018, 8, 1801720 10.1002/aenm.201801720.

[ref47] Fumanal M.; Corminboeuf C. Pushing the Limits of the Donor-Acceptor Copolymer Strategy for Intramolecular Singlet Fission. J. Phys. Chem. Lett. 2021, 12, 7270–7277. 10.1021/acs.jpclett.1c01986. 34318679

[ref48] Wang C.; Schlamadinger D. E.; Desai V.; Tauber M. J. Triplet Excitons of Carotenoids Formed by Singlet Fission in a Membrane. ChemPhysChem 2011, 12, 2891–2894. 10.1002/cphc.201100571. 21910205

[ref49] Wang C.; Tauber M. J. High-Yield Singlet Fission in a Zeaxanthin Aggregate Observed by Picosecond Resonance Raman Spectroscopy. J. Am. Chem. Soc. 2010, 132, 13988–13991. 10.1021/ja102851m. 20857932

[ref50] Fallon K. J.; Budden P.; Salvadori E.; Ganose A. M.; Savory C. N.; Eyre L.; Dowland S.; Ai Q.; Goodlett S.; Risko C.; Scanlon D. O.; Kay C. W. M.; Rao A.; Friend R. H.; Musser A. J.; Bronstein H. Exploiting Excited-State Aromaticity to Design Highly Stable Singlet Fission Materials. J. Am. Chem. Soc. 2019, 141, 13867–13876. 10.1021/jacs.9b06346. 31381323

[ref51] Fallon K. J.; Bronstein H. Indolonaphthyridine: A Versatile Chromophore for Organic Electronics Inspired by Natural Indigo Dye. Acc. Chem. Res. 2021, 54, 182–193. 10.1021/acs.accounts.0c00601. 33297676

[ref52] Engi G. Über Neue Derivate Des Indigos Und Anderer Indigoider Farbstoffe. Zeitschrift für Angew. Chemie 1914, 27, 144–148. 10.1002/ange.19140272003.

[ref53] Smith M. B.; Michl J. Singlet Fission. Chem. Rev. 2010, 110, 6891–6936. 10.1021/cr1002613. 21053979

[ref54] Ryerson J. L.; Zaykov A.; Aguilar Suarez L. E.; Havenith R. W. A.; Stepp B. R.; Dron P. I.; Kaleta J.; Akdag A.; Teat S. J.; Magnera T. F.; Miller J. R.; Havlas Z.; Broer R.; Faraji S.; Michl J.; Johnson J. C. Structure and Photophysics of Indigoids for Singlet Fission: Cibalackrot. J. Chem. Phys. 2019, 151, 184903 10.1063/1.5121863. 31731849

[ref55] Yao Z. F.; Wang J. Y.; Pei J. Control of π–π Stacking via Crystal Engineering in Organic Conjugated Small Molecule Crystals. Cryst. Growth Des. 2018, 18, 7–15. 10.1021/acs.cgd.7b01385.

[ref56] Ferraris J.; Cowan D. O.; Walatka V.; Perlstein J. H. Electron transfer in a new highly conducting donor-acceptor complex. J. Am. Chem. Soc. 1973, 95, 948–949. 10.1021/ja00784a066.

[ref57] Zhang J.; Xu W.; Sheng P.; Zhao G.; Zhu D. Organic Donor-Acceptor Complexes as Novel Organic Semiconductors. Acc. Chem. Res. 2017, 50, 1654–1662. 10.1021/acs.accounts.7b00124. 28608673

[ref58] Sokolov A. N.; Friščić T.; MacGillivray L. R. Enforced Face-to-Face Stacking of Organic Semiconductor Building Blocks within Hydrogen-Bonded Molecular Cocrystals. J. Am. Chem. Soc. 2006, 128, 2806–2807. 10.1021/ja057939a. 16506752

[ref59] Anthony J. E. The Larger Acenes: Versatile Organic Semiconductors. Angew. Chem., Int. Ed. 2008, 47, 452–483. 10.1002/anie.200604045. 18046697

[ref60] Dou J. H.; Zheng Y. Q.; Yao Z. F.; Yu Z. A.; Lei T.; Shen X.; Luo X. Y.; Sun J.; Zhang S. D.; Ding Y. F.; Han G.; Yi Y.; Wang J. Y.; Pei J. Fine-Tuning of Crystal Packing and Charge Transport Properties of BDOPV Derivatives through Fluorine Substitution. J. Am. Chem. Soc. 2015, 137, 15947–15956. 10.1021/jacs.5b11114. 26619351

[ref61] Liang Z.; Tang Q.; Xu J.; Miao Q. Soluble and Stable N-Heteropentacenes with High Field-Effect Mobility. Adv. Mater. 2011, 23, 1535–1539. 10.1002/adma.201004325. 21449057

[ref62] Sucharda E. Über Eine Neue Darstellungsmethode Der Chinolinsäure Und Einiger Derivate Derselben. Berichte der Dtsch. Chem. Gesellschaft A B Ser. 1925, 58, 1727–1729. 10.1002/cber.19250580858.

[ref63] Kolaczkowski M. A.; He B.; Liu Y. Stepwise Bay Annulation of Indigo for the Synthesis of Desymmetrized Electron Acceptors and Donor-Acceptor Constructs. Org. Lett. 2016, 18, 5224–5227. 10.1021/acs.orglett.6b02504. 27723358

[ref64] Spano F. C. The Spectral Signatures of Frenkel Polarons in H- And J-Aggregates. Acc. Chem. Res. 2010, 43, 429–439. 10.1021/ar900233v. 20014774

[ref65] Spano F. C.; Silva C. H- and J-Aggregate Behavior in Polymeric Semiconductors. Annu. Rev. Phys. Chem. 2014, 65, 477–500. 10.1146/annurev-physchem-040513-103639. 24423378

[ref66] Musser A. J.; Maiuri M.; Brida D.; Cerullo G.; Friend R. H.; Clark J. The Nature of Singlet Exciton Fission in Carotenoid Aggregates. J. Am. Chem. Soc. 2015, 137, 5130–5139. 10.1021/jacs.5b01130. 25825939 PMC4440407

[ref67] Purdy M.; Fallon K.; Congrave D. G.; Toolan D. T. W.; Zeng W.; Bronstein H. Synthesis of Asymmetric Indolonaphthyridines with Enhanced Excited State Charge-Transfer Character. J. Mater. Chem. C 2022, 10, 10742–10747. 10.1039/d1tc06054f.

[ref68] De Mello J. C.; Wittmann H. F.; Friend R. H. An Improved Experimental Determination of External Photoluminescence Quantum Efficiency. Adv. Mater. 1997, 9, 230–232. 10.1002/adma.19970090308.

[ref69] Nielsen B. R.; Jørgensen K.; Skibsted L. H. Triplet—triplet extinction coefficients, rate constants of triplet decay and rate constant of anthracene triplet sensitization by laser flash photolysis of astaxanthin, β-carotene, canthaxanthin and zeaxanthin in deaerated toluene at 298 K. J. Photochem. Photobiol., A 1998, 112, 127–133. 10.1016/s1010-6030(97)00285-2.

[ref70] Jones R. N. The Ultraviolet Absorption Spectra of Anthracene Derivatives. Chem. Rev. 1947, 41, 353–371. 10.1021/cr60129a013. 18901150

[ref71] Rao A.; Chow P. C. Y.; Gélinas S.; Schlenker C. W.; Li C. Z.; Yip H. L.; Jen A. K. Y.; Ginger D. S.; Friend R. H. The Role of Spin in the Kinetic Control of Recombination in Organic Photovoltaics. Nature 2013, 500, 435–439. 10.1038/nature12339. 23925118

[ref72] Gélinas S.; Paré-Labrosse O.; Brosseau C. N.; Albert-Seifried S.; McNeill C. R.; Kirov K. R.; Howard I. A.; Leonelli R.; Friend R. H.; Silva C. The Binding Energy of Charge-Transfer Excitons Localized at Polymeric Semiconductor Heterojunctions. J. Phys. Chem. C 2011, 115, 7114–7119. 10.1021/jp200466y.

[ref73] Zeng W.; El Bakouri O.; Szczepanik D. W.; Bronstein H.; Ottosson H. Excited State Character of Cibalackrot-Type Compounds Interpreted in Terms of Hückel-Aromaticity: A Rationale for Singlet Fission Chromophore Design. Chem. Sci. 2021, 12, 6159–6171. 10.1039/d1sc00382h. 33996014 PMC8098681

[ref74] Fallon K. J.; Wijeyasinghe N.; Yaacobi-Gross N.; Ashraf R. S.; Freeman D. M. E.; Palgrave R. G.; Al-Hashimi M.; Marks T. J.; McCulloch I.; Anthopoulos T. D.; Bronstein H. A Nature-Inspired Conjugated Polymer for High Performance Transistors and Solar Cells. Macromolecules 2015, 48, 5148–5154. 10.1021/acs.macromol.5b00542.

[ref75] Lu T.; Chen F. Multiwfn A Multifunctional Wavefunction Analyzer. J. Comput. Chem. 2012, 33, 580–592. 10.1002/jcc.22885. 22162017

