# Supplementary material for: Aza-Cibalackrot: Turning on Singlet Fission Through Crystal Engineering
Source: J Am Chem Soc. 2023 May 3;145(19):10712–20. doi: 10.1021/jacs.3c00971 (PMC10197122; doi:10.1021/jacs.3c00971)
Supplement: Supplementary file 1 — ja3c00971_si_001.pdf [file ja3c00971_si_001.pdf]

Aza-Cibalackrot: Turning on Singlet Fission Through Crystal Engineering

Michael Purdy,[†] Jessica R. Walton,[‡] Kealan J. Fallon,[†] Daniel T. W. Toolan,[‖] Peter Budden,[‡] Weixuan Zeng,[†] Merina K. Corpinot,[§] Dejan-Krešimir Bučar, Lars van Turnhout, [‡ §] Richard Friend,[‡] Akshay Rao,[‡*] Hugo Bronstein[†*]

† Yusuf Hamied Department of Chemistry, University of Cambridge, Lensfield Rd, Cambridge CB2 1EW, UK

‡ Department of Physics, Cavendish Laboratory, JJ Thomson Avenue, Cambridge, CB3 0HE, UK

§ Department of Chemistry, University College London, 20 Gordon Street, London, WC1H 0AJ, UK

‖ Department of Chemistry, University of Sheffield, Dainton Building, Brook Hill, Sheffield, S3 7HF, U.K.

Table of Contents

Synthesis 2
NMR spectra 12
Single crystal X-ray diffraction measurements 42
GIWAXS 44
Optical Spectroscopy Methods 48
Steady State Characterisation 50
Transient Photoluminescence 51
Transient Absorption Spectroscopy (Film Excitation Density Dependence) 52
Transient Absorption Spectroscopy (Solutions) 54
Triplet sensitization – Solution 56
Triplet sensitization – Film (PdTPTBP sensitizer) 61
Spectral shifts of T_1 state (INDTs – from literature) 69
Species 2 (TT) yield estimation 70
References 74

Synthesis

General

^1H NMR spectra were recorded on a 400 MHz Avance III HD Spectrometer in the stated solvent using residual protic solvent as the internal standard. ^1H NMR chemical shifts are reported to the nearest 0.01 ppm. The coupling constants (J) are measured in Hertz. ^{13}C NMR spectra were recorded on the 500 MHz DCH Cryoprobe Spectrometer in the stated solvent using the residual protic solvent as the internal standard. ^{13}C NMR chemical shifts are reported to the nearest 0.01 ppm. Mass spectra were obtained using a Waters LCT, Finnigan MAT 900XP or Waters MALDI micro MX spectrometer at the Department of Chemistry, University of Cambridge. Reactions requiring an inert atmosphere were carried out under argon. Thin layer chromatography (TLC) was carried out on silica gel and visualized using UV light (254, 365 nm). Flash chromatography was carried out on a Biotage® Isolera automated flash chromatography machine on 60 micron silica gel cartridges purchased from Biotage®.

Chemicals

All commercial chemicals were of ≥95% purity and were used as received without further purification. Anhydrous solvents were purchased from Sigma Aldrich or Acros Organics and used as received.

Alkoxy phenylacetyl chloride synthesis

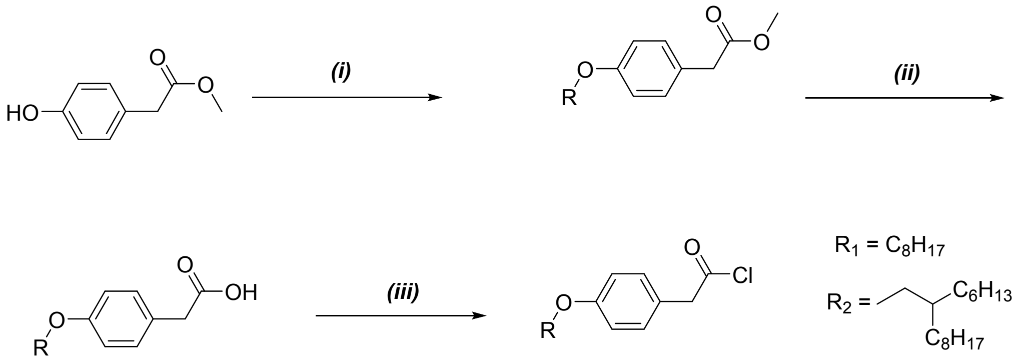

Scheme S1. *Synthesis of alkoxy phenylacetyl chlorides*. Reagents and conditions: (i) bromoalkane (1.5 equiv), K_2CO_3 (3 equiv), DMF, 100 ° C, 12 h (R_1 = 67 %, R_2= 64 %). (ii) 10 % NaOH solution (10 equiv), THF, r.t., 12 h h (R_1 = 98 %, R_2= 98 %). (iii) Thionyl chloride (3 equiv), DMF (1 drop), DCM, reflux, 3 h (R_1 = 82 %, R_2= 82 %).

Procedures

*Preparation of **1**-**3** was based on literature route and NMR data obtained in this work was in full accordance with what had been reported.*[1] *Acid chloride **6** could not be successfully found using mass spectrometry likely due to instability of molecule. ^{13}C spectra of **11** and **13** could not be obtained due to aggregation at required concentration for analysis.*

Methyl 2-(4-((2-hexyldecyl)oxy)phenyl)acetate (1)

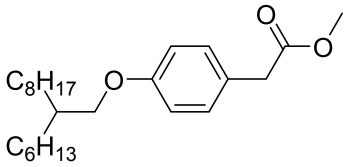

Methyl 2-(4-hydroxyphenyl)acetate (9.00 g, 54.0 mmol) was added to a mixture of potassium carbonate (22.5 g, 162 mmol) and 7-(bromomethyl)pentadecane (24.6 g, 81.2 mmol) in DMF (300 mL) under inter atmosphere. The reaction heated to 100 °C for 12 h then cooled to room temperature and extracted using chloroform (500 mL). The organic layer was washed with water (200 mL) followed by brine (×2 200 mL) and then dried over anhydrous magnesium sulfate. The solvent was removed under reduced pressure yielding a yellow oil which was then purified by silica column chromatography, using an eluent of 10 % EtOAC:Hexane, leaving the product as a colourless oil (13.5 g, 34.6 mmol, 64 %). ^{1}H NMR (400 MHz, CDCl3) 7.17 (d, *J* = 8.7 Hz, 2H), 6.85 (d, *J* = 8.7 Hz, 2H), 3.81 (d, *J* = 4.0 Hz, 2H), 3.68 (s, 3H), 3.56 (s, 2H), 1.83-1.78 (m, 1H), 1.27-1.42 (m, 24H), 0.88 (t, *J* = 8.0 Hz, 6H).

2-(4-((2-Hexyldecyl)oxy)phenyl)acetic acid (2)

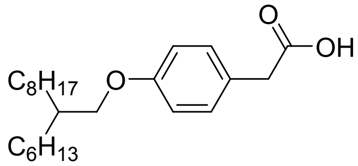

1 (13.5 g, 34.6 mmol) was dissolved in THF (100 mL) and 10 % NaOH solution (150 mL) was added with stirring. The reaction was stirred over night at room temperature and then the pH was reduced to 3 by adding concentrated HCl. The acidic solution was stirred for 3 hours and then an organic extraction using chloroform (500 mL) was performed. The organic layer was washed with brine (×2 200 mL) and then dried over anhydrous magnesium sulfate, the solvent was removed under reduced pressure yielding the product as a pale yellow oil (12.8 g, 34.0 mmol, 98 %). ^{1}H NMR (400 MHz, CDCl3) 7.18 (d, *J* = 8.2 Hz, 2H), 6.86 (d, *J* = 8.2 Hz, 2H), 3.81 (d, *J* = 5.6 Hz, 2H), 3.59 (s, 2H), 1.84-1.87 (m, 1H), 1.27-1.44 (m, 24H), 0.88 (t, *J* = 6.4 Hz, 6H).

2-(4-((2-Hexyldecyl)oxy)phenyl)acetyl chloride (3)

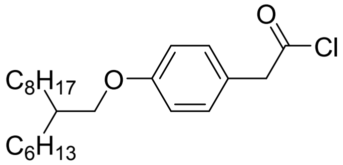

Thionyl chloride (12.2 g, 102 mmol) and anhydrous DMF (1 drop) were added to a solution of **2** (12.8 g, 34.0 mmol) in anhydrous DCM (20 mL) under inert atmosphere. The reaction was stirred and heated under reflux for 2 hours and then allowed to cool the room temperature. The solvent and excess thionyl chloride were removed under reduced pressure yielding **3** as a yellow oil (11.0 g, 27.8 mmol, 82 %). ^{1}H NMR (400 MHz, CDCl3) δ 7.17 (d, *J* = 8.2 Hz, 2H), 6.88 (d, *J* = 8.2 Hz, 2H), 4.07 (d, *J* = 5.6 Hz, 2H), 3.82 (s, 2H), 1.74-1.79 (m, 1H), 1.28-1.38 (m, 24H), 0.88 (t, *J* = 6.4 Hz, 6H).

Methyl 2-(4-(octyloxy)phenyl)acetate (4)

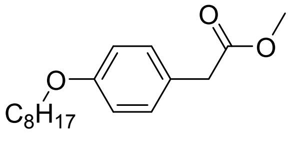

Methyl 2-(4-hydroxyphenyl)acetate (9.00 g, 54.0 mmol) was added to a mixture of potassium carbonate (22.5 g, 162 mmol) and 1-bromooctane (15.6 g, 81.1 mmol) in DMF (300 mL) under intert atmosphere. The reaction heated to 100 °C for 12 h then cooled to room temperature and extracted using chloroform (500 mL). The organic layer was washed with water (×2 200 mL) followed by brine (×2 200 mL) and then dried over anhydrous magnesium sulfate. The solvent was removed under reduced pressure yielding a yellow oil which was then purified by silica column chromatography, using an eluent of 10 % EtOAC:Hexane, leaving the product as a colourless oil (10.1 g, 36.3 mmol, 67 %). ^{1}H NMR (400 MHz, CDCl3) 7.17 (d, *J* = 8.7 Hz, 2H), 6.85 (d, *J* = 8.7 Hz, 2H), 3.93 (t, *J* = 6.6 Hz, 2H) 3.68 (s, 3H), 3.56 (s, 2H), 1.80-1.78 (m, 2H), 1.48-1.36 (m, 2H), 1.36-1.24 (m, 8H), 0.89 (t, *J* = 6.9 Hz, 3H). ^{13}C NMR (150 MHz, CDCl3) δ 172.6, 158.5, 130.4, 126.0, 114.8, 68.2, 52.2, 40.5, 32.0, 29.6, 29.5, 29.4, 26.3, 22.9, 14.3. HMRS Found (TOF MS+): $[M]^+$ 279.1951 $C_{17}H_{27}O_3$ requires 279.1960.

2-(4-(Octyloxy)phenyl)acetic acid (5)

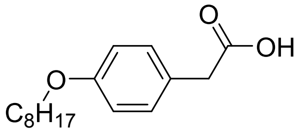

4 (10.1 g, 36.3 mmol) was dissolved in THF (100 mL) and 10 % NaOH solution (150 mL) was added with stirring. The reaction was stirred over night at room temperature and then the pH was reduced to 3 by adding concentrated HCl. The acidic solution was stirred for 3 hours and then an organic extraction using chloroform (500 mL) was performed. The organic layer was washed with brine (×2 200 mL) and then dried over anhydrous magnesium sulfate, the solvent was removed under reduced pressure yielding the product as a pale yellow solid (9.41 g, 35.6 mmol, 98 %). ^{1}H NMR (400 MHz, CDCl3) 7.18 (d, *J* = 8.7 Hz, 2H), 6.86 (d, *J* = 8.7 Hz, 2H), 3.93 (t, *J* = 6.6 Hz, 2H) 3.58 (s, 2H), 1.80-1.73 (m, 2H), 1.48-1.40 (m, 2H), 1.36-1.28 (m, 8H), 0.89 (t, *J* = 6.9 Hz, 3H). ^{13}C NMR (150 MHz, CDCl3) δ 178.1, 158.6, 130.5, 125.3, 114.8, 68.2, 40.3, 32.0, 31.1, 29.5, 29.4 (2C), 26.2, 22.8, 14.3. HRMS Found (TOF MS+): $[M]^+$ 265.1799 $C_{16}H_{25}O_3$ requires 265.1804.

2-(4-(Octyloxy)phenyl)acetyl chloride (6)

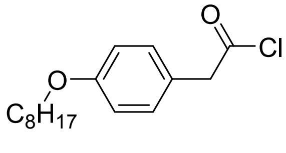

Thionyl chloride (12.7 g, 107 mmol) and anhydrous DMF (1 drop) were added to a solution of **5** (9.41 g, 35.6 mmol) in anhydrous DCM (20 mL) under inert atmosphere. The reaction was stirred and heated under reflux for 2 hours and then allowed to cool the room temperature. The solvent and excess thionyl chloride was removed under reduced pressure yielding the product as a yellow oil (11.0 g, 27.8 mmol, 82 %). ^{1}H NMR (400 MHz, CDCl3) δ 7.17 (d, *J* = 8.7 Hz, 2H), 6.88 (d, *J* = 8.7 Hz, 2H), 4.07 (s, 2H), 3.95 (t, *J* = 6.6 Hz, 2H), 1.81-1.75 (m, 2H), 1.48-1.40 (m, 2H), 1.36-1.28 (m, 8H), 0.89 (t, *J* = 6.9 Hz, 3H). ^{13}C NMR (150 MHz, CDCl3) δ 172.5, 159.2, 130.8, 123.2, 115.1, 68.3, 52.5, 32.0, 29.5, 29.4, 26.2, 22.8, 14.3.

3-((Carboxymethyl)amino)picolinic acid (7)

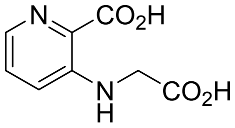

3–aminopicolinic acid (24.0 g, 170 mmol) was added to a solution of potassium carbonate (37.0 g, 270 mmol) in distilled water (570 mL). After one hour of stirring, 2-chloroacetic acid (17.7 g, 187 mmol) was added and the reaction was refluxed for 24 hours. The reaction was allowed to cool to room temperature and concentrated HCl was added with stirring in 2 mL portions until a pH of 3 was achieved. The suspension was filtered and washed with methanol. A pale cream residue was collected and recrystallised in ethanol, the solvent was then removed via filtration and 30 was collected as a white solid (9.44 g, 18 %). ^{1}H NMR (400 MHz, (CD3)2SO) δ 8.29 (s, 1H, br), 7.89 (d, *J* = 3.3, 1H), 7.46 (dd, *J* = 8.6, *J* = 3.3, 1H), 7.23 (d, *J* = 8.6, 1H) 4.07 (s, 2H), 3.48 (s, 2H, br). ^{13}C NMR (100 MHz, DMSO) δ 171.4, 167.5, 146.6, 134.2, 128.6, 127.2, 121.1, 43.6. HRMS Found (TOF MS+): [M]$^+$ 197.0564 $C_{16}H_{25}O_3$ requires 197.0562.

1-Acetyl-1*H*-pyrrolo[3,2-*b*]pyridin-3-yl acetate (8)

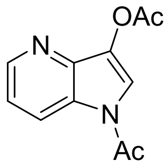

Fused potassium acetate (11.8 g, 120 mmol) was added to a solution of 3((carboxymethyl)amino)picolinic acid (9.44 g, 48.1 mmol) in acetic anhydride (64 mL) under inert atmosphere. The solution was heated to reflux and stirred until effervescence ceased. The reaction was cooled to room temperature producing dark brown scum which was then filtered. The residue was washed with acetic anhydride and the filtrate collected. The solvent was removed under reduced pressure leaving a dark brown solid which was then taken up in ethyl acetate and passed through a small silica plug. Yellow fractions were collected, and the solvent removed under reduced pressure yielding a yellow crystalline solid which was then taken up in methanol and filtered, the filtrate was collected, and the solvent removed under reduced pressure yielding **8** as a pale yellow crystalline solid (8.65 g, 83 %). ^{1}H NMR (400 MHz, CDCl3) δ 8.70 (d, *J* = 8.1 Hz, 1H), 8.60 (d, *J* = 4.7, 1H), 7.98 (s, 1H), 7.33 (dd, *J* = 8.1, J = 4.7, 1H), 2.62 (s, 3H), 2.43 (s, 3H). NMR (150 MHz, CDCl3) δ ^{13}C NMR (100 MHz, $CDCl_3$) δ 168.9, 168.2, 146.8, 140.8, 134.0, 127.0, 124.4, 121.0, 117.1, 23.5, 21.3. HRMS Found (TOF MS+): $[M]^+$ 219.0777 $C_{11}H_{11}N_2O_3$ requires 219.0770.

(*E*)-[2,2'-Bipyrrolo[3,2-*b*]pyridinylidene]-3,3'(1*H*,1'*H*)-dione (9)

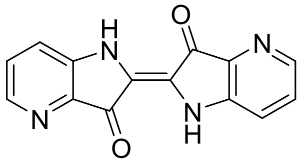

Ammonia solution (220 mL) was added to **2** (8.65 g, 39.7 mmol) in ten portions with stirring forming a dark green viscous solution. After 18 hours, methanol (100 mL) was added and the solution filtered. The residue was collected and treated with a small amount of hot DMSO, re-filtered and then washed extensively with methanol followed by acetone and air-dried yielding **9** as a dark blue solid (2.10 g, 7.94 mmol, 20 %). ^{1}H NMR (400 MHz, (CD3)2SO) δ 10.79 (s, 2H), 8.30 (d, *J* = 4.4 Hz, 2H), 7.75 (d, *J* = 8.1 Hz, 2H), 7.45 (dd, *J* = 4.4 Hz & *J* = 8.1 Hz, 2H). HRMS Found (TOF MS+): $[M]^+$ 265.0726 $C_{14}H_9N_4O_2$ requires 265.0726.

7,14-Bis(4-((2-hexyldecyl)oxy)phenyl)pyrido[2',3':4,5]pyrrolo[3,2,1-*de*]pyrido[2',3':4,5]pyrrolo[3,2,1-*ij*][1,5]naphthyridine-6,13-dione (10)

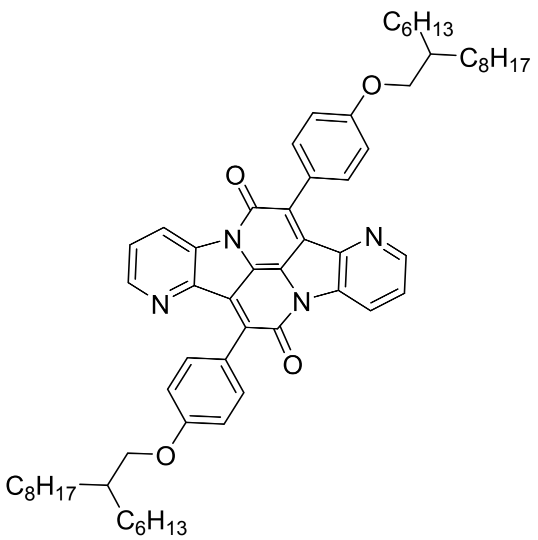

9 (760 mg, 2.89 mmol) was dissolved in xylene (38 mL) and heated to reflux. A solution of acid Chloride **3** (11.4 g, 28.9 mmol) was dissolved in xylene (5 mL) and added dropwise to the refluxing solution. After 16 hr, the reaction was cooled to room temperature and the xylenes removed under vacuum leaving a dark brown solid which was placed onto a silica plug and eluted with neat ethyl acetate. The filtrate was collected, and the solvent removed under reduced pressure producing a black solid which was dry loaded onto a silica column and eluted with 10 % EtOAc:Hexane. Fractions containing a deep purple solution were collected and the solvent removed under reduced pressure producing a dark purple solid which was sonicated in methanol producing a solid suspension. After filtration, **10** was air-dried and collected as a black waxy solid (15 mg, 0.02 mmol, 0.6 %). ^{1}H NMR (400 MHz, CDCl3) δ 8.79 (dd, J = 8.2 Hz & J = 1.5 Hz, 2H), 8.58 (dd, J = 4.9 Hz & J = 1.5 Hz, 2H), 8.00 (d, J = 8.8 Hz, 4H), 7.44 – 7.45 (dd, J = 8.2 Hz & J = 4.9 Hz, 2H), 7.06 (d, J = 8.8 Hz, 4H), 3.95 (d, J = 5.5 Hz, 4H), 1.80-1.86 (m, 2H), 1.25-1.38 (m, 48H), 0.85 – 0.92 (m, 12H). ^{13}C NMR (100 MHz, $CDCl_3$) δ 160.8, 159.7, 147.6, 146.0, 139.2, 133.20, 132.80, 128.8, 124.7, 124.6, 124.5, 121.5, 114.0, 68.2, 32.0, 29.9 (2C), 29.6, 29.5, 26.3, 22.9, 14.3. HRMS Found (TOF MS+): $[M]^+$ 945.6266 $C_{62}H_{81}N_4O_4$ requires 945.6258.

7,14-Bis(4-(octyloxy)phenyl)pyrido[2',3':4,5]pyrrolo[3,2,1-*de*]pyrido[2',3':4,5]pyrrolo[3,2,1-*ij*][1,5]naphthyridine-6,13-dione (11)

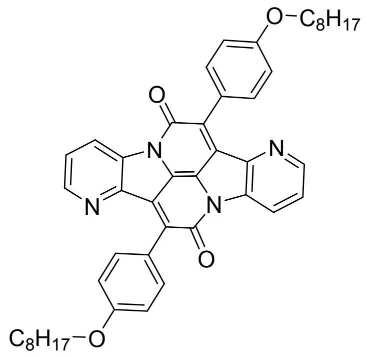

9 (760 mg, 2.89 mmol) was dissolved in xylene (38 mL) and heated to reflux. A solution of acid Chloride **6** (8.15 g, 28.9 mmol), dissolved in xylene (5 mL), was added dropwise to the refluxing solution. After 16 hr, the reaction was cooled to room temperature and the xylenes removed under vacuum leaving a dark brown solid which was placed onto a silica plug and eluted with DCM. Once the filtrate lost its' dark brown colour the eluent was changed to 50:50 EtOAc:DCM and dark purple fractions were collected. The solvent was removed under reduced pressure leaving a black residue was triturated from acetone. The solid was then filtered, air-dried and **11** was collected as a black waxy solid (11 mg, 0.015 mmol, 0.4 %). ^{1}H NMR (400 MHz, CDCl3) δ 8.79 (dd, *J* = 8.2 Hz & *J* = 1.5 Hz, 2H), 8.58 (dd, *J* = 4.9 Hz & *J* = 1.5 Hz, 2H), 8.00 (d, *J* = 8.8 Hz, 4H), 7.44 – 7.45 (dd, *J* = 8.2 Hz & *J* = 4.9 Hz, 2H), 7.06 (d, *J* = 8.8 Hz, 4H), 4.08 (t, *J* = 6.4 Hz, 4H), 1.88-1.81 (m, 4H), 1.52-1.46 (m, 4H), 1.42-1.27 (m, 16H), 0.91 (t, *J* = 6.3 Hz, 6H). HRMS Found (TOF MS+): $[M]^+$ 721.3785 $C_{46}H_{49}N_4O_4$ requires 721.3754.

7,14-Bis(4-((2-hexyldecyl)oxy)phenyl)diindolo[3,2,1-*de*:3',2',1'-*ij*][1,5]naphthyridine-6,13-dione (12)

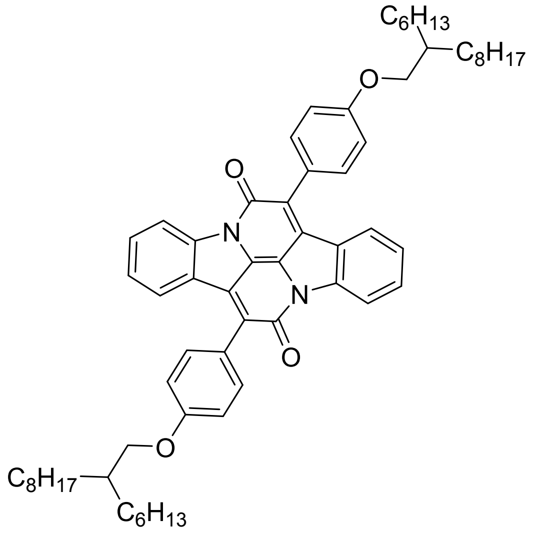

Indigo (247 mg, 0.94 mmol) was dissolved in xylene (60 mL) and heated to reflux. A solution of acid Chloride **3** (14.2 g, 36.0 mmol) was dissolved in xylene (5 mL) and added dropwise to the refluxing solution. After 16 hr, the reaction was cooled to room temperature and the xylenes removed under vacuum leaving a dark brown solid which was placed onto a silica plug and eluted with neat chloroform. The filtrate was collected, and the solvent removed under reduced pressure producing a black solid which was dry loaded onto a silica column and eluted with 20 % chloroform:hexane. Fractions containing a deep orange solution were collected and the solvent removed under reduced pressure producing a dark red solid which was sonicated in methanol producing a solid suspension. After filtration, **12** was air-dried and

collected as a red waxy solid (45 mg, 0.05 mmol, 5 %). ^{1}H NMR (400 MHz, CDCl3) δ 8.51 (d, *J* = 8.1, 2H), 7.61 – 7.67 (m, 6H), 7.55 (t, *J* = 7.8, 2H), 7.22 (t, *J* = 7.8, 2H), 7.09 (d, *J* = 8.7 Hz, 4H), 3.96 (d, *J* = 5.6 Hz, 4H), 1.83 – 1.88 (m, 2H), 1.27 – 1.52 (m, 48), 0.85 – 0.92 (m, 12H). ^{13}C NMR (126 MHz, $CDCl_3$) δ 207.2, 160.4, 160.0, 144.8, 132.0, 131.8, 131.5, 131.1, 126.2, 126.1, 125.6 (2C), 122.2, 117.8, 114.8, 71.3, 38.2, 32.1 (2C), 31.6, 31.1, 30.3, 29.9 (2C), 29.8, 29.6, 27.1 (2C), 22.9, 14.3. HRMS Found (TOF MS+): $[M]^+$ 943.6368 $C_{64}H_{83}N_2O_4$ requires 943.6353.

7,14-Bis(4-(octyloxy)phenyl)diindolo[3,2,1-*de*:3',2',1'-*ij*][1,5]naphthyridine-6,13-dione (13)

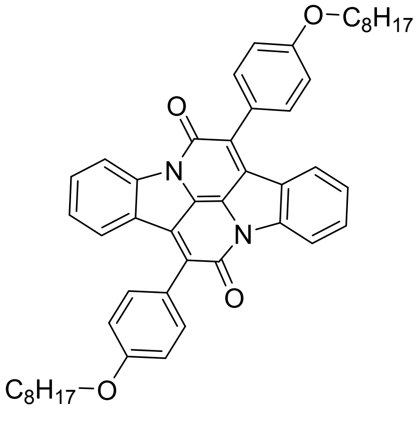

Indigo (366 mg, 1.40 mmol) was dissolved in xylene (60 mL) and heated to reflux. A solution of acid Chloride **6** (10.2 g, 36.0 mmol) was dissolved in xylene (5 mL) and added dropwise to the refluxing solution. After 16 hr, the reaction was cooled to room temperature and the xylenes removed under vacuum leaving a dark brown solid which was placed onto a silica plug and eluted with neat chloroform. The bright red filtrate was collected, and the solvent removed under reduced pressure producing a dark red residue which was triturated from acetone. The suspension was filtered, air-dried and **13** was collected as a red solid (321 mg, 0.45 mmol, 32 %). ^{1}H NMR (400 MHz, CDCl3) δ 8.51 (d, *J* = 8.1, 2H), 7.61 – 7.67 (m, 6H), 7.55 (t, *J* = 7.8, 2H), 7.22 (t, *J* = 7.8, 2H), 7.09 (d, *J* = 8.7 Hz, 4H), 4.08 (t, *J* = 6.3 Hz, 4H), 1.90 – 1.83 (m, 2H), 1.54 – 1.48 (m, 2H), 1.27 – 1.52 (m, 16), 0.91 (t, *J* = 6.2 Hz, 6H). HRMS Found (TOF MS+): $[M]^+$ 719.3871 $C_{48}H_{51}N_2O_4$ requires 719.3849.

NMR spectra

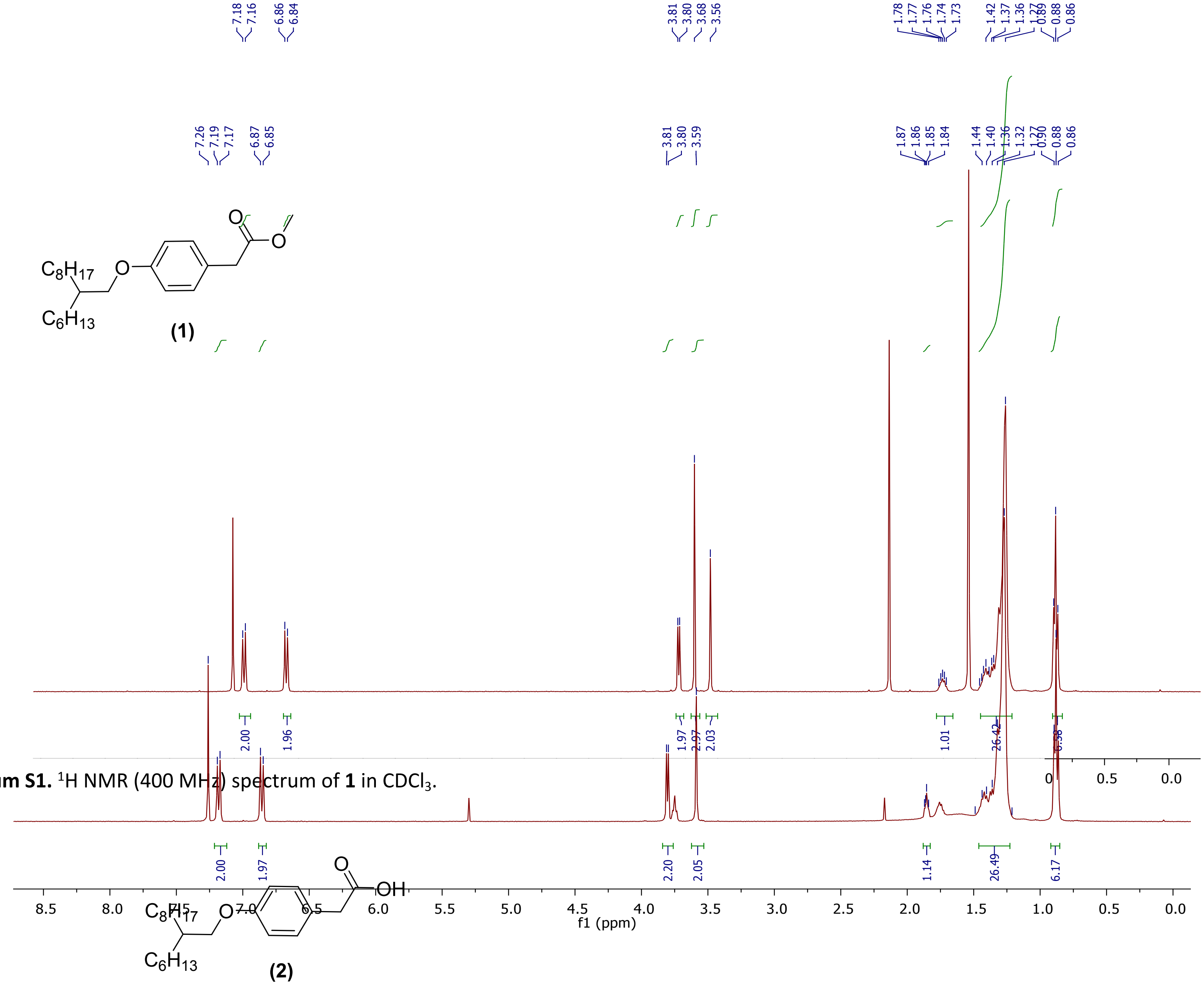

Spectrum S1. ^{1}H NMR (400 MHz) spectrum of **1** in $CDCl_3$.

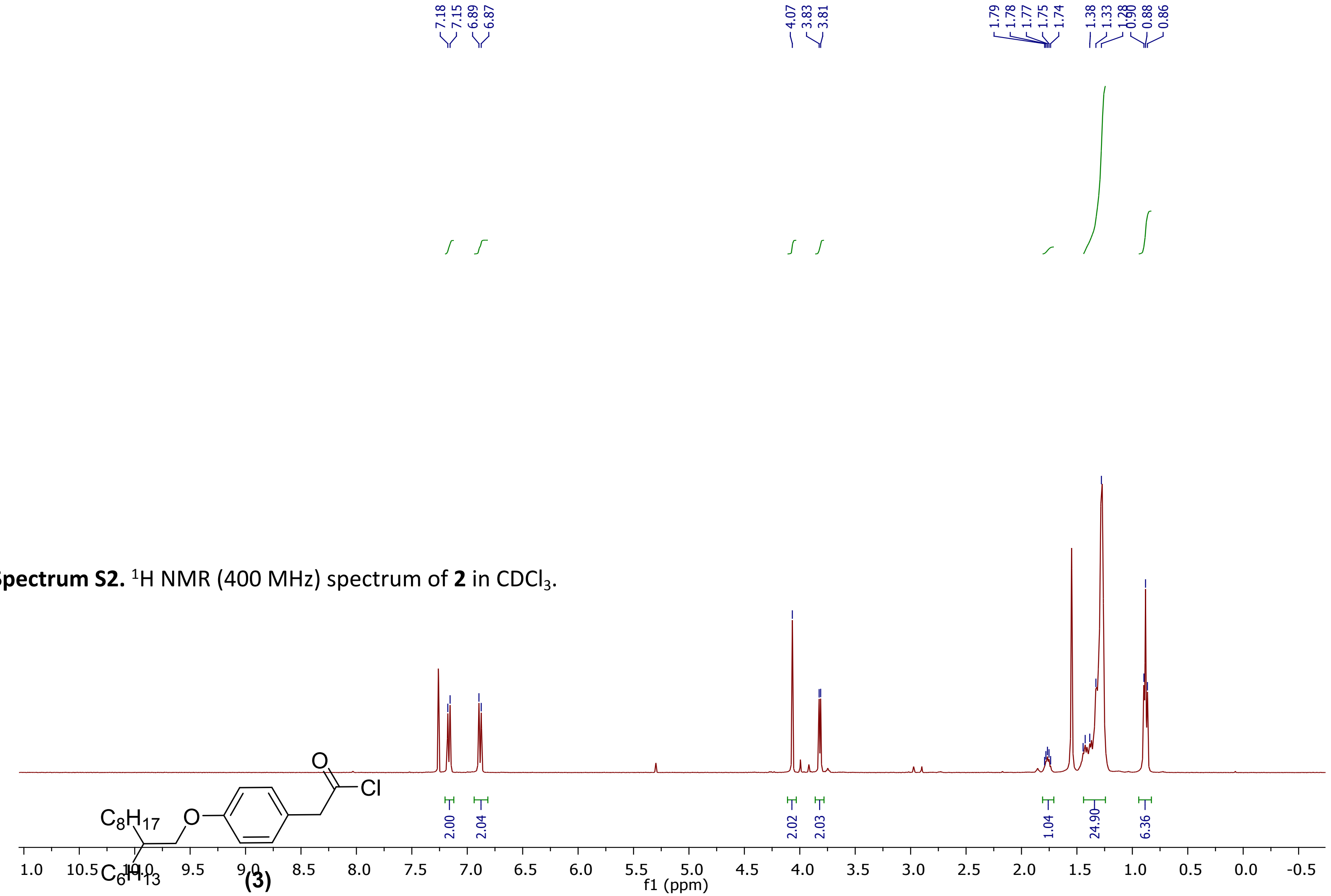

Spectrum S2. ^{1}H NMR (400 MHz) spectrum of **2** in $CDCl_3$.

(3)

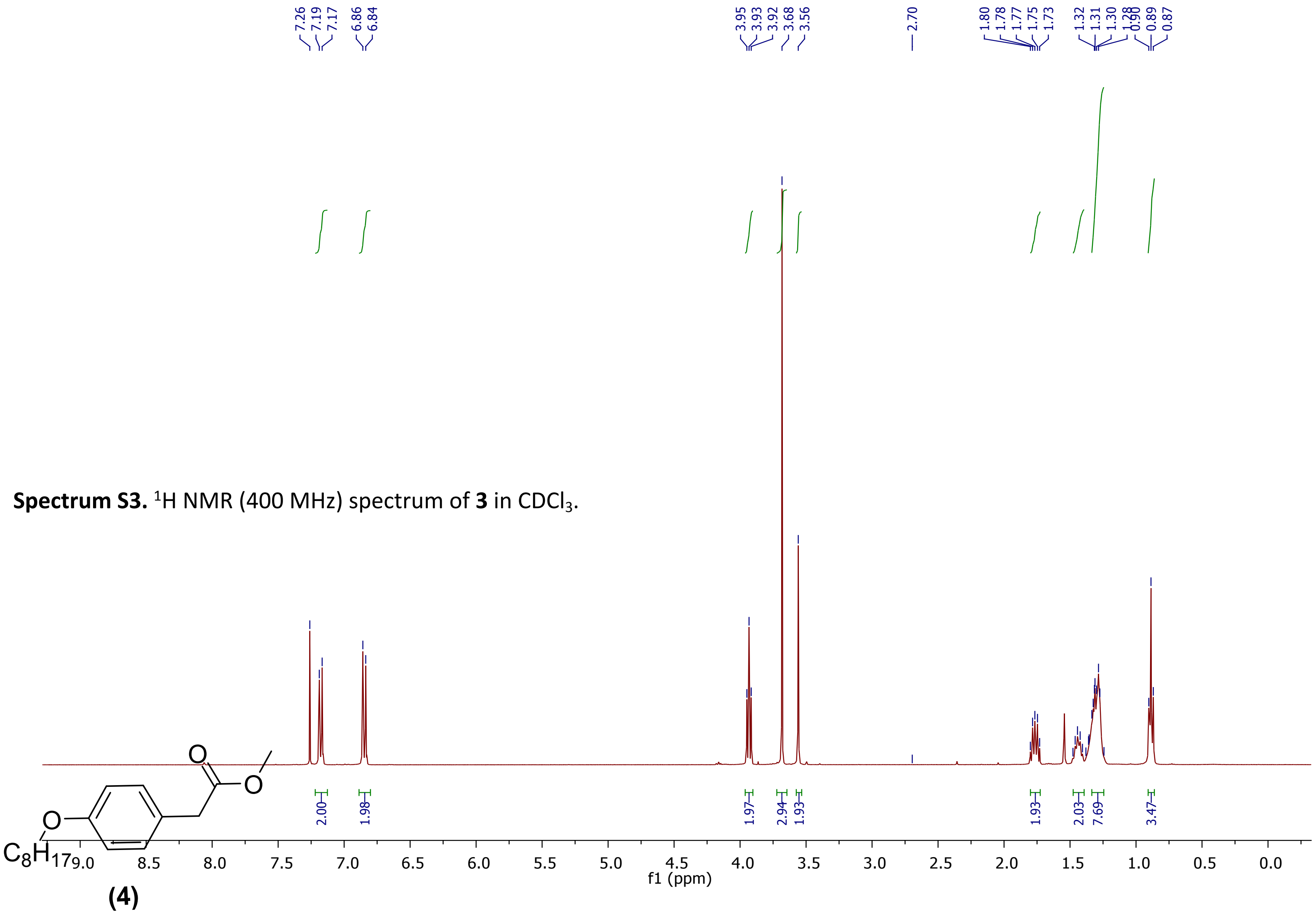

Spectrum S3. ^{1}H NMR (400 MHz) spectrum of **3** in $CDCl_3$.

(4)

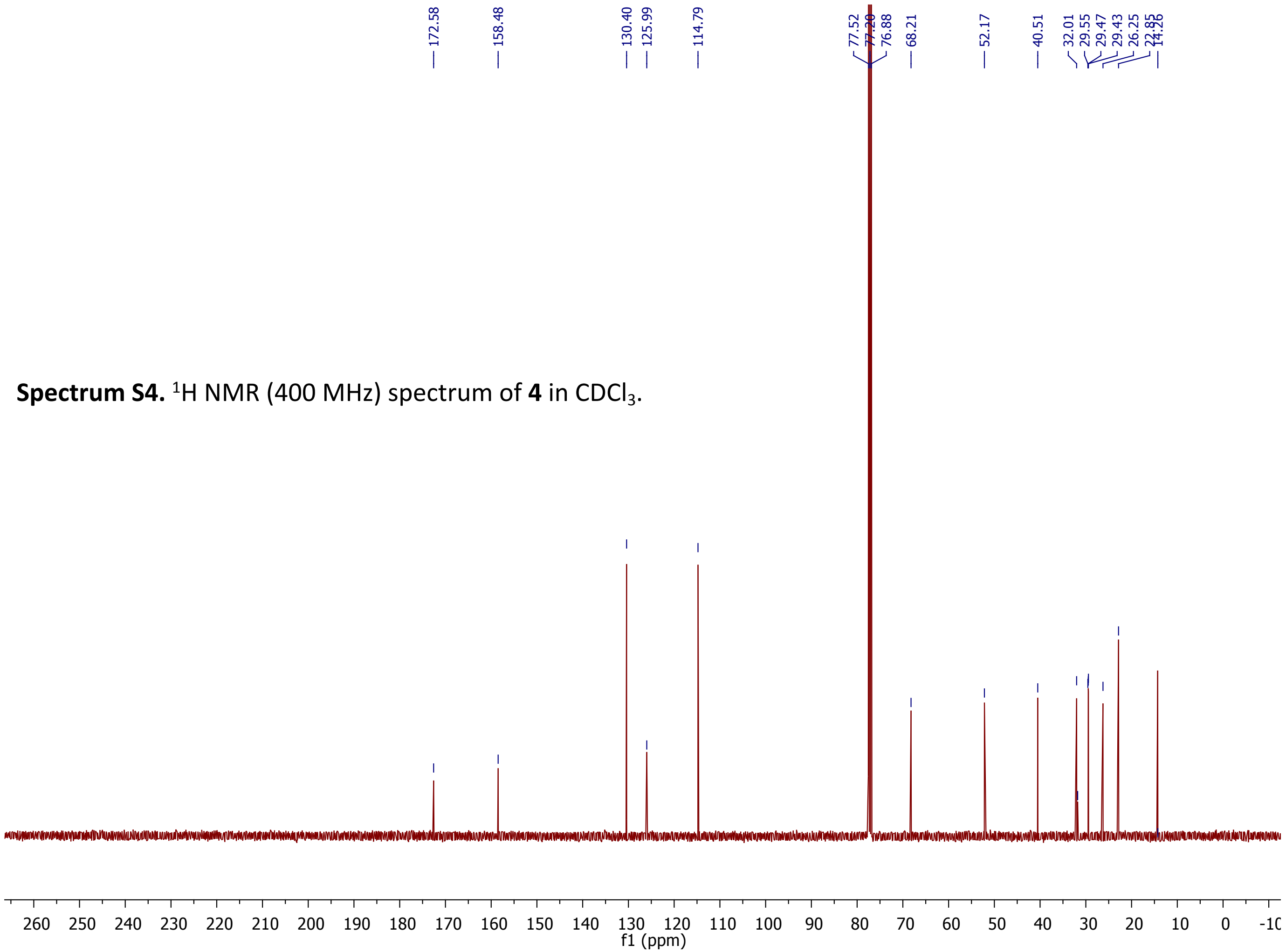

Spectrum S4. ^{1}H NMR (400 MHz) spectrum of **4** in $CDCl_3$.

Elemental Composition Report **Page 1**

Single Mass Analysis
Tolerance = 1000.0 PPM / DBE: min = -1.5, max = 50.0
Element prediction: Off
Number of isotope peaks used for i-FIT = 3

Monoisotopic Mass, Even Electron Ions
3 formula(e) evaluated with 1 results within limits (up to 5 closest results for each mass)
Elements Used:
C: 0-17 H: 0-27 O: 0-3
HAB_50550 M PURDY MP-OP ESP
HAB_50550 M PURDY MP-OP ESP 3047 (6.531) Cm (2870:3245)

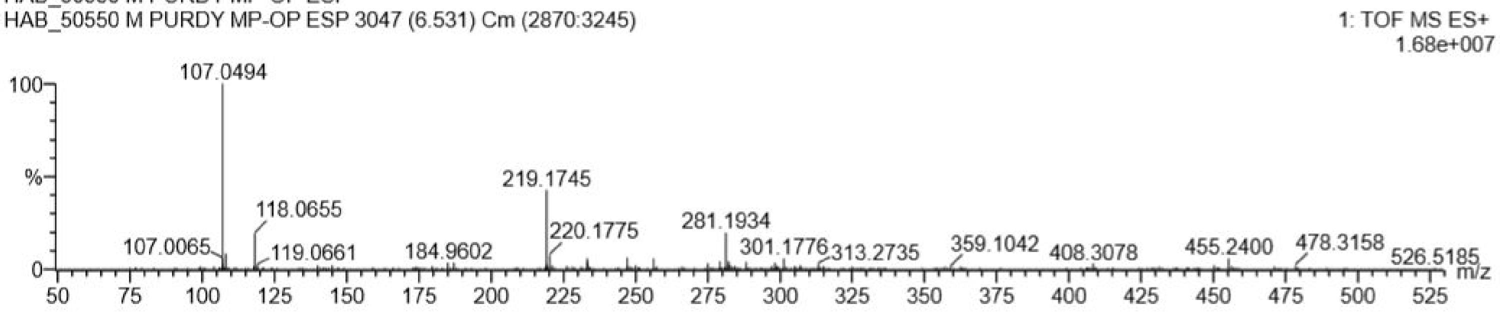

Minimum:				-1.5				
Maximum:		5.0	1000.0	50.0				
Mass	Calc. Mass	mDa	PPM	DBE	i-FIT	Norm	Conf(%)	Formula
279.1951	279.1960	-0.9	-3.2	4.5	1145.3	n/a	n/a	C17 H27 O3

Spectrum S6. High resolution mass spectrum of **4**.

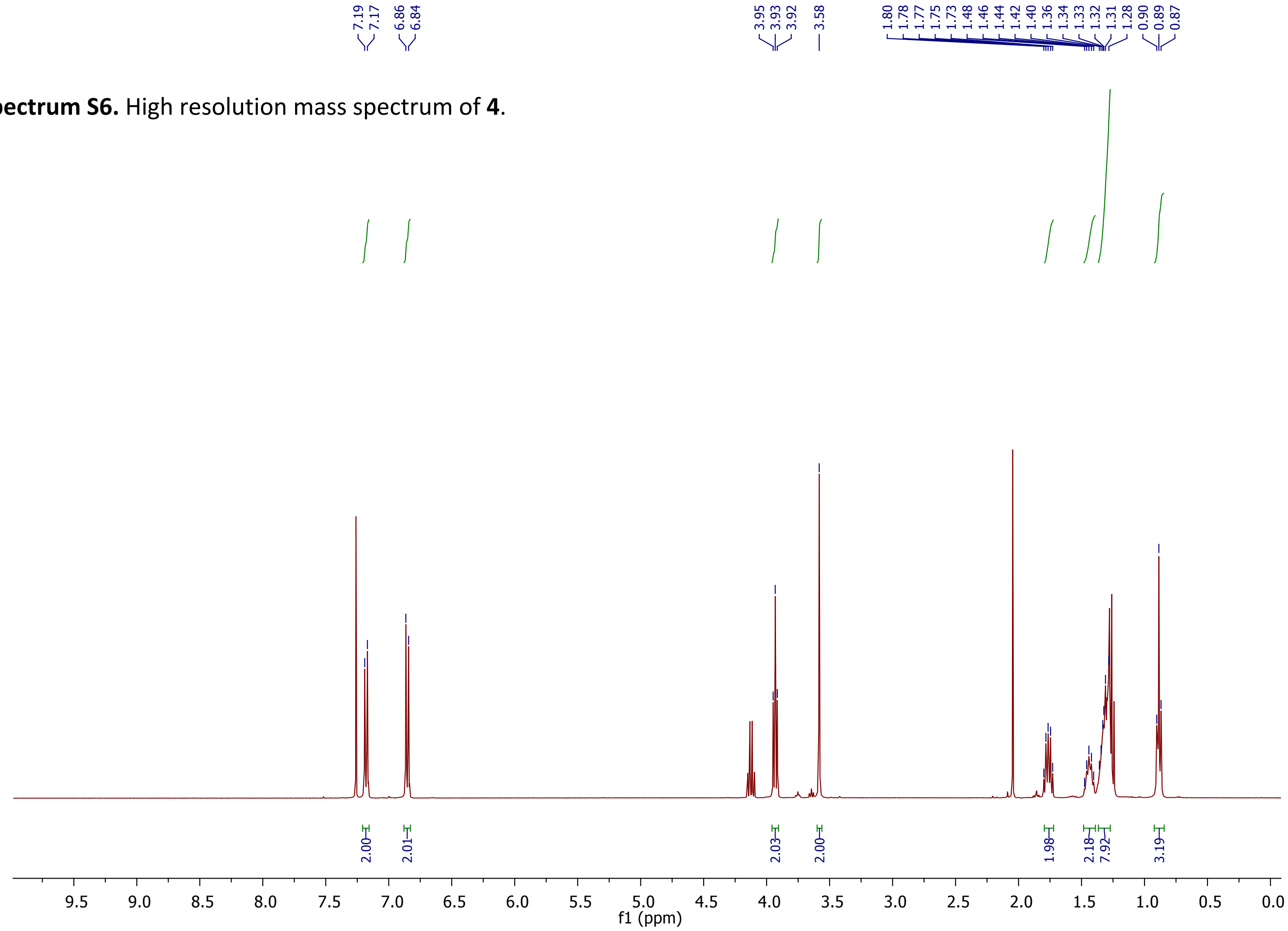

Spectrum S7. ^{1}H NMR (400 MHz) spectrum of **5** in $CDCl_3$.

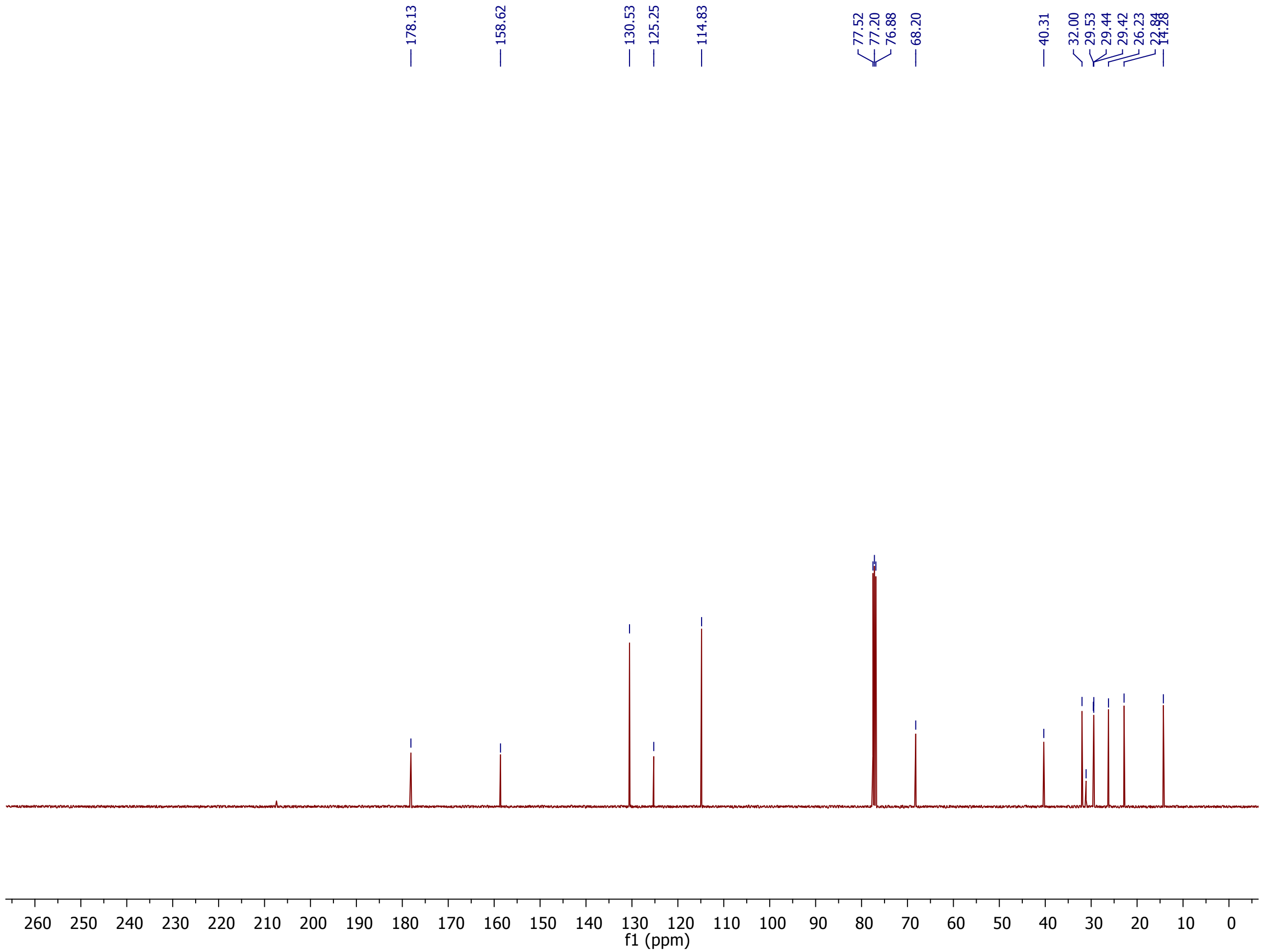

Spectrum S8. ^{13}C NMR (150 MHz) spectrum of **5** in $CDCl_3$.

Elemental Composition Report

Page 1

Single Mass Analysis

Tolerance = 1000.0 PPM / DBE: min = -1.5, max = 50.0
Element prediction: Off
Number of isotope peaks used for i-FIT = 3

Monoisotopic Mass, Even Electron Ions
3 formula(e) evaluated with 1 results within limits (up to 5 closest results for each mass)
Elements Used:
C: 0-17 H: 0-27 O: 0-3

HAB_50550 M PURDY MP-OP ESP
HAB_50550 M PURDY MP-OP ESP 3047 (6.531) Cm (2870:3245)

1: TOF MS ES+
1.68e+007

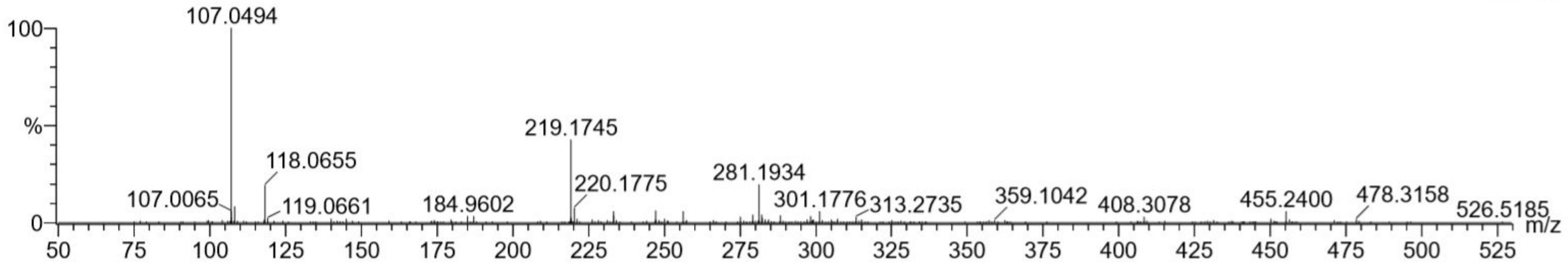

Minimum:				-1.5				
Maximum:		5.0	1000.0	50.0				
Mass	Calc. Mass	mDa	PPM	DBE	i-FIT	Norm	Conf(%)	Formula
279.1951	279.1960	-0.9	-3.2	4.5	1145.3	n/a	n/a	C17 H27 O3

Spectrum S9. High resolution mass spectrum of **5**.

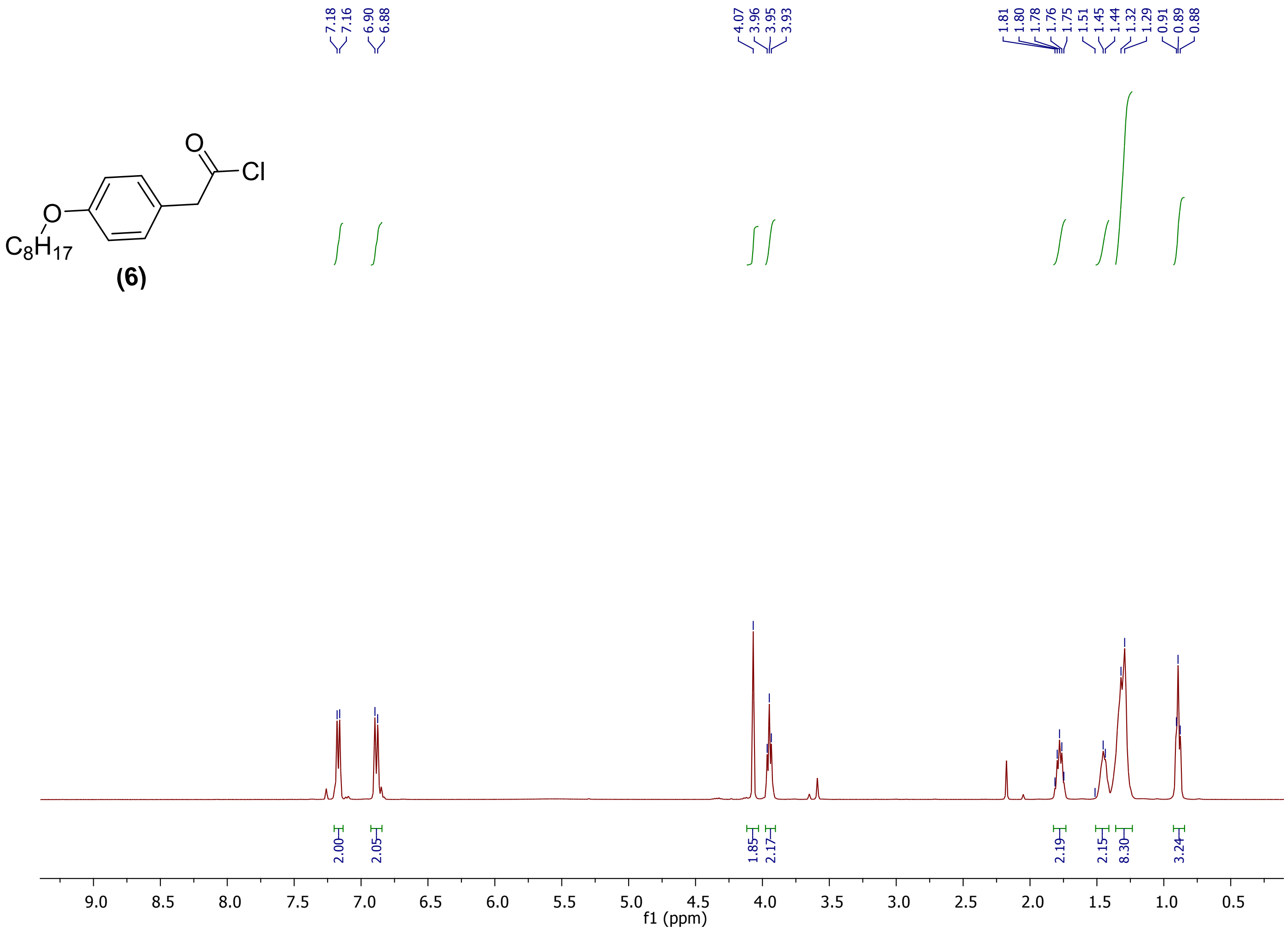

Spectrum S10. ^{1}H NMR (400 MHz) spectrum of **6** in $CDCl_3$.

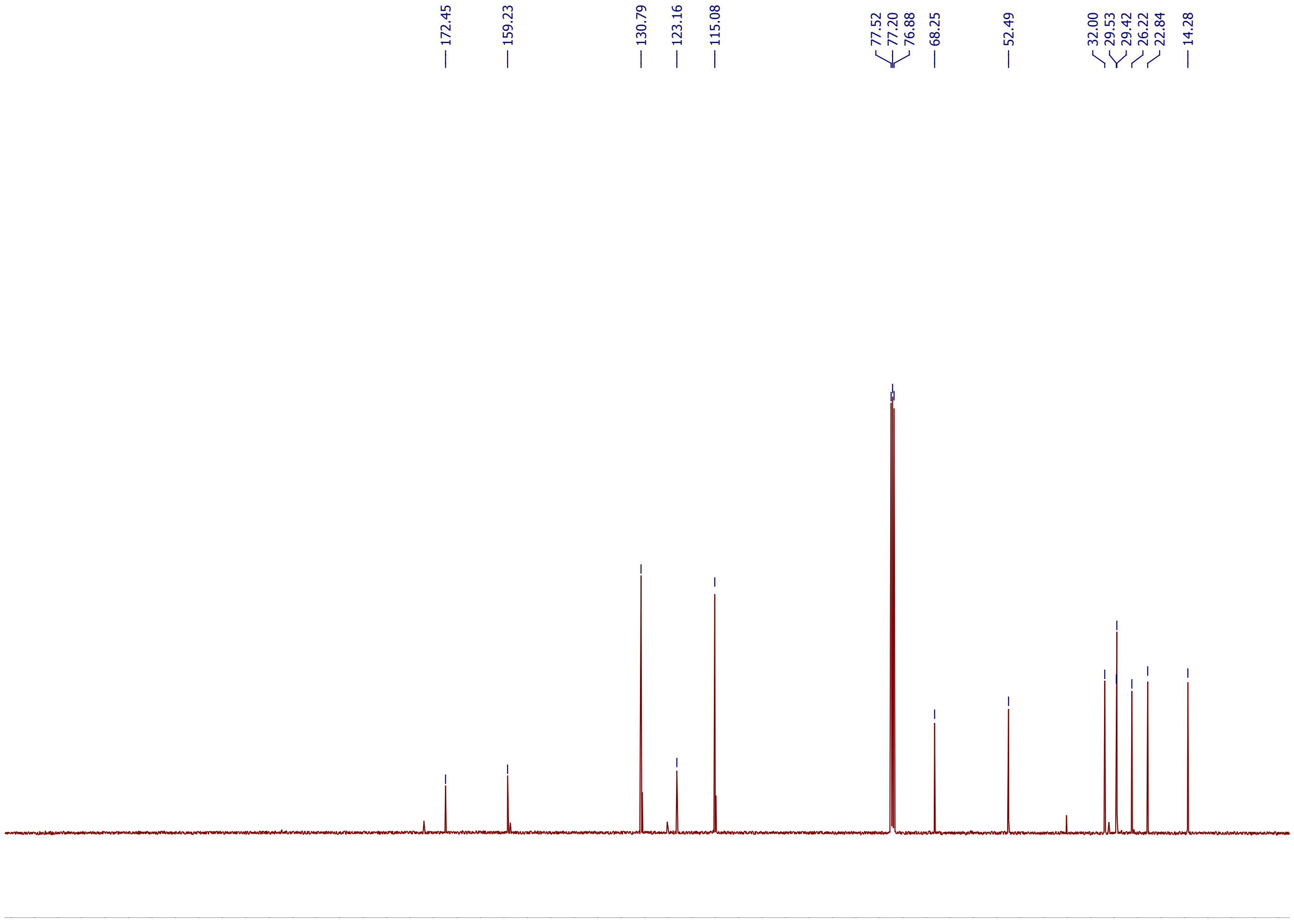

Spectrum S11. ^{13}C NMR (150 MHz) spectrum of **6** in $CDCl_3$.

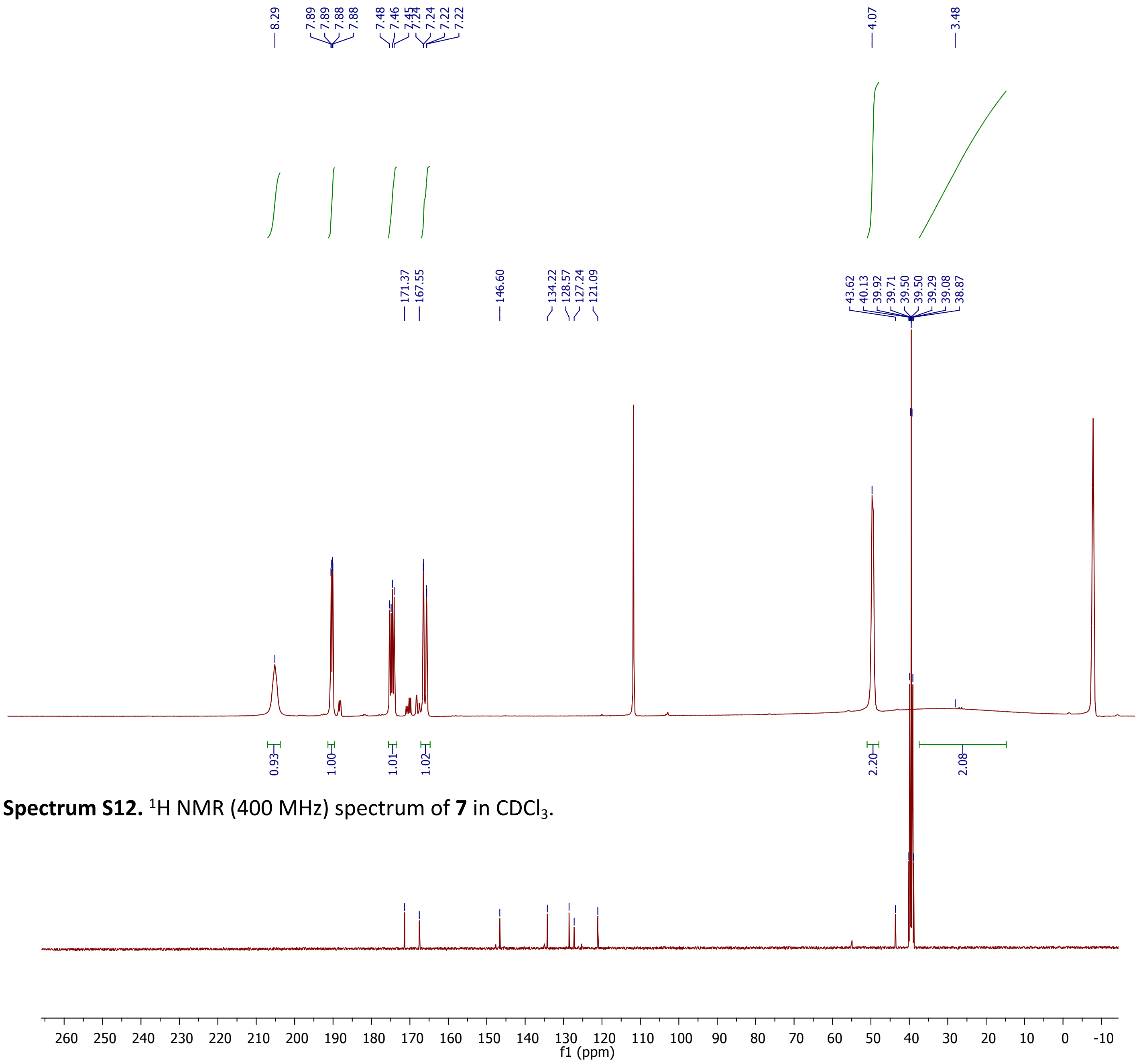

Spectrum S12. ^{1}H NMR (400 MHz) spectrum of **7** in $CDCl_3$.

Elemental Composition Report

Single Mass Analysis

Tolerance = 500.0 PPM / DBE: min = -1.5, max = 50.0
Element prediction: Off
Number of isotope peaks used for i-FIT = 3

Monoisotopic Mass, Even Electron Ions
13 formula(e) evaluated with 1 results within limits (up to 50 best isotopic matches for each mass)
Elements Used:
C: 0-8 H: 0-9 N: 0-2 O: 0-4
S1
HAB-MPurdy-50403-S1-2 1119 (2.425) 1: TOF MS ASAP+
5.67e+002

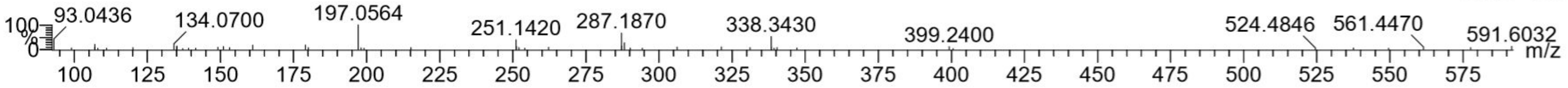

Minimum:				-1.5				
Maximum:		5.0	500.0	50.0				
Mass	Calc. Mass	mDa	PPM	DBE	i-FIT	Norm	Conf(%)	Formula
197.0564	197.0562	0.2	1.0	5.5	12.9	n/a	n/a	C8 H9 N2 O4

Spectrum S14. High resolution mass spectrum of **7**.

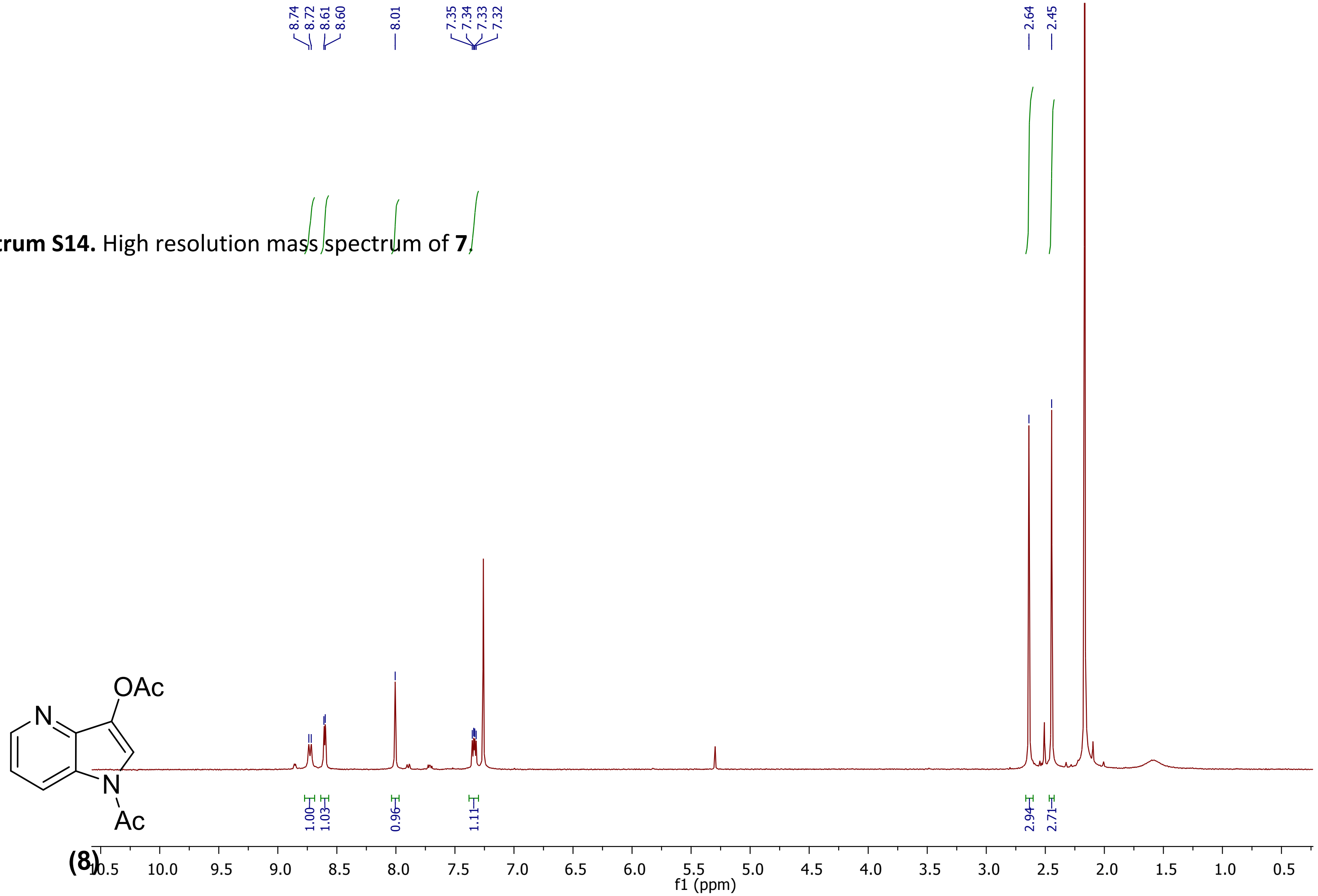

(8)

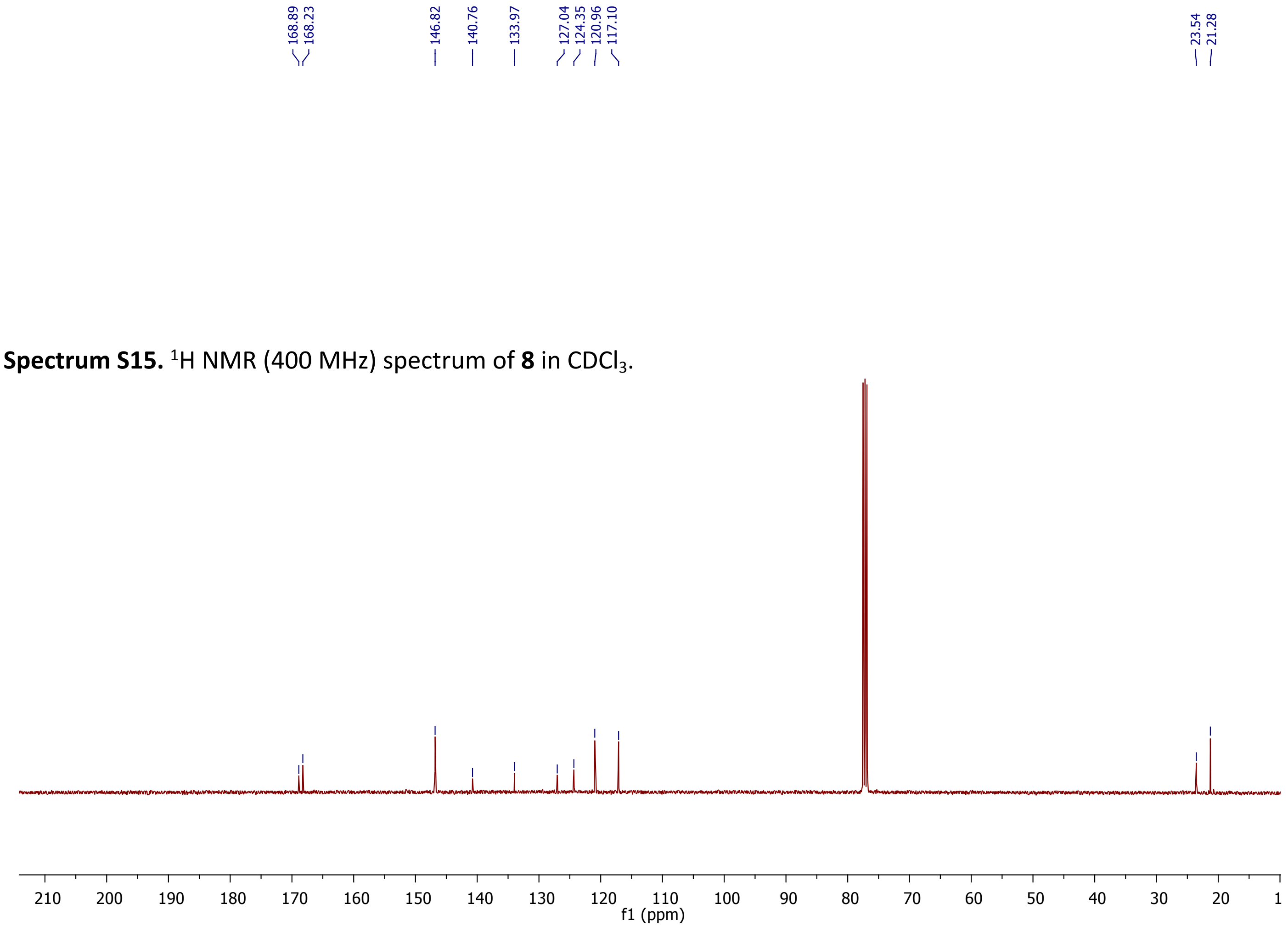

Spectrum S15. ^{1}H NMR (400 MHz) spectrum of **8** in $CDCl_3$.

Elemental Composition Report

Single Mass Analysis

Tolerance = 100.0 PPM / DBE: min = -1.5, max = 50.0
Element prediction: Off
Number of isotope peaks used for i-FIT = 3

Monoisotopic Mass, Even Electron Ions
7 formula(e) evaluated with 1 results within limits (up to 50 best isotopic matches for each mass)
Elements Used:
C: 0-11 H: 0-11 N: 0-2 O: 1-3

HAB_50454 M Purdy INDOL
HAB_50454 M Purdy INDOL 503 (1.093) Cm (420:515)

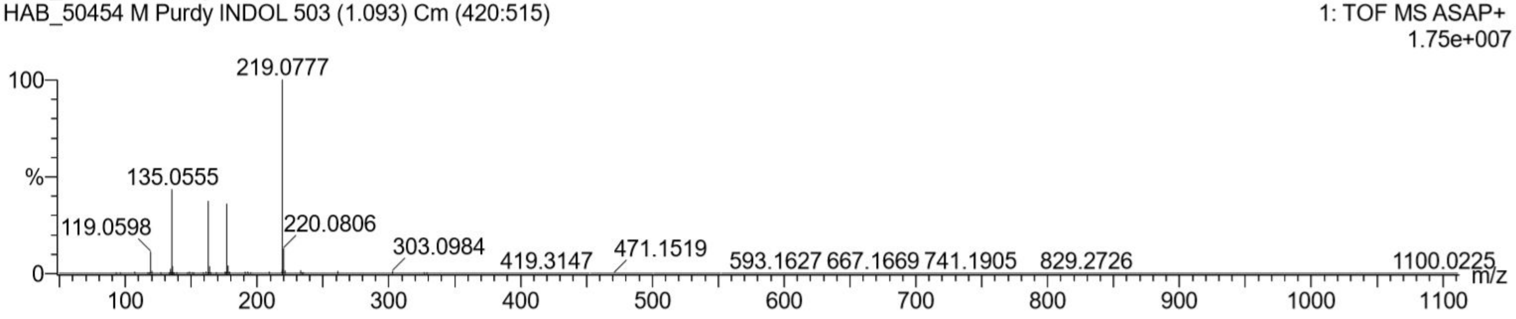

Minimum:				-1.5				
Maximum:		5.0	100.0	50.0				
Mass	Calc. Mass	mDa	PPM	DBE	i-FIT	Norm	Conf(%)	Formula
219.0777	219.0770	0.7	3.2	7.5	1989.5	n/a	n/a	C11 H11 N2 O3

Spectrum S17. High resolution mass spectrum of **8**.

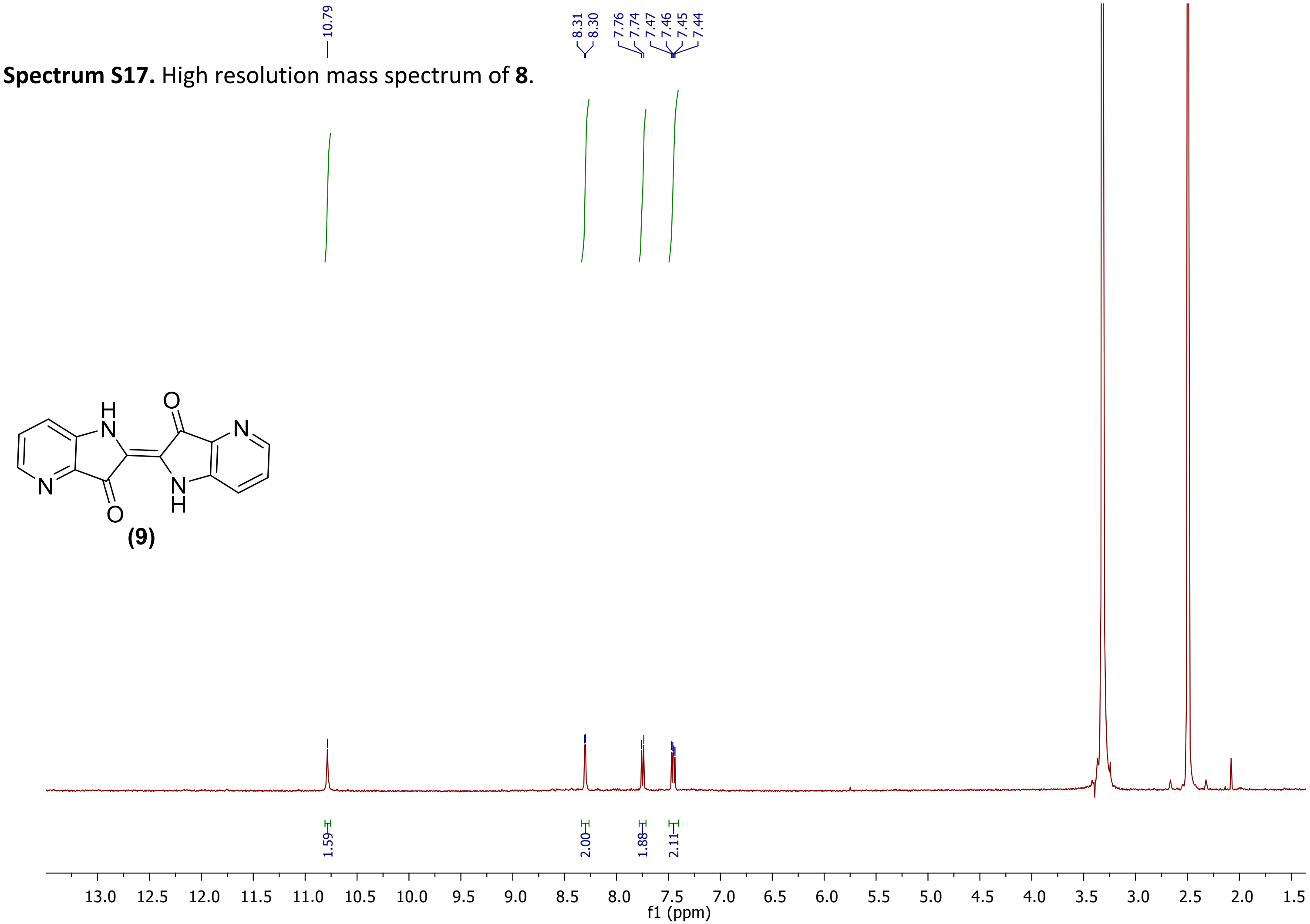

(9)

Spectrum S18. ^{1}H NMR (400 MHz) spectrum of **9** in DMSO-d_6.

Elemental Composition Report

Page 1

Single Mass Analysis

Tolerance = 500.0 PPM / DBE: min = -1.5, max = 50.0
Element prediction: Off
Number of isotope peaks used for i-FIT = 3

Monoisotopic Mass, Even Electron Ions
10 formula(e) evaluated with 1 results within limits (up to 50 best isotopic matches for each mass)
Elements Used:
C: 0-14 H: 0-9 N: 0-4 O: 0-2
S3
HAB-MPurdy-50403-S3-2 1742 (3.749)

1: TOF MS ASAP+
1.07e+003

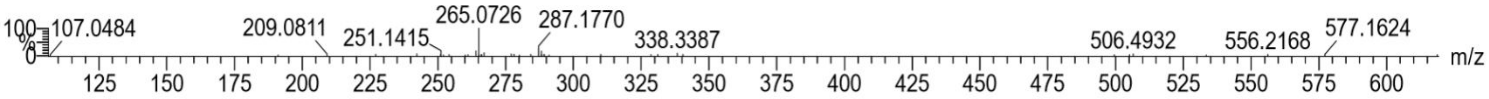

Minimum:				-1.5				
Maximum:		5.0	500.0	50.0				
Mass	Calc. Mass	mDa	PPM	DBE	i-FIT	Norm	Conf(%)	Formula
265.0726	265.0726	0.0	0.0	12.5	35.1	n/a	n/a	C14 H9 N4 O2

Spectrum S19. High resolution mass spectrum of **9**.

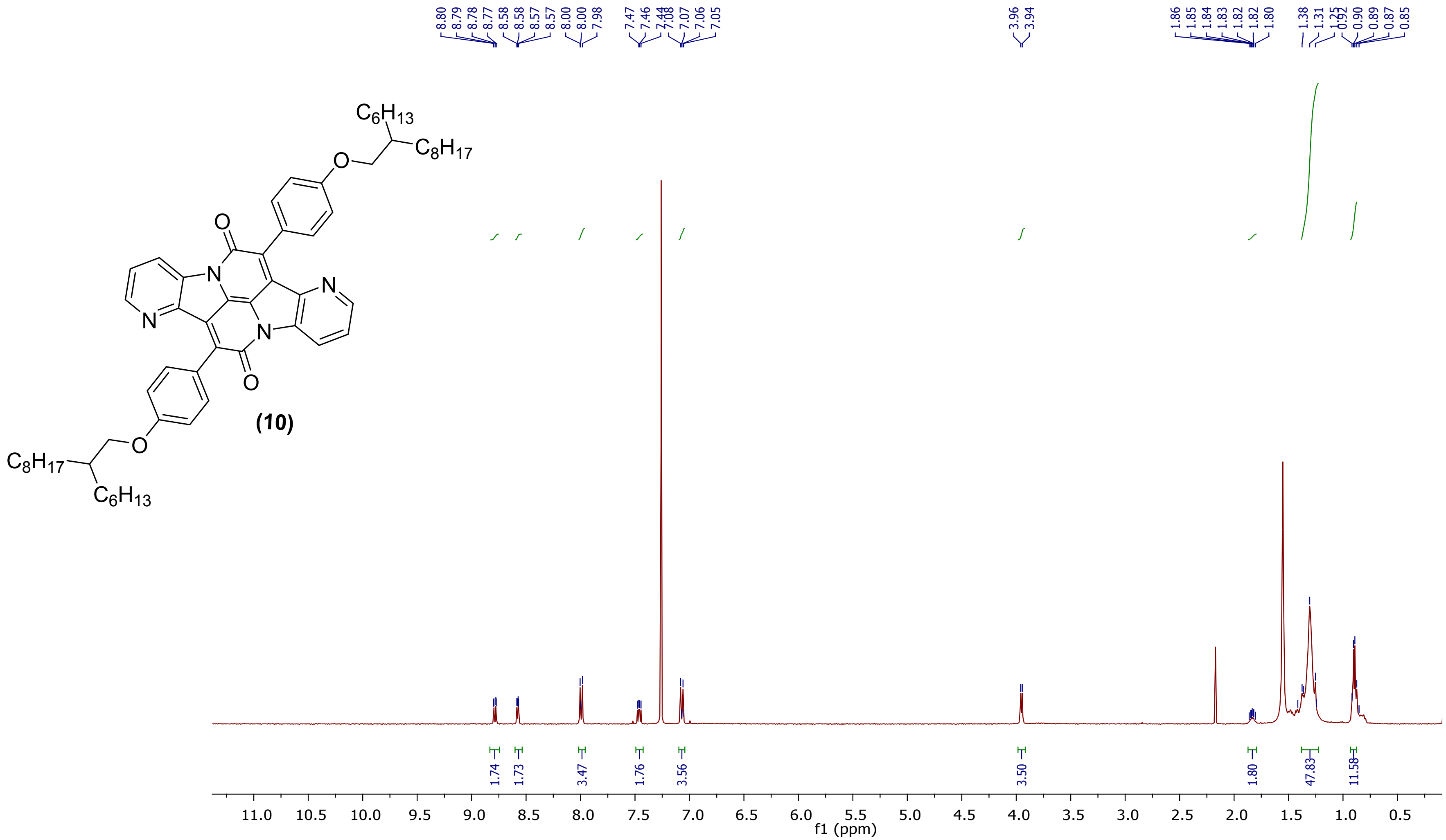

Spectrum S20. ^{1}H NMR (400 MHz) spectrum of **10** in $CDCl_3$.

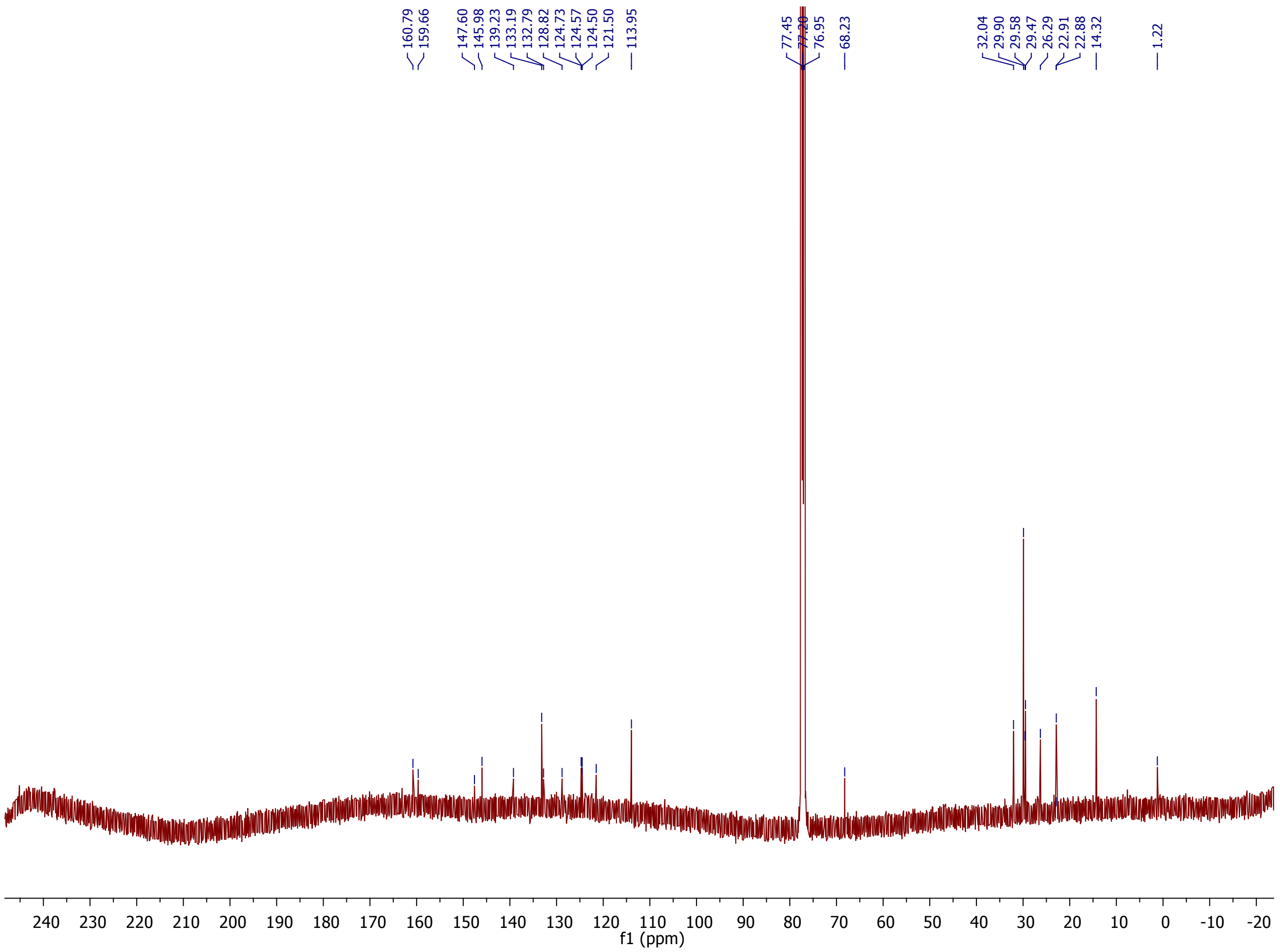

Spectrum S21. ^{13}C NMR (150 MHz) spectrum of **10** in $CDCl_3$.

Elemental Composition Report

Single Mass Analysis

Tolerance = 5.0 PPM / DBE: min = -1.5, max = 50.0
Element prediction: Off
Number of isotope peaks used for i-FIT = 3

Monoisotopic Mass, Even Electron Ions
263 formula(e) evaluated with 1 results within limits (all results (up to 1000) for each mass)
Elements Used:
C: 1-62 H: 1-81 N: 1-4 O: 0-4 I: 0-2

HAB_45589 M Purdy 2246 (4.827) Cm (2133:2285) 1: TOF MS ASAP+ 7.89e+003

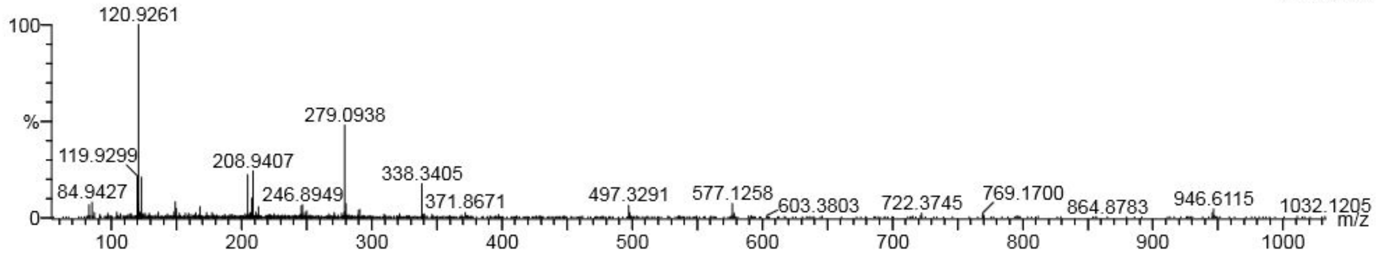

Minimum:				-1.5				
Maximum:		5.0	5.0	50.0				
Mass	Calc. Mass	mDa	PPM	DBE	i-FIT	Norm	Conf(%)	Formula
945.6266	945.6258	0.8	0.8	24.5	35.5	n/a	n/a	C62 H81 N4 O4

Spectrum S21. High resolution mass spectrum of **10**.

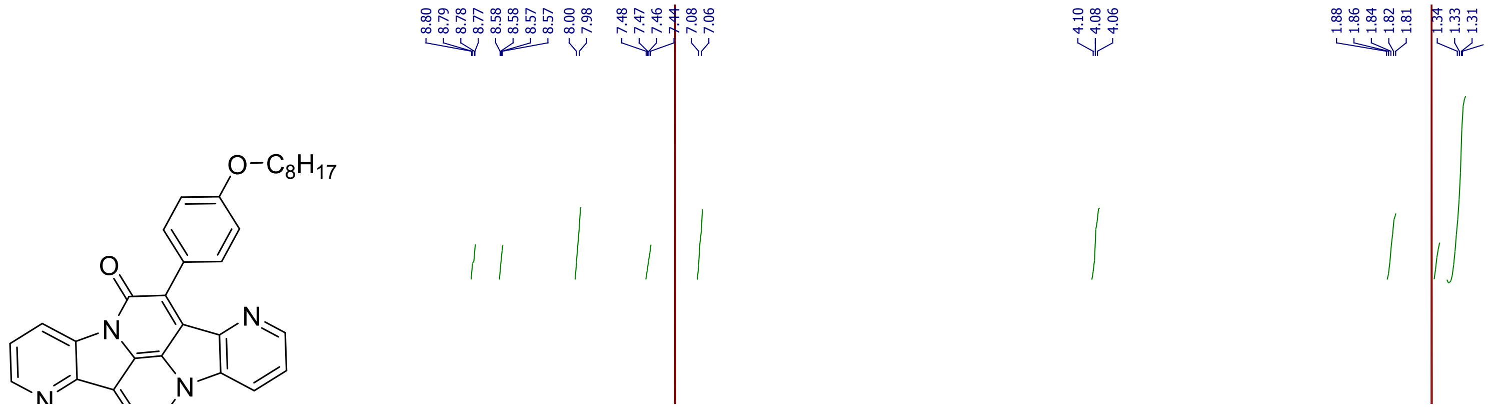

C_8H_1

Elemental Composition Report

Single Mass Analysis

Tolerance = 5.0 PPM / DBE: min = -1.5, max = 50.0
Element prediction: Off
Number of isotope peaks used for i-FIT = 3

Monoisotopic Mass, Even Electron Ions
171 formula(e) evaluated with 1 results within limits (all results (up to 1000) for each mass)
Elements Used:
C: 1-46 H: 1-49 N: 1-4 O: 0-4 I: 0-2

HAB_45587 M Purdy 2311 (4.951) Cm (2237:2312)

1: TOF MS ASAP+
7.00e+003

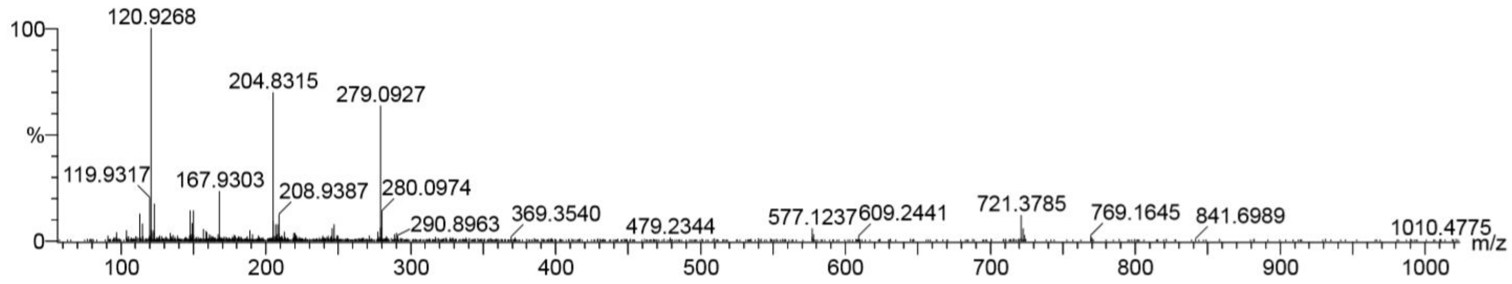

Minimum:				-1.5				
Maximum:		5.0	5.0	50.0				
Mass	Calc. Mass	mDa	PPM	DBE	i-FIT	Norm	Conf(%)	Formula
721.3785	721.3754	3.1	4.3	24.5	47.8	n/a	n/a	C46 H49 N4 O4

Spectrum S22. High resolution mass spectrum of **11**.

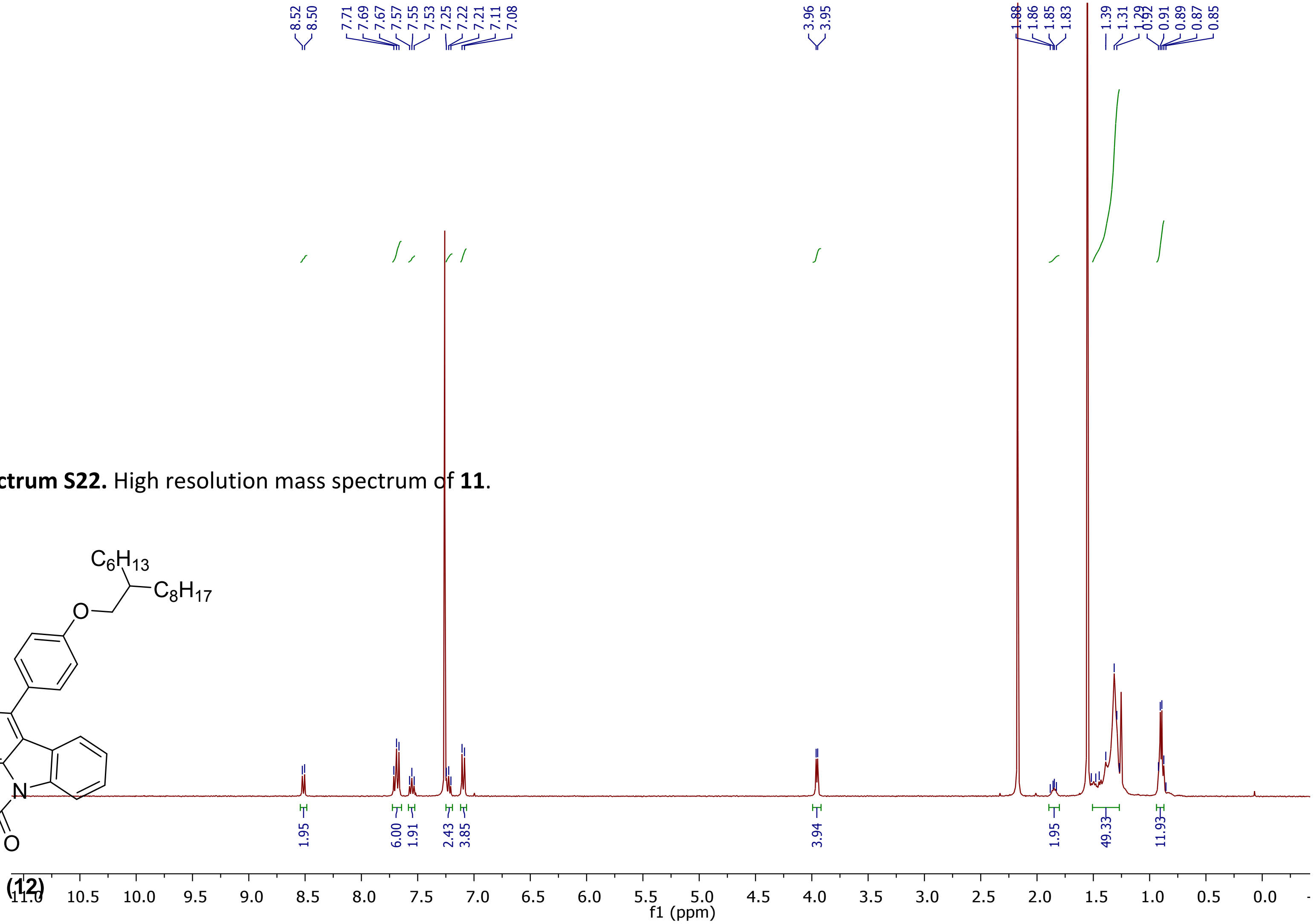

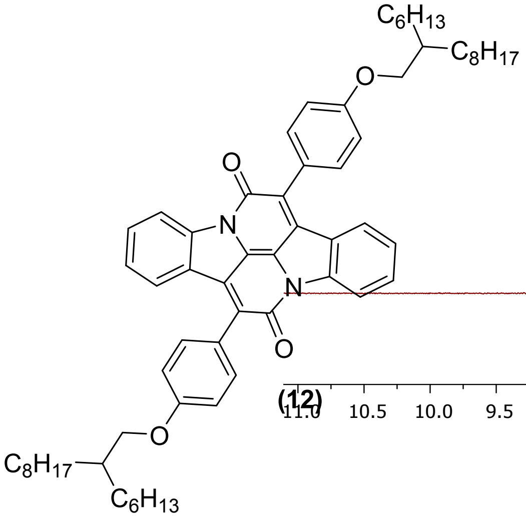

(12)

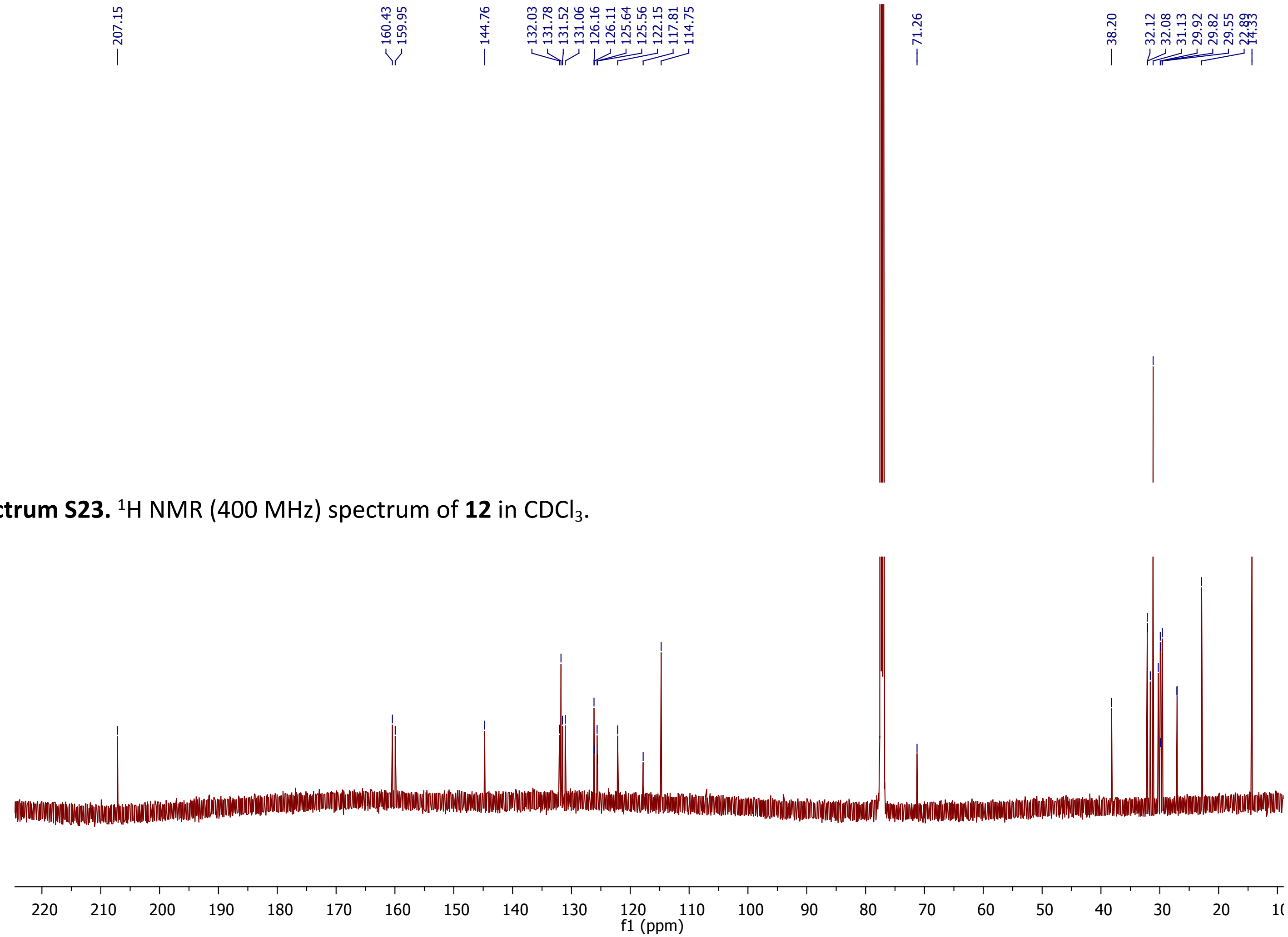

Spectrum S23. ^{1}H NMR (400 MHz) spectrum of **12** in $CDCl_3$.

Single Mass Analysis

Tolerance = 5.0 PPM / DBE: min = -1.5, max = 50.0
Element prediction: Off
Number of isotope peaks used for i-FIT = 3

Monoisotopic Mass, Even Electron Ions
140 formula(e) evaluated with 1 results within limits (all results (up to 1000) for each mass)
Elements Used:
C: 1-64 H: 1-83 N: 1-2 O: 0-4 I: 0-2

HAB_45590 M Purdy 2065 (4.443) Cm (2065:2066) 1: TOF MS ASAP+ 3.36e+002

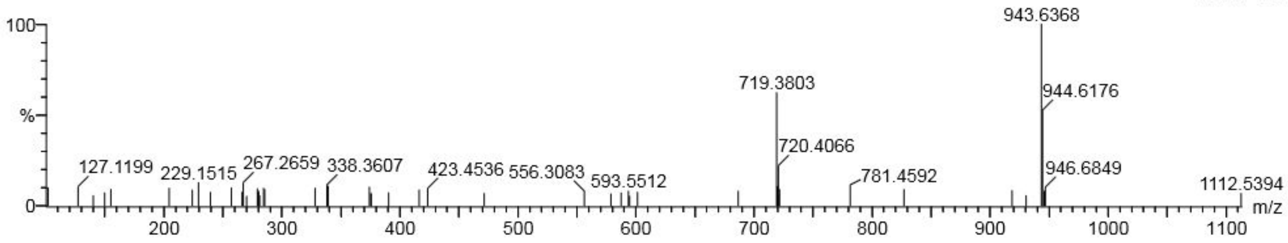

Minimum:				-1.5				
Maximum:		5.0	5.0	50.0				
Mass	Calc. Mass	mDa	PPM	DBE	i-FIT	Norm	Conf(%)	Formula
943.6368	943.6353	1.5	1.6	24.5	21.4	n/a	n/a	C64 H83 N2 O4

Spectrum S25. High resolution mass spectrum of **12**.

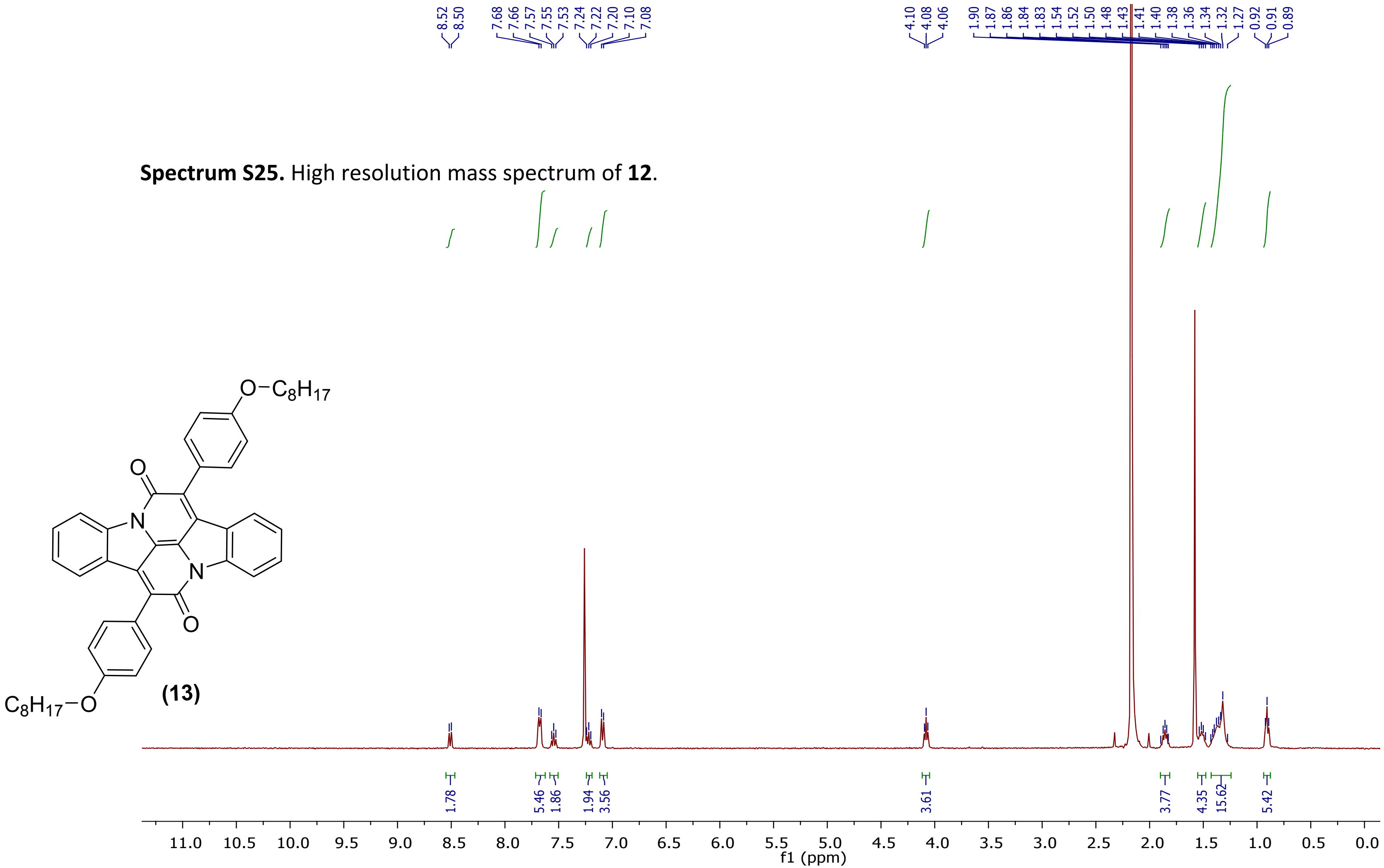

Spectrum S26. ^{1}H NMR (400 MHz) spectrum of **13** in $CDCl_3$.

Elemental Composition Report

Page 1

Single Mass Analysis

Tolerance = 50.0 PPM / DBE: min = -1.5, max = 50.0
Element prediction: Off
Number of isotope peaks used for i-FIT = 3

Monoisotopic Mass, Even Electron Ions
7 formula(e) evaluated with 1 results within limits (all results (up to 1000) for each mass)
Elements Used:
C: 0-48 H: 0-52 N: 0-2 O: 0-4

HAB_51193 M Purdy OC
HAB_51193 M Purdy OC 1895 (4.080)

1: TOF MS ASAP+
1.04e+005

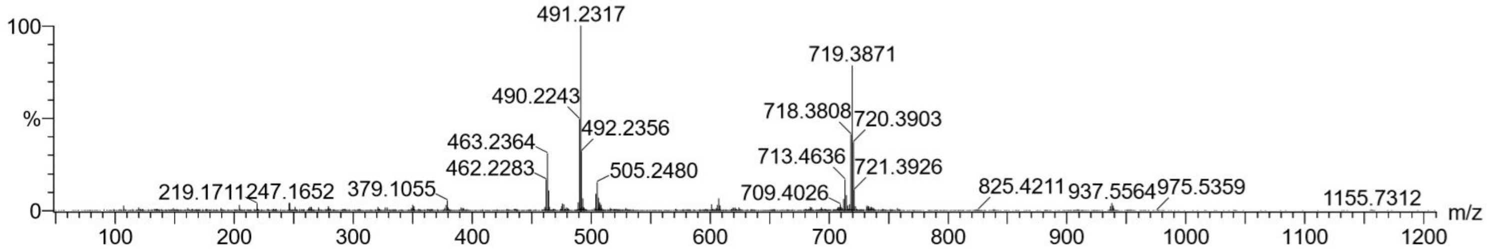

Minimum:				-1.5				
Maximum:		5.0	50.0	50.0				
Mass	Calc. Mass	mDa	PPM	DBE	i-FIT	Norm	Conf(%)	Formula
719.3871	719.3849	2.2	3.1	24.5	322.4	n/a	n/a	C48 H51 N2 O4

Spectrum S26. High resolution mass spectrum of **13**.

DFT

Molecule	Torsional Twist	Optical Band Gap (eV)	S_1 State Energy (eV)	S_1 State Torsion	T_1 State Energy (eV)	T_1 State Torsion	T_1: S_1 ratio	η_{Ph} (%)
OHC	41.6 °[a], 45.1 °[b]	2.04[c]	2.30[d]	37.2 °[h]	1.24[d]	33.0 °[i]	0.53[e]	12[f]
OHAC	38.5 °[b]	1.91[c]	2.13[d]	31.9 °[h]	1.07[d]	30.1 °[i]	0.5[e]	18[f]
OHAC	45.1 °[g]		2.14[d]	31.9 °[h]	1.08[d]	30.1 °[i]	0.5[e]	

Table S1. Data extracted from theoretical calculations performed on OHC and OHAC. [a]Torsional twist take from single crystal structure. [b]Torsional Twist taken from DFT optimized structures. [c]Optical band gaps estimated from on-set of UV-Vis absorption spectra. [d]Energies of the first singlet (S_1) and first triplet (T_1) excited states. [e]Ratio of excited state energies. [f]Spin population density of the delocalised diradical. on the outer phenoxy groups. [g]Arbitrarily set torsion.[h]Torsional twist of S_1 state. [i]Torsional twist of T_1 state.

Single crystal X-ray diffraction measurements

Single crystals of OC were obtained through slow solvent evaporation form a diluted acetone/*p*-xylene solvent mixture at ambient conditions.

The diffraction data for OC was collected on a four-circle *Agilent SuperNova* (Dual Source) single crystal X-ray diffractometer using a micro-focus CuK_α X-ray beam (λ = 1.54184 Å) and an *Atlas* CCD detector. The sample temperatures were controlled with an *Oxford Instruments* cryojet. The data was processed using the *CrysAlis^{Pro}* programme package from *Rigaku Oxford Diffraction*.[2] The crystal structures were solved with the *SHELXT* programme,[3] used within the *Olex2* software suite,[4] and refined by least squares on the basis of F^2 with the *SHELXL*[5] programme using the *ShelXle* graphical user interface.[6] All non-hydrogen atoms were refined anisotropically by the full-matrix least-squares method. Hydrogen atoms associated with carbon atoms were refined isotropically [U_{iso}(H) = 1.2U_{eq}(C)] in geometrically constrained positions. The asymmetric unit of OC is shown in Figure S1, while the crystallographic and refinement parameters are listed in Table S1.

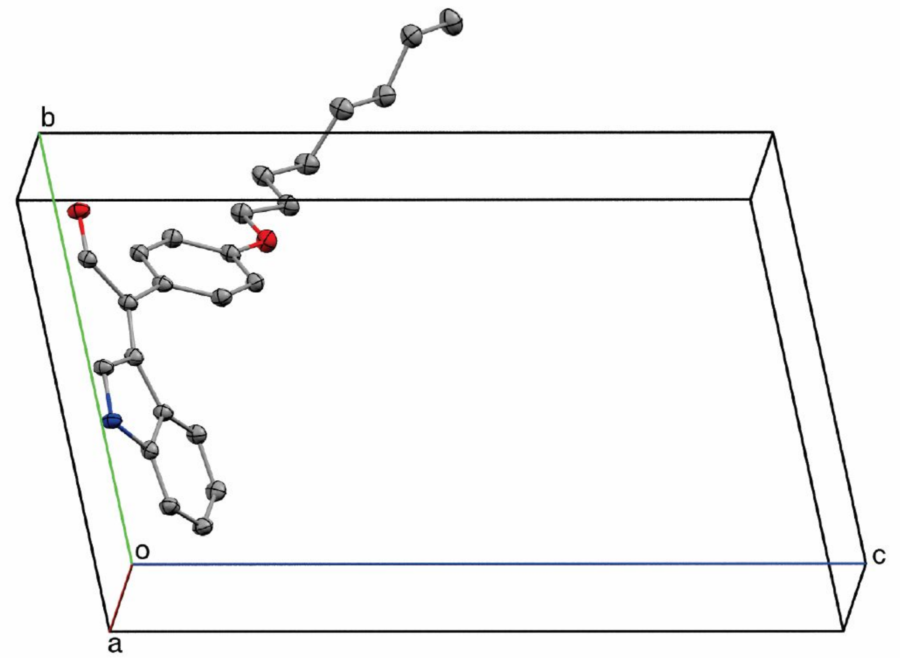

Figure S1. The asymmetric unit of compound OC. The thermal ellipsoids are drawn at the 50% probability level, while hydrogen atoms are omitted for clarity. Colour scheme: carbon – grey, nitrogen – blue, oxygen – red.

	OC
empirical formula	$C_{48}H_{50}N_2O_4$
M_r / g mol^{-1}	718.90
crystal system	triclinic
space group	$P\overline{1}$
a / Å	5.2780(3)
b / Å	10.3858(8)
c / Å	17.0622(12)
α / °	100.743(6)
β / °	90.072(5)
γ / °	95.237(6)
V / Å^3	914.90(11)
Z	1
ρ_{calc} / g cm^{-3}	1.305
T / K	150.0(1)
μ / mm^{-1}	0.647
F(000)	384
crystal size / mm^3	0.41 × 0.04 × 0.03
radiation	CuK_α (λ = 1.54184 Å)
2θ range for data collection / °	4.352−73.814
index ranges	$-4 \leq h \leq 6$<br>$-12 \leq k \leq 12$<br>$-20 \leq l \leq 21$
number of collected reflections	5859
unique reflections	3510
number of unique reflections	2956 [$I > 2\sigma(I)$]
R_{int}	0.0222
$R(F)$, $F > 2\sigma$(F)	0.0399
$wR(F^2)$, $F > 2\sigma$(F)	0.0988
$R(F)$, all data	0.0519
$wR(F^2)$, all data	0.01051
Δ_r (max., min.) e Å^{-3}	0.268/−0.236
CCDC deposition number	2164360

Table S2. Crystallographic and refinement parameters of OC.

OAC Crystallisation experiments

The different crystallisation experiments used for aza-cibalackrot (OAC) single crystal growth are detailed below:

1. Solution crystallisation and slow solvent diffusion

- Chlorinated solvents worked best: chloroform, dichloromethane, dichloroethane, tricholoroethylene; the precipitation rate was controlled through the addition of a small amounts of methanol, ethanol, butanol, EtOAc, DMSO, dioxane, hexane MEK, toluene (a range of solvent compositions was used in the crystallisation experiments).
- Very small and thin plates were obtained during experiments using solvent diffusion and from the crysallisation using solvent mixtures; these single crystals were too thin for data collection (the bigger once gave diffused, smeared peaks – most likely because the crystals were bent).
- Crystallisation from chloroform yielded nicely shaped prisms, but too small for data collection on a laboratory diffractometer.

2. Interfacial crystallisation

- Chloroform/water (no single crystals).

3. Melt crystallisation

- Melting point and the decomposition temperature are too close to obtain single crystals.

S5 GIWAXS

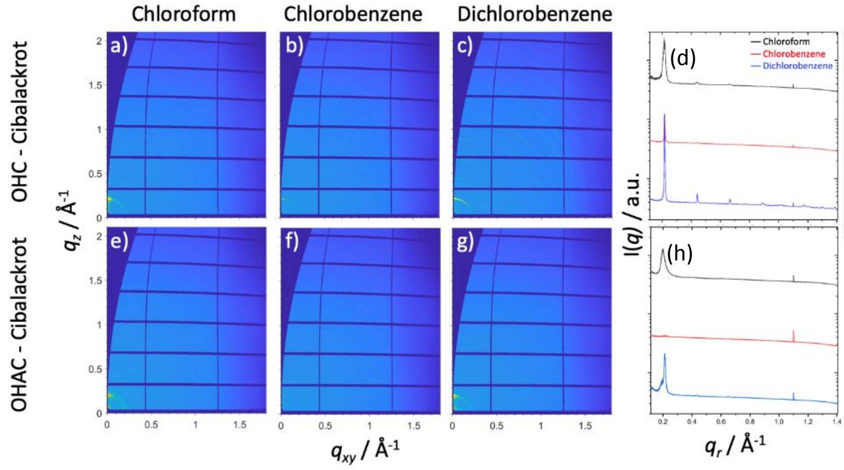

(i)	Casting solvent	1st order peak position / Å^{-1}	d-spacing / Å
OH - Cibalackrot	Chloroform	0.21475	29.3
	Chlorobenzene	0.21675	29.0
	Dichlorobenzene	0.21675	29.0
OHAC - Cibalackrot	Chloroform	0.20132	31.2
	Chlorobenzene	NA	NA
	Dichlorobenzene	0.21162	29.7

Figure S2: Two-dimension grazing incidence scattering data for thin films of (a) OHC drop-cast from chloroform (b) OHC drop-cast from chlorobenzene (c) where OHC drop-cast from dichlorobenzene (d) OHC radial integrated data (e) OHAC drop-cast from chloroform (f) OHAC drop-cast from chlorobenzene (g) where OHAC drop-cast from dichlorobenzene (h) OHAC radial integrated data (i) d-spacing of OHAC and OHC thin films

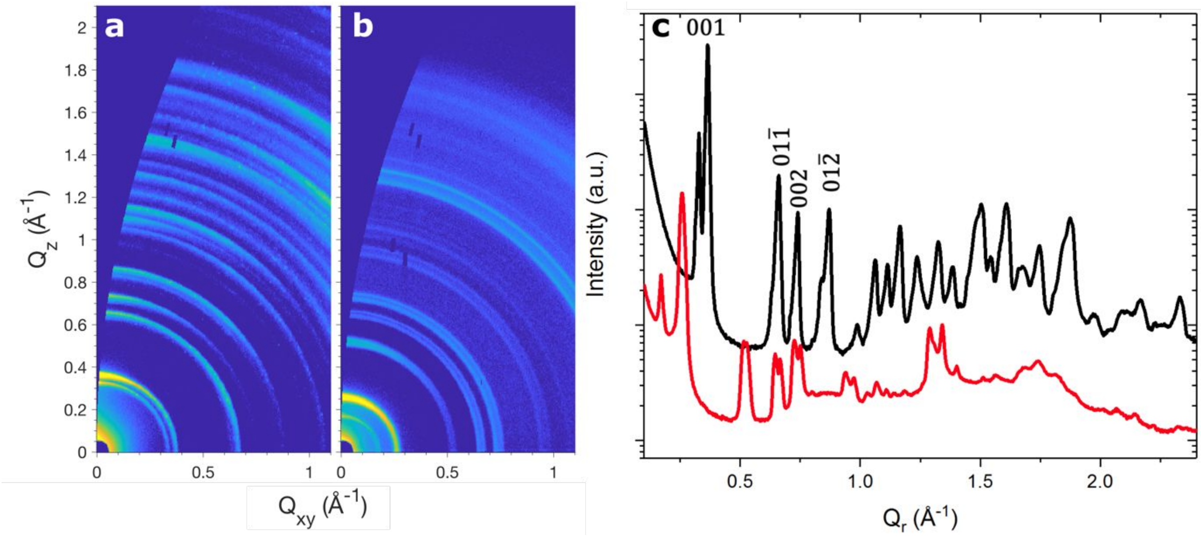

Figure S3: Two-dimension grazing incidence scattering data for (a) OC and (b) OAC, with corresponding radial integrated data (c) where OC (black line) OAC (red line).

Grazing incidence wide angle X-ray scattering (GIWAXS) was employed to gain further insight into the microstructure of drop-cast OC and OAC films. The GIWAXS data presented in Figure S2, comprises numerous Debye-Scherrer rings and indicates that both OC and OAC films are highly crystalline with many large, randomly orientated crystal grains. For the OC film the crystal structure observed via GIWAXS is consistent with the single crystal X-ray diffraction studies (with the 001, $01\bar{1}$, 002 and $01\bar{2}$ reflections labelled in Figure S2**c** for clarity). An

additional, low Q peak at 0.33 $Å^{-1}$ is observed for the OC film, which most likely arises from the larger scale lamellae packing. Whilst we have not been able to perform single crystal X-ray diffraction of OAC the GIWAXS data indicates that the packing between OC and OAC is largely similar, albeit with the scattering peaks shifting to lower Q. Interestingly, we find that through manipulating the unit cell parameters of the OC crystal structure it was possible to replicate the observed shifts of the 001, $01\bar{1}$, 002 and $01\bar{2}$ reflections for OAC (using CrystalDiffract v6.8.5 software). The unit cell dimensions obtained via single crystal X-ray diffraction of OC and estimated from GIWAXS data of OAC are shown in Table S2 and indicate that the modification of the OAC has a considerable impact on crystal packing, with a large increase in the b and c unit cell dimensions. We speculate that the reducing the of torsion of the peripheral phenoxy rings alters how the octyl hexyl groups are arranged in the unit cell. Further, for the OAC the low Q feature ascribed to lamellae packing is further shifted to 0.17 $Å^{-1}$. As both OC and OAC possess octyl functional groups, we hypothesize that the large shift in the low Q lamellae peak between OC and OAC indicates a change from the octyl groups being interdigitated for OC and non-interdigitated for the OAC.

	**OC unit cell parameters** (obtained via single crystal X-ray diffraction)	**OAC unit cell parameters** (estimated from GIWAXS data)
a (Å)	5.3	5.3
b (Å)	10.4	13.4
c (Å)	17.1	26.0

Table S3 Unit cell parameters of OC and OAC.

Method

Grazing Incidence Small Angle X-ray Scattering. Grazing incidence wide-angle X-ray scattering (GIWAXS) was performed on the Xuess 2.0 instrument equipped with an Excillum MetalJet liquid gallium X-ray source. Alignment was performed on silicon substrates via three iterative height (z) and rocking curve (Ω) scans, with the final grazing incidence angle set to $\Omega = 0.15°$. Scattering patterns were recorded on a vertically-offset Pilatus 1M detector with a sample to detector distance of 280 mm, calibrated using a silver behenate standard to achieve a q-range of 0.05 – 2.3 $Å^{-1}$. Two-dimensional images were recorded with exposure times of 600 s. Data correction and reduction was performed using Xenocs XSACT software, with two-dimensional scattering data reshaped and reduced to one-dimension via radial integration, which was performed with a mask to remove contributions from “hot pixels”, the substrate horizon and the reflected beam.

S6 Optical Spectroscopy Methods

All samples were prepared in an N_2 atmosphere glovebox. Stock solutions of octyl-hexyl-cibalackrot (OHC) and octyl-hexyl-aza-cibalackrot (OHAC) were made with chlorobenzene at 10 mg ml^{-1} and 2.5 mg ml^{-1} respectively. Solution measurements were performed using quartz cuvettes with a 1 mm path length, sealed with a PTFE cap, PTFE tape and parafilm. Solutions for transient absorption spectroscopy were encapsulated in a small volume microcuvette with a 200 μm path length, sealed with a UV-cure epoxy resin

Thin-films were prepared by drop casting onto fused silica substrates, washed with acetone and iso-propyl alcohol. Thin-films were encapsulated by a glass cover slip and sealed with a two-part rapid cure epoxy resin.

Steady state absorption measurements were performed using a Shimadzu UV3600 Plus UV-Visible spectrometer with reference beam. Steady state photoluminescence (PL) measurements and PL quantum yield (PLQE) were performed inside an integrating sphere using adapted methods as described by de Mello *et al.*[2], with a 520 nm continuous excitation source and an Andor iDus DU420A BVF Si detector. Measurements were taken at room temperature on encapsulated films. PLQE calculations were performed as described by de Mello *et al.*[2]

Time-resolved PL decay with time correlated single photon counting (TCSPC) measurements were performed using an Edinburgh Instruments FLS1000-DD-stm Steady State, Fluorescence Lifetime and Phosphorescence Lifetime Spectrometer. Excitation and emission spectra of the triplet sensitization measurements were also performed on this apparatus.

Ultrafast transient absorption (TA) spectra were measured using apparatus previously reported[3]. Time delays of 100 fs to 2ns were generated with a delay stage. The probe (Light Conversion Pharos 40kHz, 260fs, 1030nm) is modified using tunable, broadband, white-light generation, spanning 575-950 nm. The pump is created by passing the Pharos 1030nm output to an Optical Parametric Amplifier (LYRA) to convert the fundamental to any tunable wavelength. Within this study, the pump was centered at 550 nm, or at 650 nm for the triplet sensitization measurements. The signal was recorded on a JAI silicon detector using a single beam (no reference beam) geometry.

Triplet sensitisation measurements were recorded at longer times (1 ns – 3 μs) using a nanosecond transient absorption apparatus. Time delays were generated with an electronic delay generator (Stanford Research Systems DG645). The probed (LEUKOS Disco 1) spanned 575-800 nm. The pump (Innolas Picolo 25) was centered at 355 nm/ 532 nm. For the sensitization with the Palladium complex, the pump was generated by a home-built noncollinear optical parametric amplifier (NOPA), seeded by a 1 kHz Spectra-Physics Solstice Ace, generating a tuneable pump centred at 650 nm. The signal was recorded on a Hamamatsu silicon detector using a reference beam.

Excitation densities were calculated using ophiropt power density calculator.[10]

Analysis Methods

Spectral deconvolution was achieved using either a genetic algorithm (GA), as described previously, or through singular value decomposition (SVD), combined with a global fit to a series of ordinary differential equations to transform the resulting components into the correct time/wavelength eigenbasis.[9,11] The genetic algorithm was performed in MATLAB, with exponential fits achieved through use of scipy.optimize.curve_fit, and associated errors

obtains from pcov. SVD was performed in python, using the numpy.linalg.svd, and kinetic model fitted with scipy.integrate.odeint and scipy.optimize.minimize (L-BFGS-B) functions. The lifetimes associated with the kinetic ODE model are quoted, with an error associated with the goodness of fit, calculated from both a bootstrapping of the residuals, and from the inverse hessian of the fit.

S7 Steady State Characterisation

Steady state absorption spectra presented in the main text, **Figure 1**, were recorded at room temperature in chlorobenzene solution (0.1 mg ml^{-1}) and thin films drop cast from chlorobenzene stock solutions (OHC 10 mg ml^{-1}, OHAC 2.5 mg ml^{-1}), using the apparatus described in the previous section. PL spectra were obtained during the PLQE measurements. For the thin films in particular, this means that the PL spectra may be obscured by self-absorption of the 0-0 peak due to the presence of an integrating sphere. The absorption and PL spectra were normalised to the 0-0 peak, and the intersection of the two spectra were used to estimate the 0-0 transition energies (SI Table 4 below).

		**PLQE / %**	**Absorbance at 520 nm / OD**	**0-0 transition / nm**	**0-0 transition / eV**
**OHAC**	Solution	74	0.1	624	1.99
	Film	0.5	0.24	620	2.00
**OHC**	Solution	86	0.1	584	2.12
	Film	4.0	0.95	589	2.11

Table S4. Photoluminescence quantum yield, sample absorbance at laser excitation, and 0-0 transitions of aza- cibalackrot OHAC and cibalackrot OHC obtained from PQLE and UV-Vis measurements in thin-film and solution (1mm path length).

S8 Transient Photoluminescence

Excitation Wavelength	Emission Wavelength
510 nm	645 nm

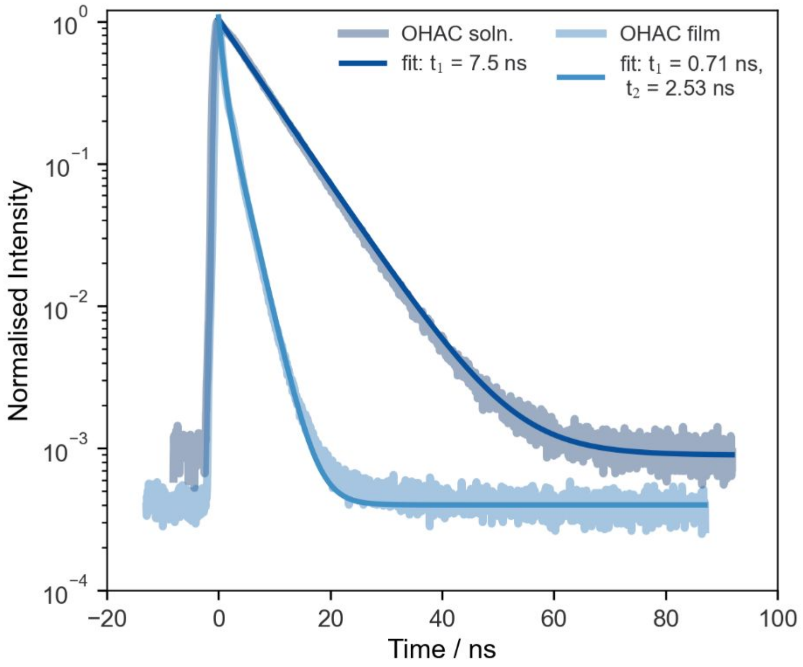

Figure S4 Time correlated single photon counting (TCSPC) traces of OHAC in both solution and thin film, demonstrating a quenching in the film compared to solution, indicating the presence of non-radiative processes in the thin film. (OHAC solution: t_1= 7.5 ± 0.5 ns , OHAC film: t_1= 0.71 ± 0.01 ns (from instrument response), t_2= 2.53 ± 0.03 ns).

S9 Transient Absorption Spectroscopy (Film Excitation Density Dependence)

Transient absorption data of thin films are presented in the main text, **Figure 3**. Raw data are presented, and data normalised to the integral of the spectra. Spectra were presented alongside the spectra of the triplet sensitisation studies, described in the following sections.

Below, in **Figure S5**, we show the excitation fluence dependence of the kinetic profile at the TT region and S_1 region for both OHC (**Figure S5a**) and OHAC (**Figure S5b**). This was achieved by adjusting the laser power of the pump between 200 μW and 825 μW, with a corresponding Gaussian spot size of 646 μm, giving a range of fluences between 3.0e-6 - 1.26e-5 J cm^{-2} . Spot position changed between fluence measurements, resulting in different sample morphologies being probed.

For OHC, the singlet PIA region is 820-840 nm (**Figure S5a**), and triplet PIA region is 710-730 nm (**Figure S5c**), whereas for OHAC the S_1 PIA region is 890-910 nm (**Figure S5b**) and T_1 PIA region is 730-750 nm (**Figure S5d**). For both OHC and OHAC, the singlet PIA grows within the instrument response time, and decays within 1 ns for both. For OHC, the singlet and triplet regions follow the same kinetics, while for OHAC, the singlet and triplet regions have distinct kinetics. For OHAC, the triplet PIA grows in concomitantly with the decay of the singlet PIA. The triplet PIA decay is independent of fluence, suggesting a geminate decay pathway.

Pump Wavelength	Spot size / µm	Power / µW	Repetition Rate	Excitation density / J cm^{-2}
550 nm	646	200 - 1000	40 kHz	3.0e-6 - 1.26e-5

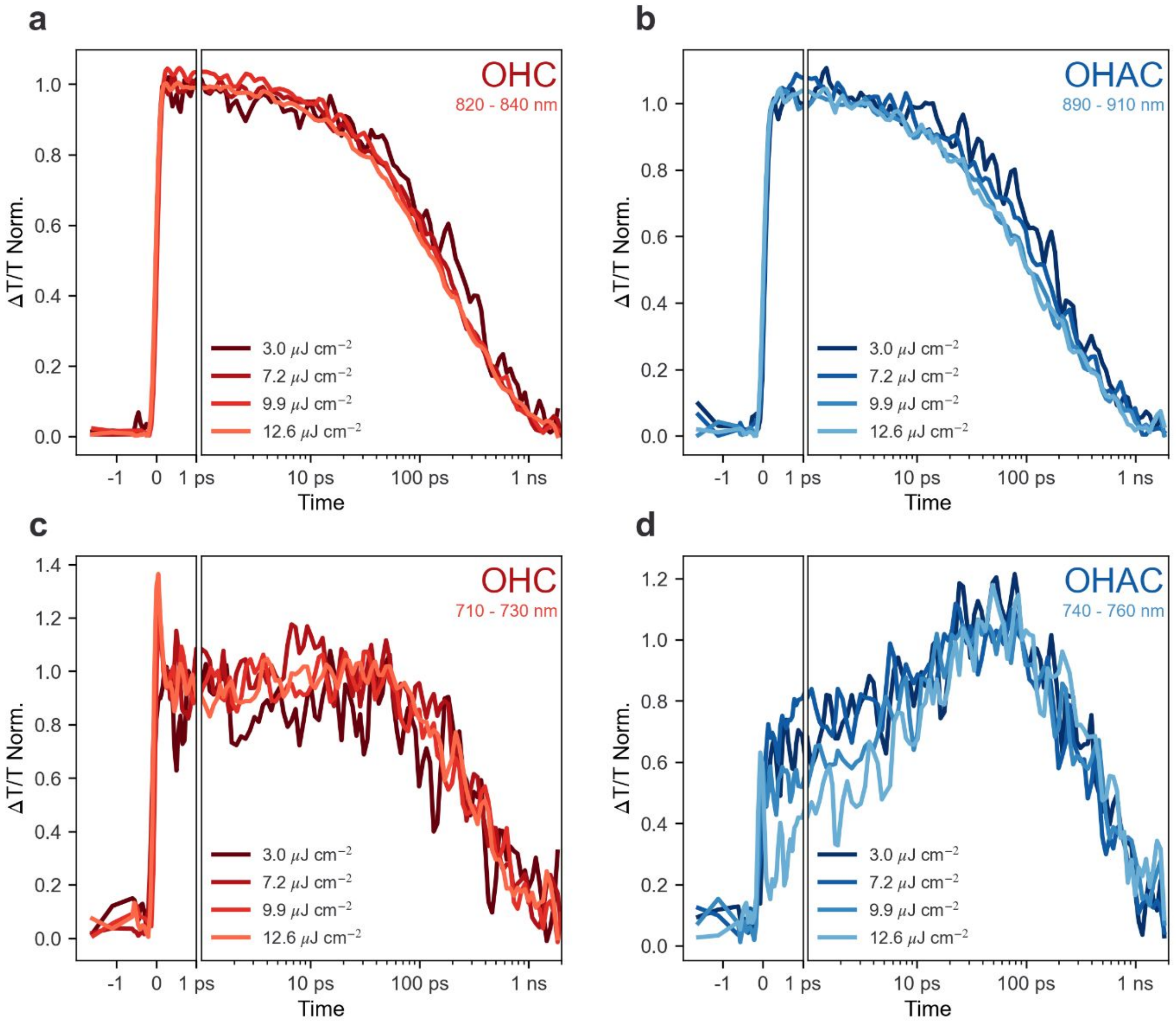

Figure S5 Fluence dependence of psTA on kinetics over wavelength ranges for OHC **(a&c)** and OHAC **(b&d)**. The singlet PIA region (**a**, OHC, **b**, OHAC) and TT state region (**c**, OHC, **d**, OHAC) are shown at increasing excitation power. Absence of change in the decay profile of the TT state in OHAC with fluence indicates the decay pathway is exciton density independent, suggesting geminate TT annihilation. Minimal singlet-singlet annihilation (SSA) is observed in either film at these excitation densities, as seen by little change in the S_1 decay rate.

S10 Transient Absorption Spectroscopy (Solutions)

Pump Wavelength	Spot size	Power	Repetition Rate	Excitation density
550 nm	232 μm	500 μW	40 kHz	5.9e-5 J cm^{-2}

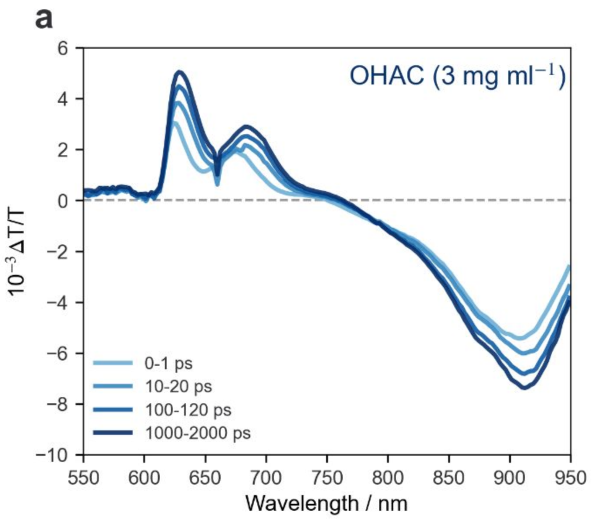

Figure S6: OHAC solution (3 $mgml^{-1}$, 2:1 toluene:THF) transient absorption spectra on picosecond timescale. Rapid (sub 1 ps) formation of excited singlet S1 state, with no other evolution of species. Some spectral shifting at early times. The S_1 begins to decay at 1 ns, which agrees with the ns TA data in **Figure S7**.

Pump Wavelength	Spot size	Power	Repetition Rate	Excitation density
633 nm	295 µm	0.1 mW	1 kHz	2.9e-4 J cm^{-2}

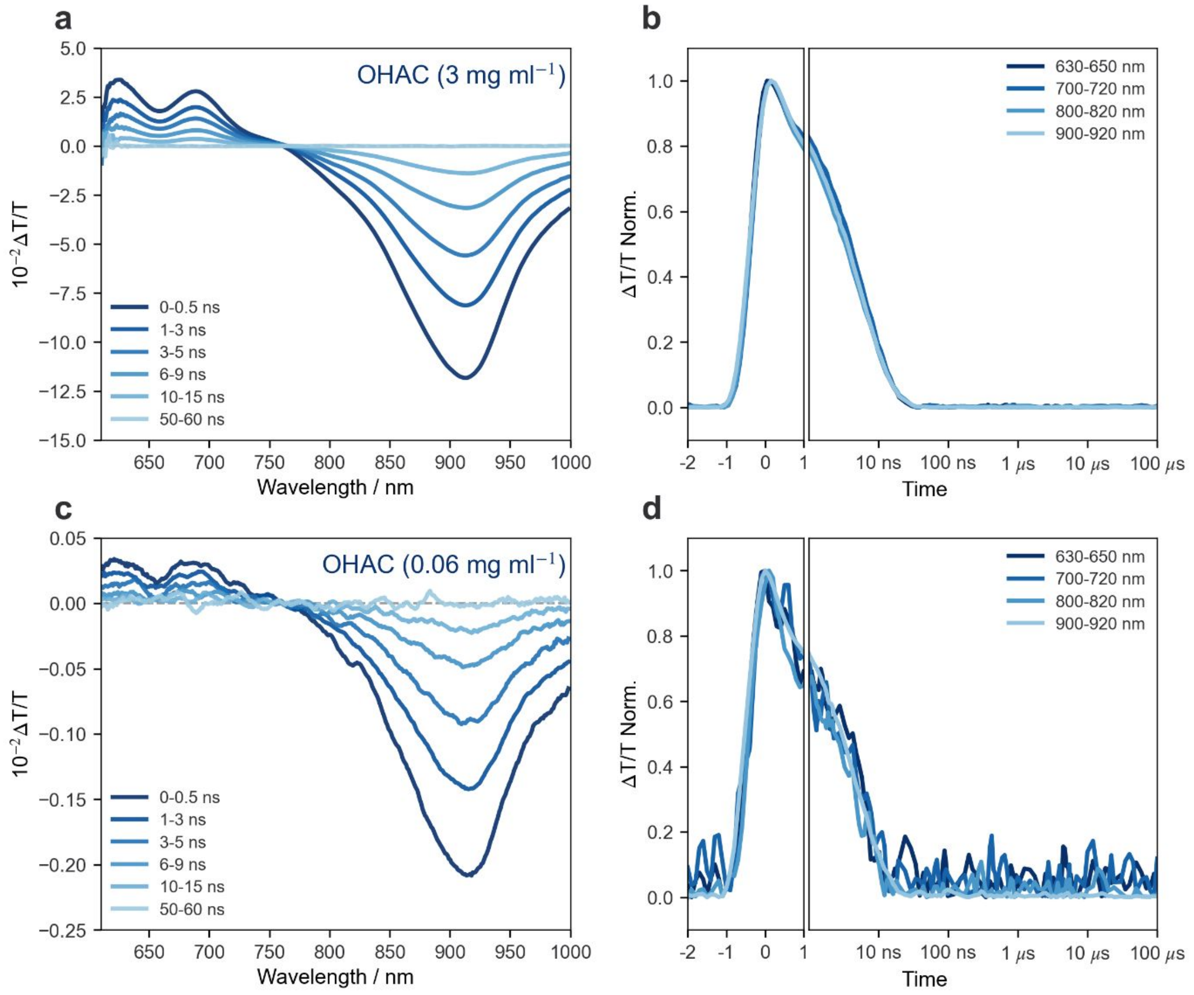

Figure S7: OHAC solution at two concentrations (**a&b**, 3 mgml^{-1}, **c&d** 0.06 mgml^{-1} (50x dilution), 2:1 toluene:THF, 200µm path length) transient absorption spectra on nanosecond timescale. No spectral evolution, with only one state present (S_1). There is rapid decay of S_1 state at both concentrations.

S11 Triplet sensitization – Solution

Anthracene sensitizer

Pump Wavelength	Spot size	Power	Repetition Rate	Excitation density
355 nm	1164 μm	1.0 mW	1 kHz	1.88e-4 J cm^{-2}

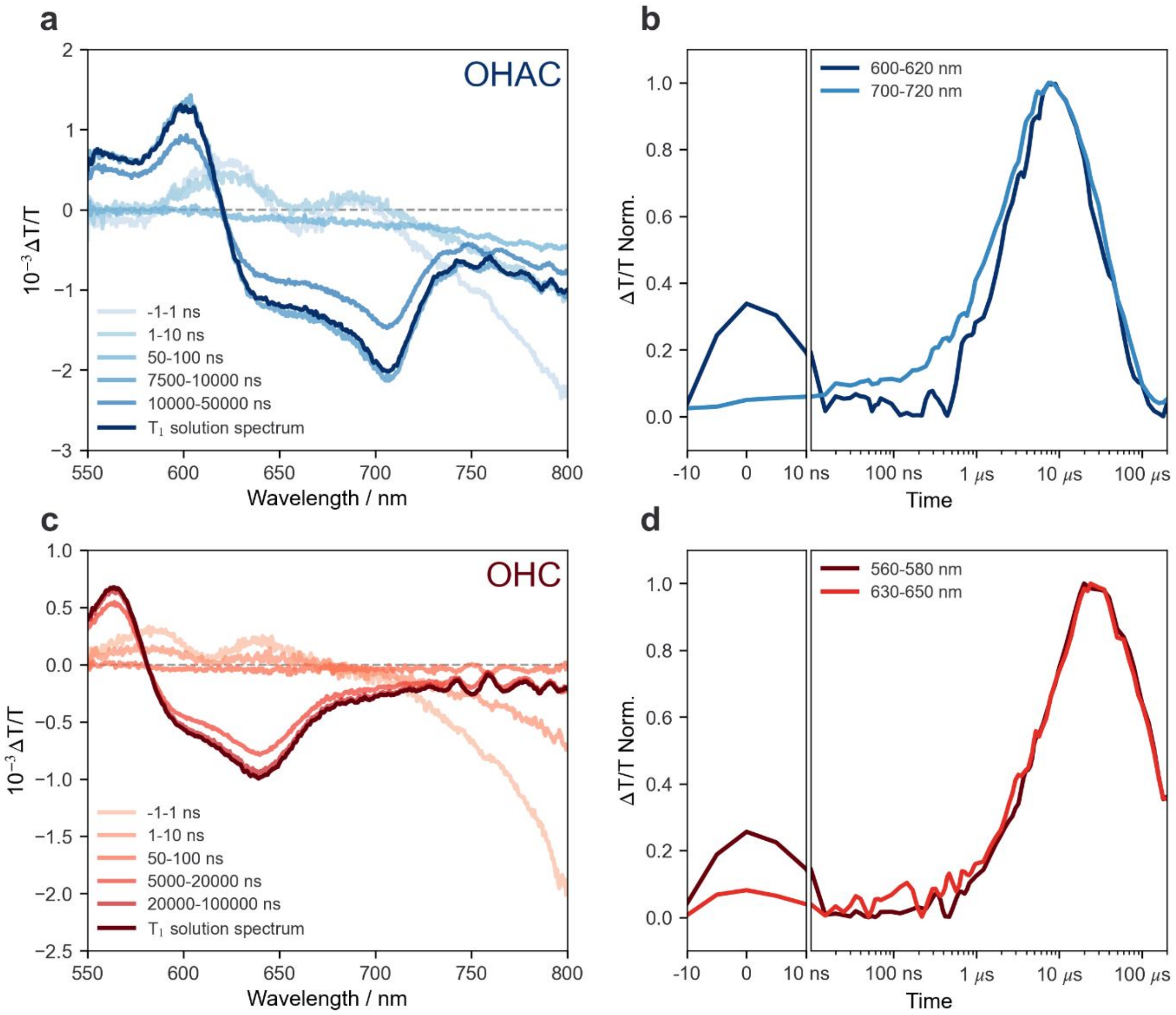

Figure S8 Triplet sensitization measurements in solution of OHAC (**a & b**) and OHC (**c & d**), using anthracene as a sensitiser with 355 nm photoexcitation. Measured with TA on a ns timescale.

Triplet sensitisation measurements were performed in chlorobenzene solution (1mm path length) with anthracene as a sensitiser. Solutions were prepared such that anthracene had an absorbance of 0.2 OD at the excitation wavelength 355 nm, while OHC and OHAC had an absorbance of 0.5 OD at their λ_{max}.

Initial photoexcitation of the anthracene generates free triplets in solution by ISC. Collision between anthracene triplets and cibalackrots in solution results in triplet transfer at late times. Triplet spectra were obtained by averaging the late time signals attributed to the triplets of the sensitised species (signal at ~10^4 ns). Some initial excitation of the cibalackrots occurred, generating the photoexcited singlet S1 spectra (seen in the -1 → 1 ns window spectra), which decayed rapidly (within 10 ns). Thus, we can ignore the contribution of the S_1 state.

There was no evidence of signals from the anthracene molecules in either spectrum as expected due to the probe range being investigated.

PdTPTBP sensitizer

Pump Wavelength	Spot size	Power	Repetition Rate	Excitation density
633 nm	295 µm	0.1 mW	1 kHz	2.9e-4 J cm^{-2}

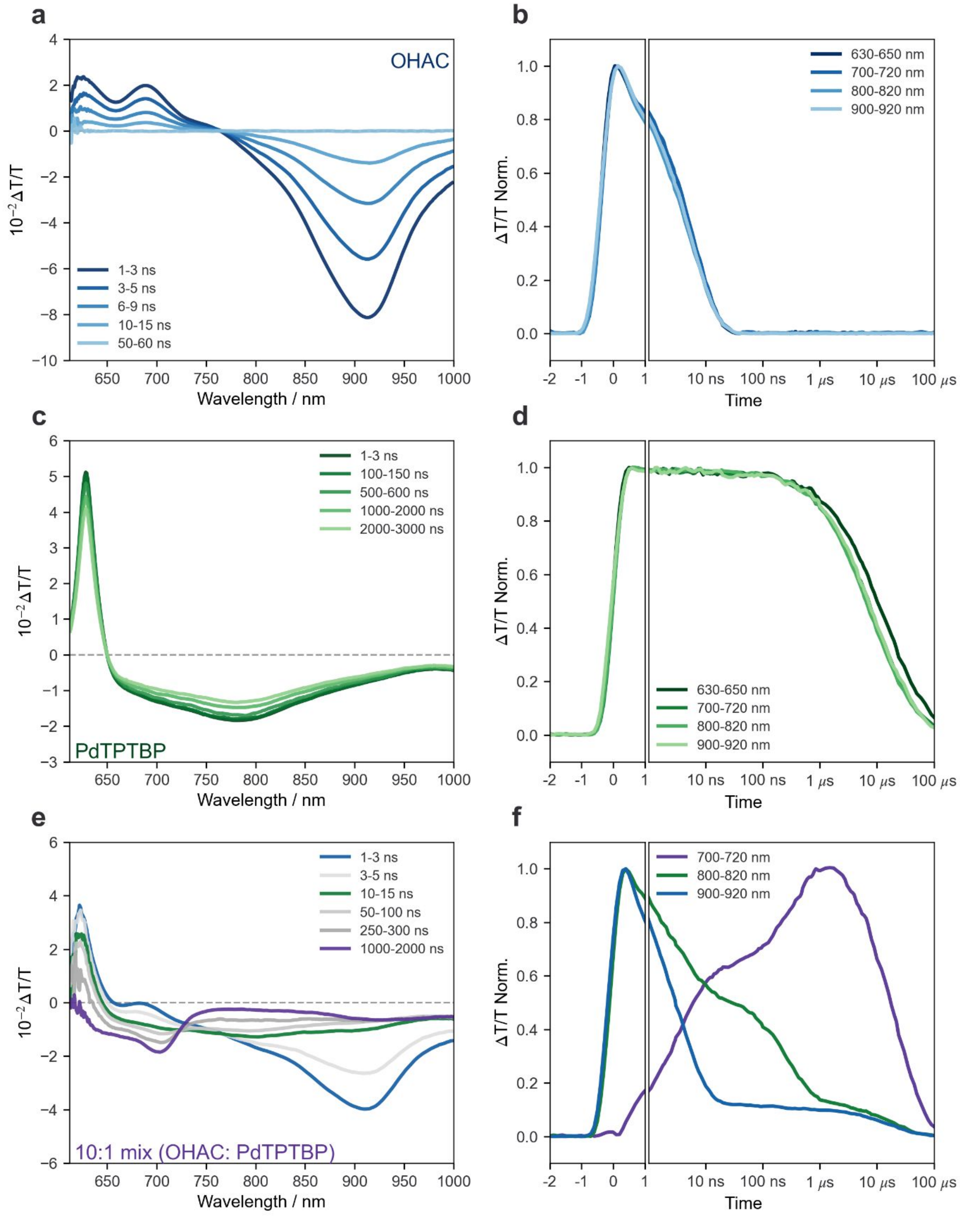

Figure S9 Triplet sensitization measurements in solution (200 µm path length) of OHAC using meso-tetraphenyl-tetrabenzoporphine Palladium Complex (PdTPTBP) as a sensitiser with 632 nm photoexcitation. Measured with TA on a ns timescale.

Repeating the solution state sensitisation with a second sensitiser, meso-Tetraphenyl-tetrabenzoporphine Palladium Complex (PdTPTBP), gave results comparable to those with

anthracene sensitiser. PdTPTBP was purchased from Inochem, Frontier Scientific. Solutions were prepared with 2:1 toluene:THF solvent mix in 200μm path length encapsulated cuvettes, at concentrations of 3 $mgml^{-1}$ (OHAC, 3mM) and 0.25 $mgml^{-1}$ (PdTPTBP, 0.3mM), and mixed to achieve a 10:1 OHAC:PdTPTBP ratio. Excitation and emission spectra of the solutions are given in **Figure S10**.

The transient absorption spectra of OHAC, PdTPTBP, and a 10:1 mix in solution are compared in **Figure S9**. Free triplets on the PdTPTBP form within the first 1 ns and decay over 100 μs (panels **c&d**), which is quenched to less than 1μs by the OHAC organic in the 10:1 mix (panels **e&f**). Again, some initial photoexcitation of the OHAC occurred, highlighted in blue. This contribution again decayed within 10 ns and can be neglected. Free triplets form on the OHAC, as highlighted in purple. The spectrum of the free T_1 state of OHAC agrees well with the spectrum of free triplets from the anthracene sensitisation in **Figure S8**.

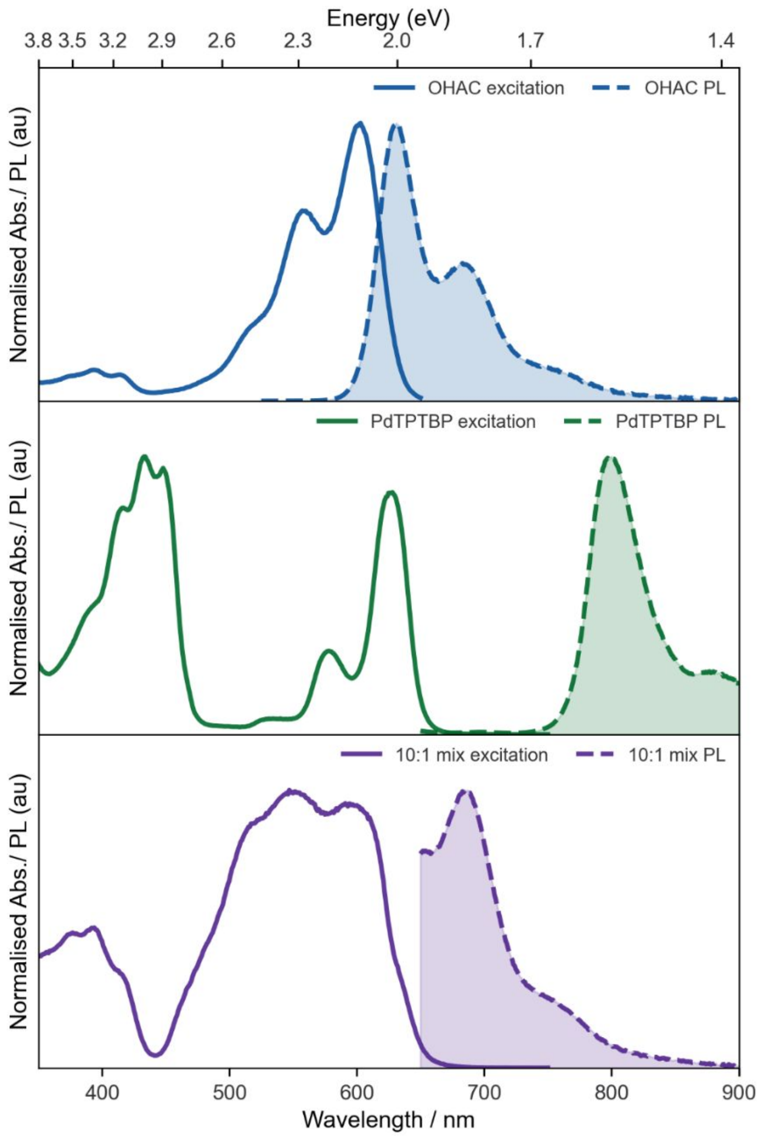

Figure S10 Excitation and emission spectra of OHAC, PdTPTBP, and a 10:1 mix solutions. Emission spectra were recorded with excitation at peak excitation wavelength (510 nm, 630 nm, and 630 nm respectively).

S12 Triplet sensitization – Film (PdTPTBP sensitizer)

Pump Wavelength	Spot size	Power	Repetition Rate	Excitation density
650 nm	325 μm	640 μW	40 kHz	3.86e-5 J cm^{-2}

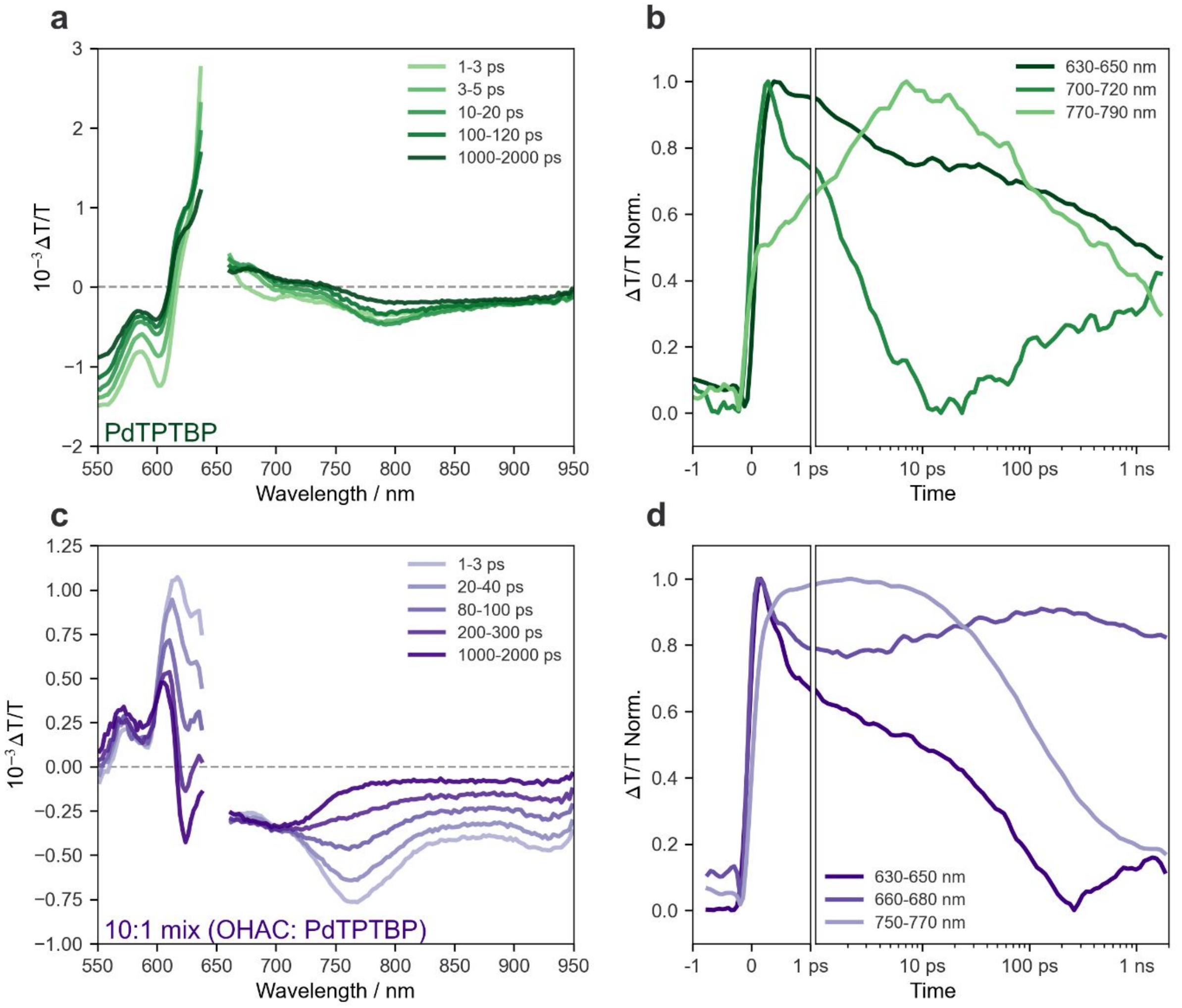

Figure S11 Triplet sensitization measurements in thin film of OHAC using meso-Tetraphenyl-tetrabenzoporphine Palladium Complex (PdTPTBP) as a sensitiser with 650 nm photoexcitation. Measured with TA on a picosecond timescale.

Spectra of PdTPTBP film and a 10:1 mix shown with 650 nm excitation. Pristine OHAC film did not give a signal with 650 nm excitation. Unlike the solution sensitisation (**Figure S9**), there is no contribution from the OHAC S_1 state at early times. Early formation of the PdTPTBP T_1 state is observed in both the pristine sensitiser and 10:1 mix films. The Palladium complex forms free triplets on a sub-ps timescale. In the 10:1 mix, triplet transfer to the OHAC occurs with a lifetime of 130 ps. This is confirmed by spectral deconvolution (**Figures S12-S13**). Excitation and emission spectra of the films are shown in **Figure S14**, with transient PL of the PdTPTBP phosphorescence shown to be quenched in **Figure S15**.

Spectral Deconvolution of 10:1 mix film TA data

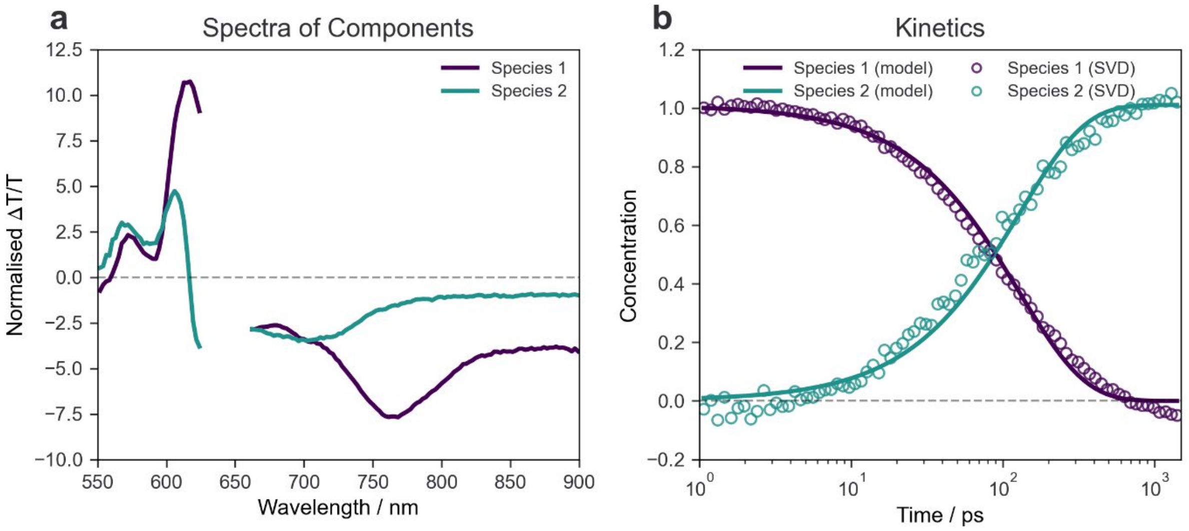

Figure S12 Spectral deconvolution (with SVD and a kinetic model) of 10:1 OHAC:PdTPTBP mix thin film on picosecond timescale. Species 1 is assigned as PdTPTBP T_1 , and Species 2 is assigned as OHAC T_1. The model illustrates how the PdTPTBP T_1 transfers triplet energy to the OHAC with a lifetime of 130 ± 3 ps. Fitted with a simple kinetic model (A → B), with one rate constant. Residuals of the global fit are shown in **Figure S13**.

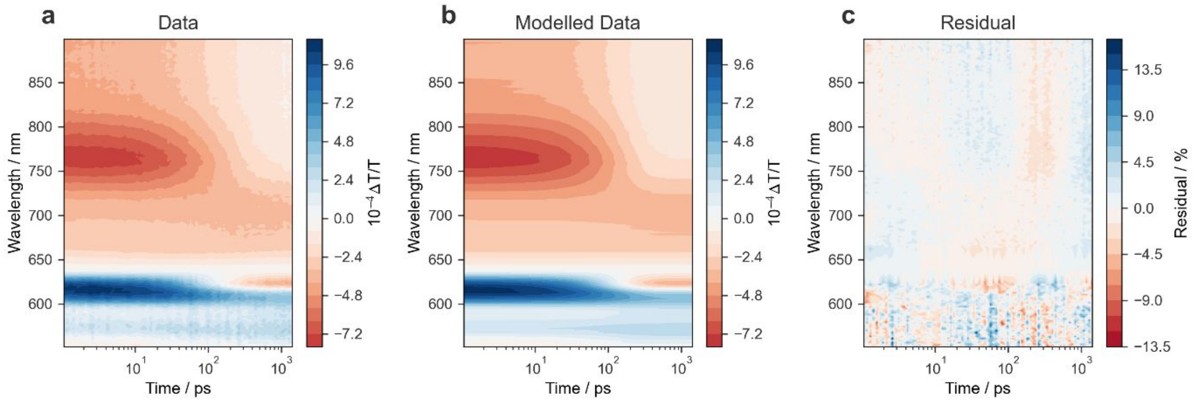

Figure S13 Residuals of the Spectral deconvolution (with SVD and a kinetic model) of 10:1 OHAC:PdTPTBP mix thin film on picosecond timescale. **(a)** shows the raw TA data from **Figure S11 c&d**, **(b)** shows the modelled data presented in **Figure S12**, with panel **(c)** showing the residuals of the fit as a percentage of the absolute maximum ΔT/T of the raw data.

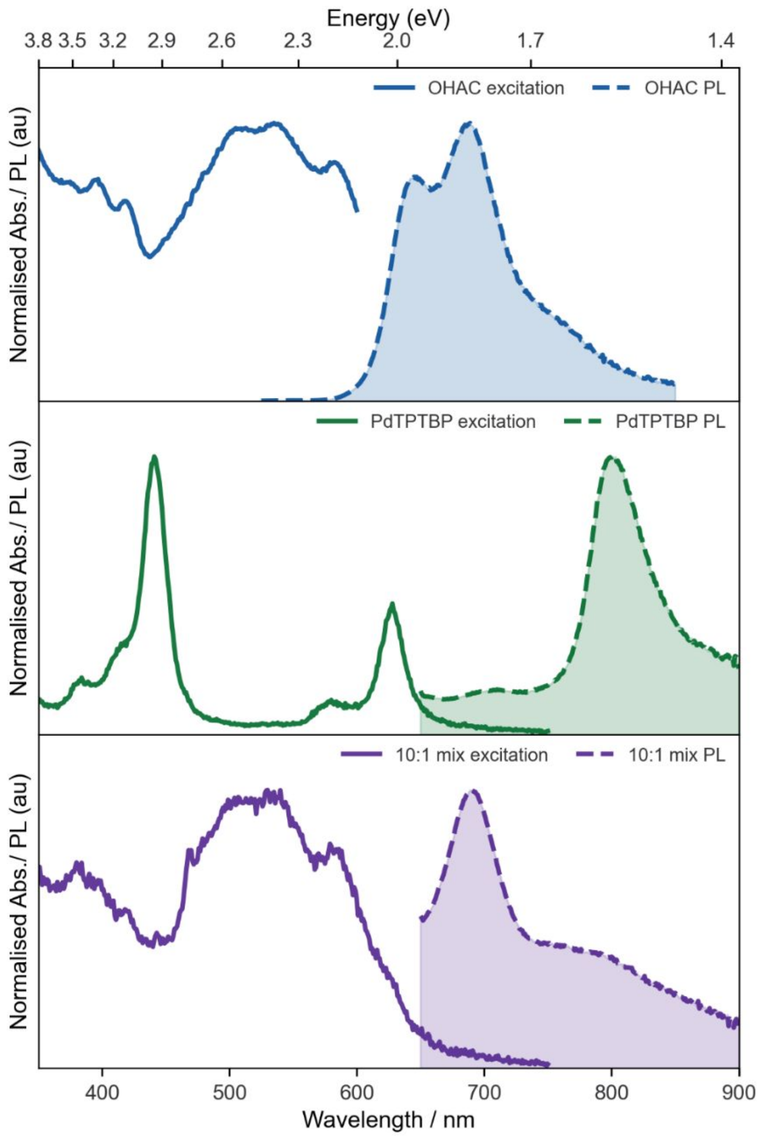

Figure S14 Excitation and emission spectra of OHAC, PdTPTBP, and a 10:1 mix thin films. Emission spectra were recorded with excitation at peak excitation wavelength (OHAC 510nm, PDTPTBP 630nm, 10:1 mix 630nm). The 10:1 mix shows clear evidence of contribution from the PdTPTBP phosphorescence at 800 nm. Excitation spectra were recorded at the following emission wavelengths: OHAC 687nm, PdTPTBP 800nm, 10:1 mix 800 nm.

Excitation Wavelength	Emission Wavelength
630 nm	800 nm

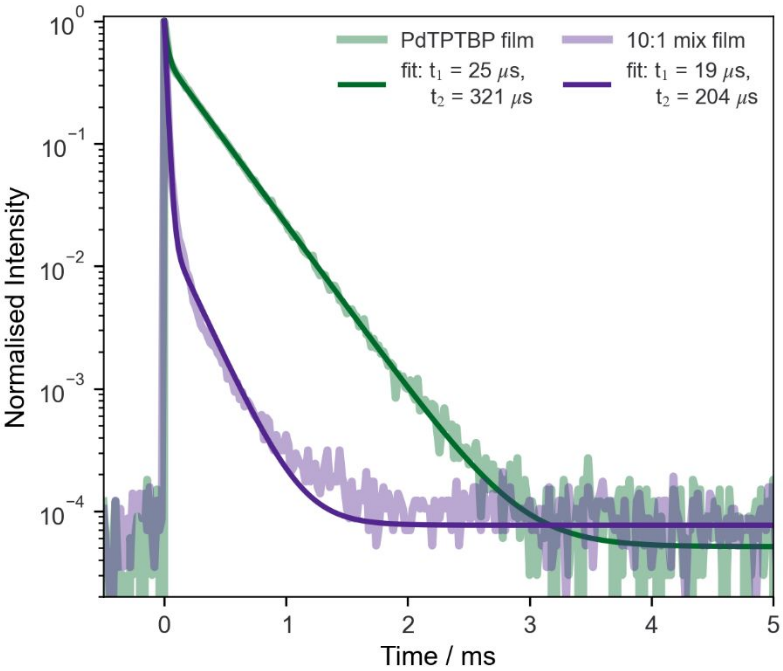

Figure S15 TCSPC of PdTPTBP film and the 10:1 OHAC:PdTPTBP mix film at 800 nm emission, observing the quench in the phosphorescence lifetime of the PdTPTBP T_1 lifetime due to triplet energy transfer to the OHAC. (PdTPTBP film : t_1= 25 ± 0.3 μs, t_2= 321 ± 1.5 μs , 10:1 OHAC:PdTPTBP film : t_1= 19.4 ± 0.1 μs, t_2= 204 ± 17 μs). The long decay of PdTPTBP phosphorescence is quenched form 321 μs to 204 μs, confirming triplet energy transfer is occuring from the PdTPTBP to the OHAC in the mix-film.

S13 Spectral Deconvolution of Transient Absorption Data

Discussion of Genetic Algorithm (GA) Deconvolutions

Here we have used a model-free genetic algorithm to deconvolve the data Comparing the N=2 (Figure S20) to the N=3 models, there appears to be only 2 species, with the third species being a linear combination of the two spectra. This strongly suggests that the model with N = 3 is overparameterized and that the GA deconvolution with N=2 sufficiently captures the spectra and kinetics of the sample. The time constants and spectra are compared to those obtained from a deconvolution which used singular value decomposition, which implements a kinetic model.

N = 2 model

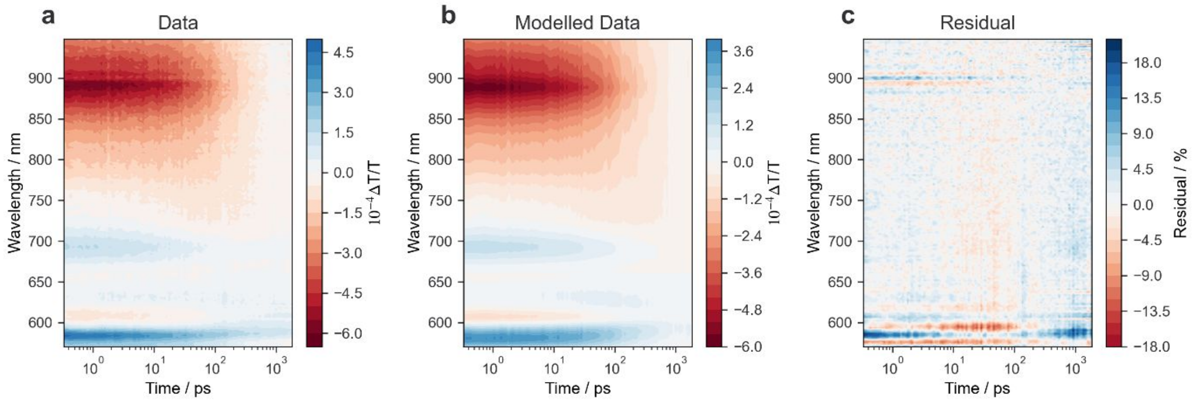

Figure S16 reconstruction of data using a N=2 genetic algorithm. The resulting modelled data are shown in **(b)**, compared to the raw data **(a)** (from main text **Figure 3**), and the residuals **(c)** shown as a percentage of absolute maximum ΔT/T of the raw data.

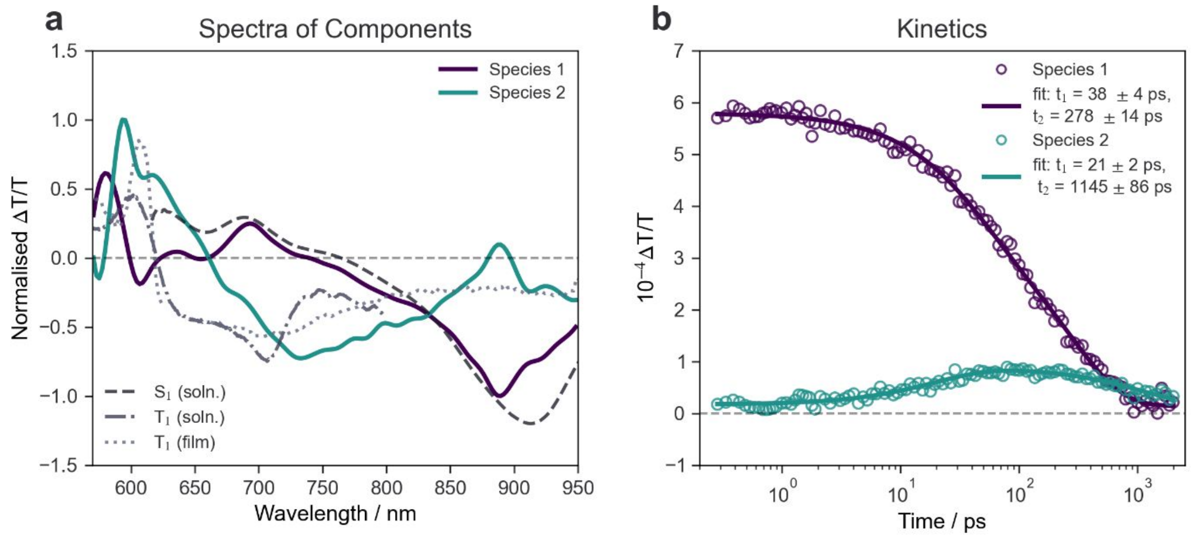

Figure S17 reconstruction of data using a N=2 genetic algorithm, shown in main text **Figure 4**. Spectra in **(a)** are compared the spectra of OHAC solution S_1 (**Figure S7**), solution T_1 (**Figure S8a**), and film T_1 (**Figure S11c**). The kinetics in **(b)** are fitted to independent exponential decays. Species 1 is assigned as the S_1 state and Species 2 the TT state. Species 2 spectrum shows good comparison to the solution T_1 spectrum of OHAC. In the kinetics, Species 1 decay of 38ps matches well with the growth of species 2 of 21ps, so we can say this is concomitant. Species 1 also has a non-radiative decay. The absorption cross section

of Species 2 is lower than Species 1. From the ratios of extinction coefficients in **Table S6**, we can approximate the yield of species 2 to be 29-32%.

N = 3 model

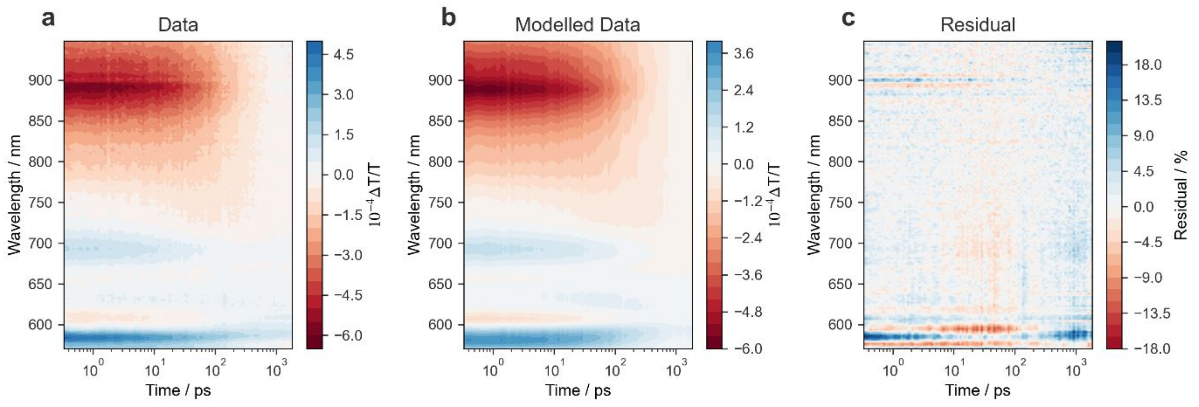

Figure S18 reconstruction of data using a N=3 genetic algorithm. The resulting modelled data are shown in **(b)**, compared to the raw data **(a)** (from main text **Figure 3**), and the residuals **(c)** shown as a percentage of absolute maximum ΔT/T of the raw data. By comparison to **Figure S16c**, the inclusion of a third species does not greatly improve the goodness of fit.

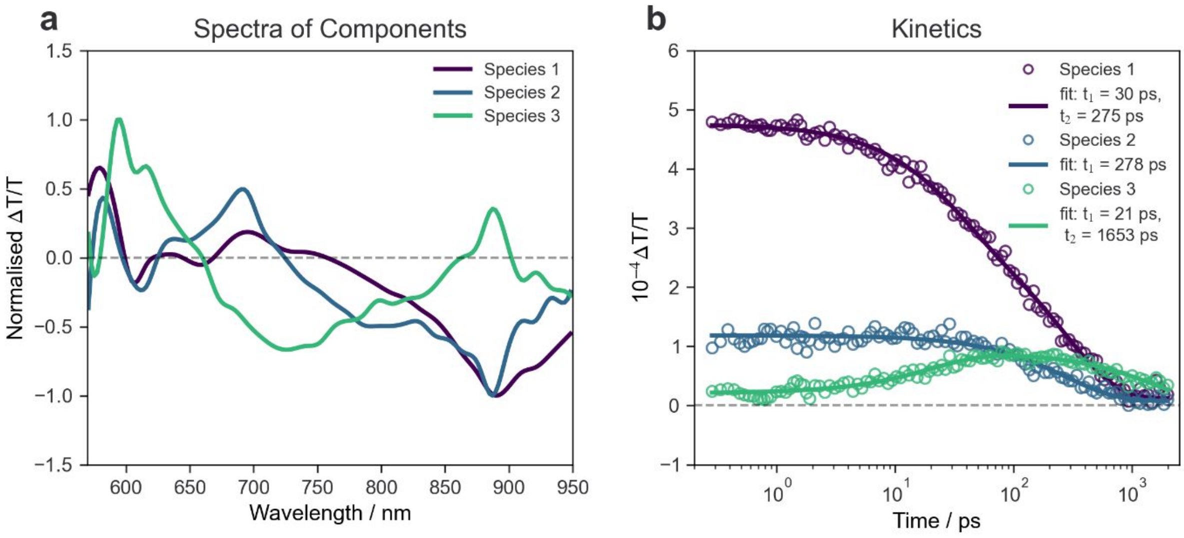

Figure S19 reconstruction of data using a N=3 genetic algorithm. Comparing the N=2 (Figure S17) to the N=3 models, there appears to be only 2 species, with the third species being a linear combination of the two spectra. This strongly suggests that the model with N = 3 is overparameterized and that the GA deconvolution with N=2 sufficiently captures the spectra and kinetics of the sample.

Comparison to Singular Value Decomposition

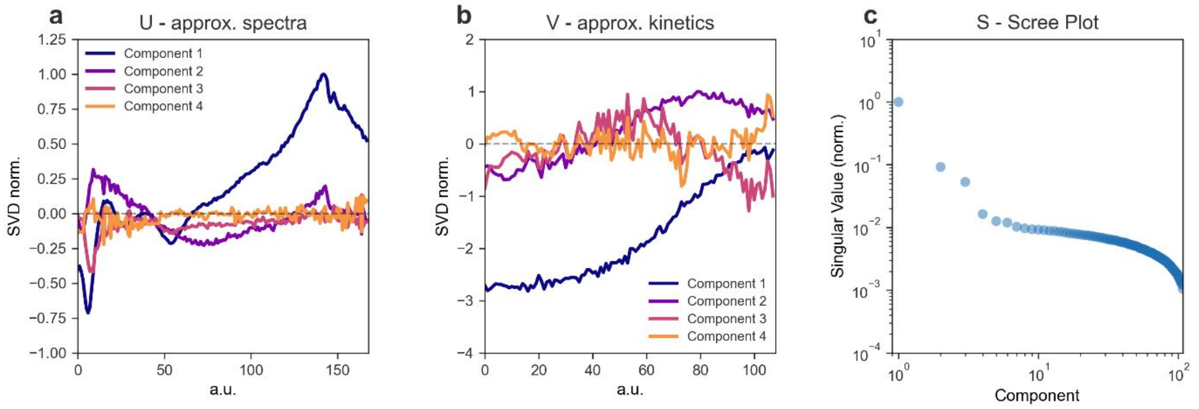

Figure S20 Output from singular value decomposition, showing the first N=4 principal components in their arbitrary unphysical eigenbasis, which approximately correspond to spectra (a) and kinetics (b), with the scree plot, or plot of the singular values, shown in (c). The lack of structure in the 4th component, and the results of the scree plot, illustrate that the dataset can be well described with 2 or 3 principal components.

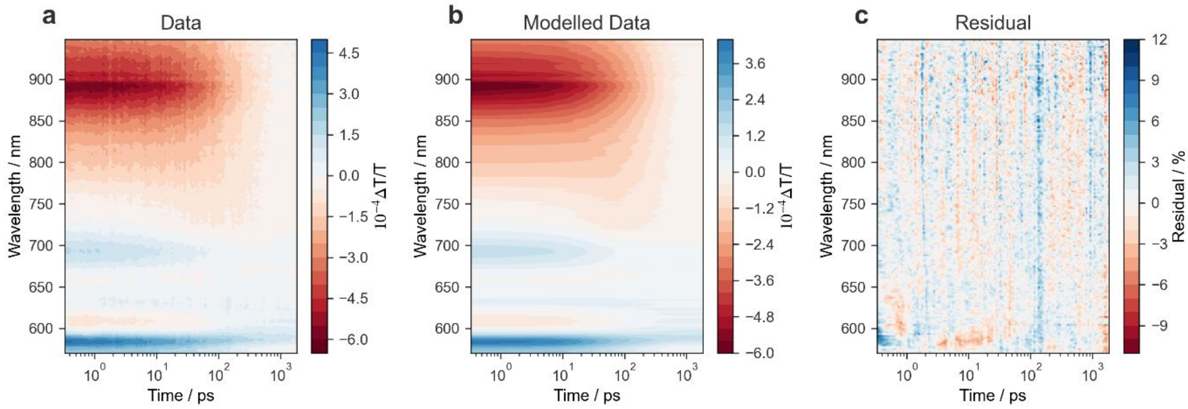

Figure S21 Using the first N=3 principal components from singular value decomposition (SVD), the SVD 'spectra' and 'kinetics' are rotated into the correct wavelength/time eigenbasis by fitting a kinetic model to the 'kinetics'. The resulting modelled data are shown in **(b)**, compared to the raw data **(a)** (from main text **Figure 3**), and the residuals **(c)** shown as a percentage of absolute maximum ΔT/T of the raw data.

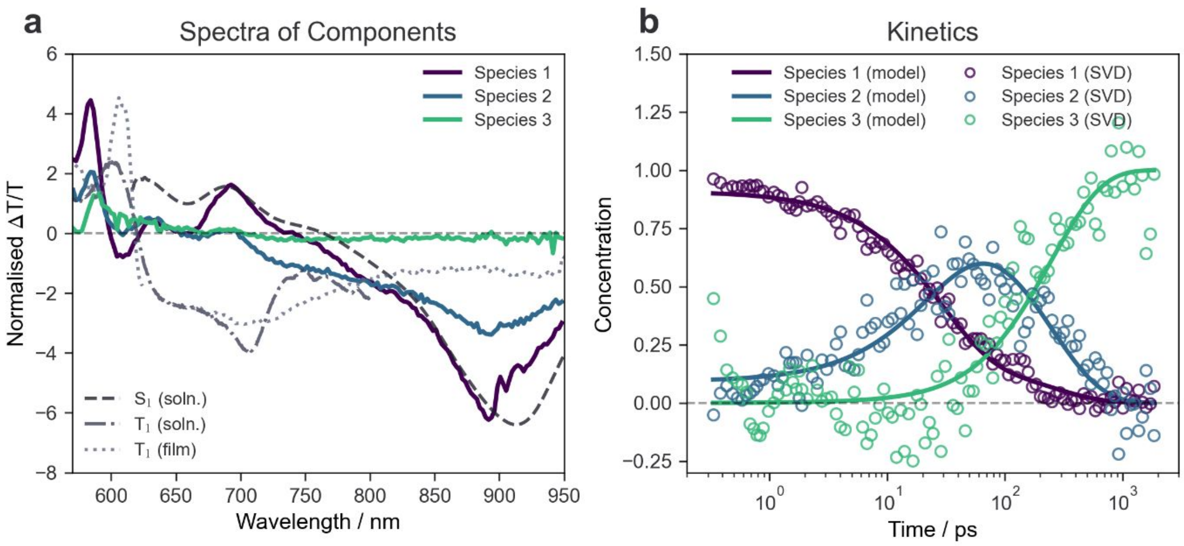

Figure S22 Spectra and associated kinetics from the SVD shown in SI **Figure S21b**, fitted to the kinetic model shown below. Species 1 is assigned as the S_1 state, Species 2 is assigned as the TT state, and Species 3 is assigned as the late time noise. Species 3 matches well with the late time (1ns) signals shown in **Figure S21a**. TT forms reversibly from the S_1 with a forwards lifetime of 36 ± 1 ps and reverse of 188 ± 10 ps. S_1 also decays with a second lifetime of 574 ± 40 ps, which we assign as non-radiative decay. The TT state decays with a lifetime of 220 ± 2 ps. Spectra are compared to the spectra of OHAC solution S_1 (**Figure S7**), solution T_1 (**Figure S8a**), and film T_1 (**Figure S11c**). These spectral comparisons confirm a close match between Species 1 and the solution S_1 spectrum. Species 2 shows some characteristic of species 1, but with a distinct additional, broad PIA at 740 which is the region associated with triplet PIAs.

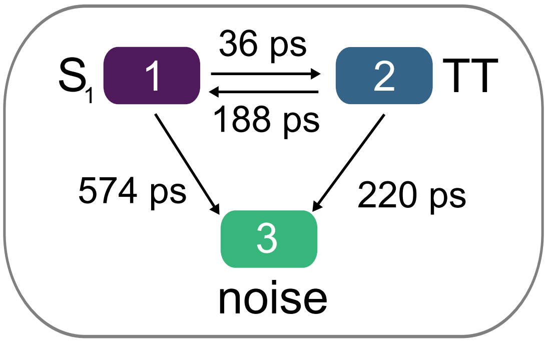

Comparison between the results of genetic algorithm and SVD deconvolutions show many similarities. Both illustrate the data can be well described with 2 significant species. Species 1 decays into species 2 with a lifetime of 21-38 ps. Species 1 has a second decay on the order of 200-500 ps which we attribute to non-radiative decay. Species 1 in both GA and SVD matches well with the OHAC S_1 spectrum. Species 2 in both shows an increased PIA between 700-750 nm, which overlaps well with the OHAC T_1 spectra. This is more significantly demonstrated with the GA analysis compared to the SVD. The SVD struggles to fully deconvolve the species 1 and species 2 spectra, as species 2 shows some characteristics of species 1. However, overall this has limited impact on the kinetics and yield of species 2. The SVD model shows some evidence that there may be an equilibrium between species 1 and species 2. However, this is not confirmed in the GA analysis. Further investigation would be required to fully elucidate the mechanism of singlet fission in this system.

S13 Spectral shifts of T_1 state (INDTs – from literature)

Molecule	Peak of T_1 PIA from sensitization / nm	Peak of T_1 PIA from SF in films / nm	Apparent Red-Shift / nm
Cl-INDT	650	680	30
F-INDT	635	635	0
H-INDT	635	n/a	n/a
CN/Br-INDT	680	720	50
Br-INDT	655	695	40
CN-INDT	700	740	40

Table S5 Page S35 - S36 of Supplementary Information from Fallon *et al.*[3] Indolonaphthyridine thiophenes (INDTs) are similar systems to the cibalackrots, and were confirmed to have high yields of singlet fission. Most INDT systems investigated exhibited a strong red-shifting of the triplet photoinduced absorption (PIA) in the pristine films, when compared to the free triplets generated by sensitisation experiments.

S14 Species 2 (TT) yield estimation

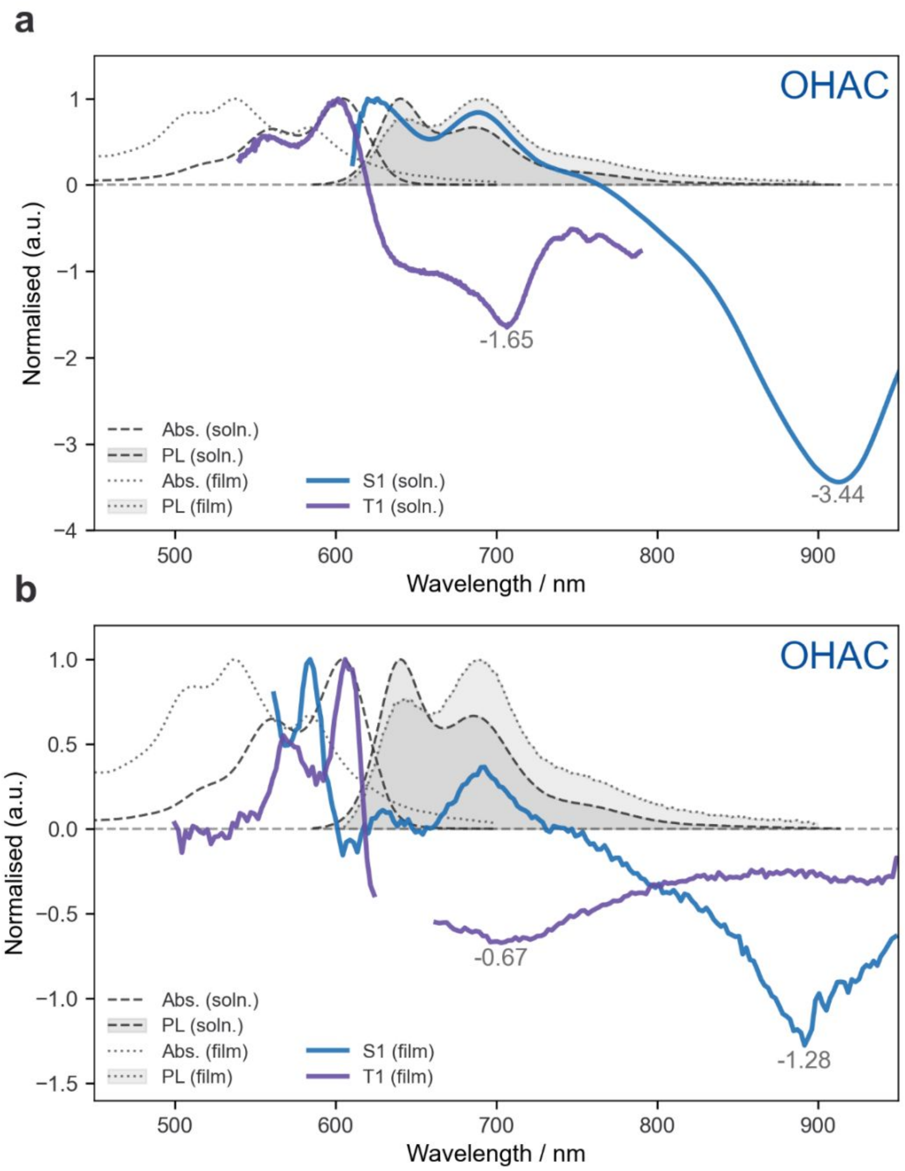

Figure S23 Estimation of the ratio of extinction coefficient of S_1: T_1 photoinduced absorption (PIA), summarized in **Table S6**. The spectra of the S_1 state from pristine film/solution TA measurements are compared to the spectra of free triplets, T_1, generated from the previously described sensitization experiments. Normalized steady state absorbance (abs.) and photoluminescence (PL) spectra are added for reference.

	S_1	T_1	Ratio T_1/S_1
Solution	-3.44	-1.65	0.48
Film	-1.28	-0.67	0.52

Table S6 Estimated ratio of the extinction coefficients of OHAC S_1: T_1 photoinduced absorption (PIA) in both solution and film. Both in thin-film and solution, it is indicated that the T_1 photoinduced absorption has approximately half the extinction coefficient of the S_1 PIA.

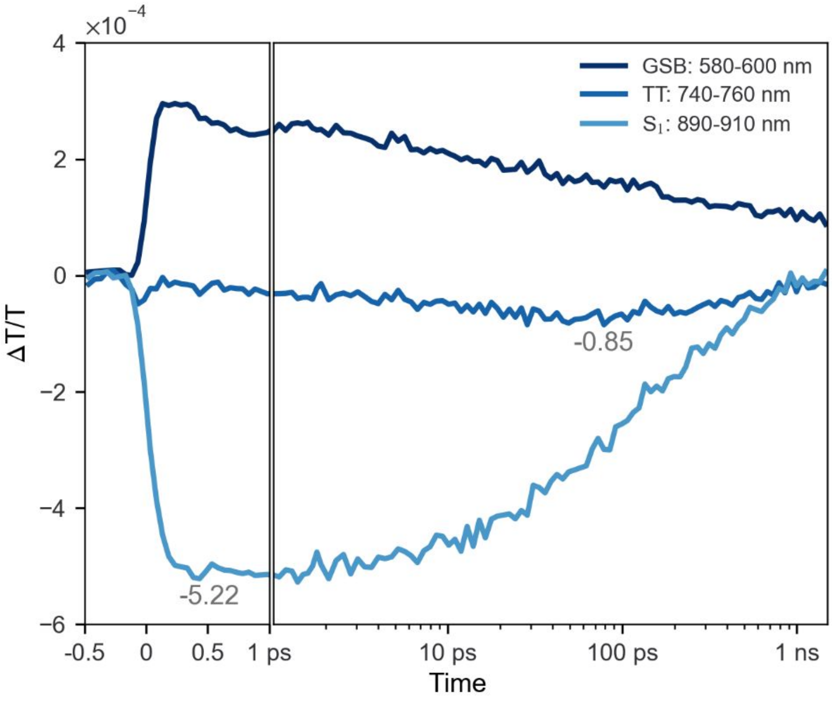

Figure S24 Estimation of TT yield from the ratio of PIA maxima of the S_1 and TT regions in the transient absorption spectra, summarized in Table S6.

**Max S1**	**Max TT**	**Max TT / Ratio (solution)**	**Yield**	**TT/ Ratio (film)**	**Yield**
-5.22	-0.85	-1.77	0.34	-1.63	0.31

Table S7 Estimating the TT yield, using the ratio of the maximal intensities of the S_1 and TT regions, combined with the ratio of extinction coefficients of the species from **Table S6**.

Comparison to results from N=2 genetic algorithm deconvolution

**Max S1**	**Max TT**	**Max TT / Ratio (solution)**	**Yield**	**TT/ Ratio (film)**	**Yield**
5.94	0.90	1.875	0.32	1.731	0.29

Table S8 Estimating the TT yield, using the ratio of the maximal intensities of the S_1 and TT regions from genetic algorithm model (N=2) from **Figure S17b**, combined with the ratio of extinction coefficients of the species from **Table S6.**

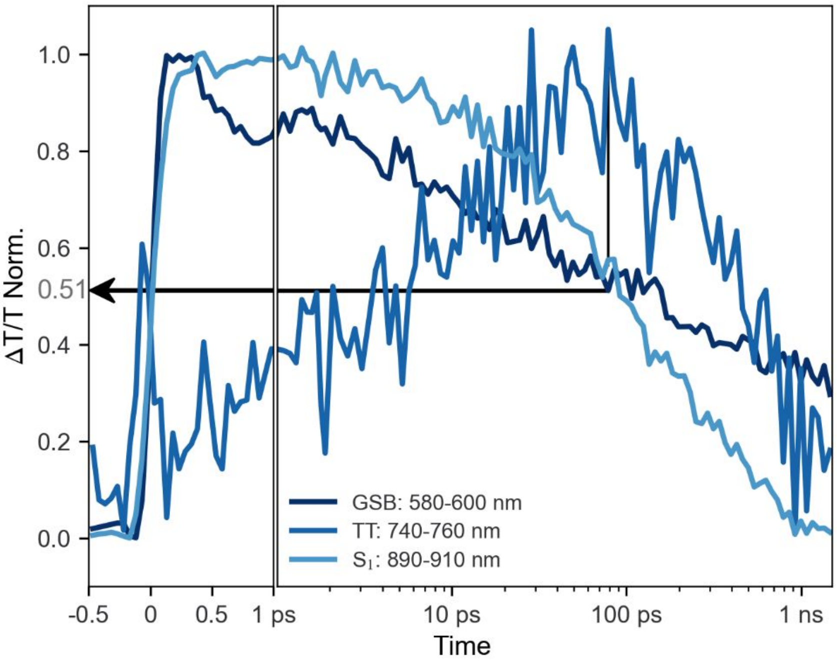

Figure S25 Estimation of TT yield from decay of GSB, which is an over approximation, estimating the percentage population of all remaining excited states at the peak of formation of the TT state.

Method	Approximate TT Yield (%)
Ratio solution (Table S7)	34
Ratio film (Table S7)	31
Decay of GSB population (Figure S25)	51
Genetic Algorithm (Table S8)	29-32
SVD (Figure S22)	60

Table S9 Summary of approximate TT yields from the various methods discussed.

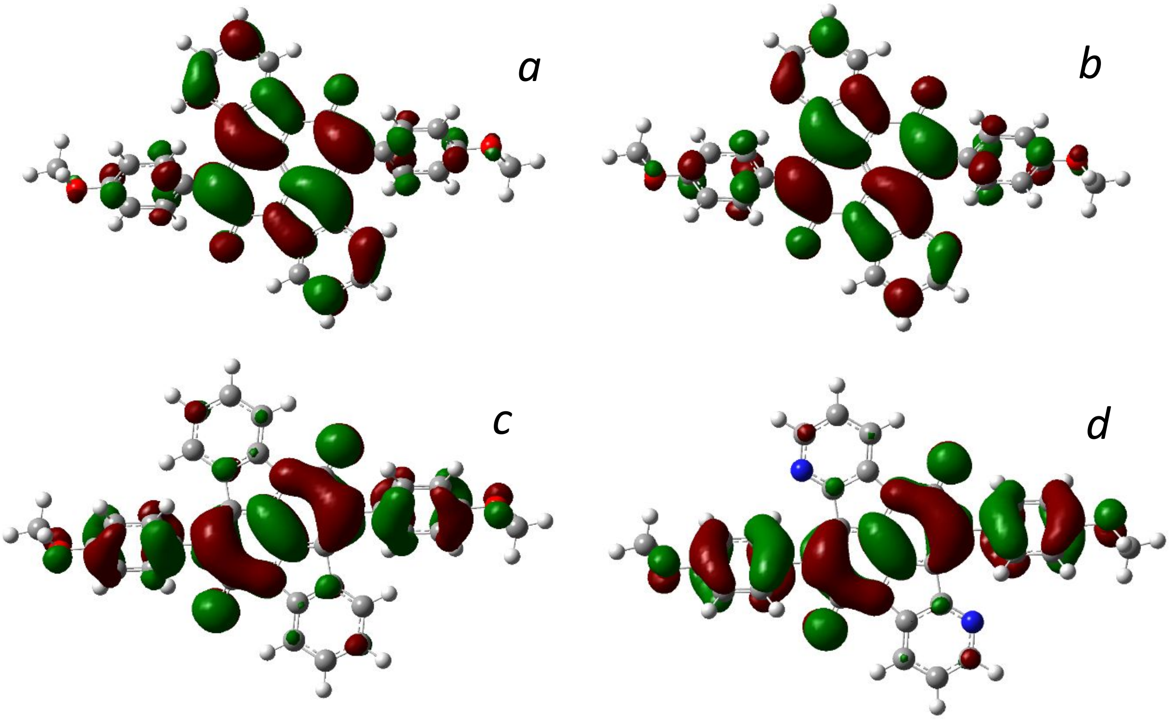

Figure S26 Frontier molecular orbital distribution (performed at B3LYP/G** level) of OHC (left) and OHAC (right). (a) OHC LUMO, (b) OHAC LUMO, (c) OHC HOMO, (d) OHAC HOMO.

References

1. Kolaczkowski, M.; He, B. A.; & Liu, Y. Stepwise Bay Annulation of Indigo for the Synthesis of Desymmetrized Electron Acceptors and Donor-Acceptor Constructs. *Org. Lett.* **2016**, *18*, 5224–5227.
2. CrysAllisPro, Agilent Technologies Inc., 2014.
3. Sheldrick, G. M. SHELXT - Integrated space-group and crystal-structure determination. *Acta Crystallogr A*., **2015**, *64*, 3–8.
4. Dolomanov, O. V.; Bourhis, L. J.; Gildea, R. J.; Howard, J. A. K.; Puschmann, H. OLEX2: a complete structure solution, refinement, and analysis program., *J. Appl. Crystallogr*. **2009**, *42*, 339–341.
5. Sheldrick, G. M. Crystal structure refinement with SHELXL. *Acta Crystallogr C*., **2015**, *71*, 3–8.
6. Hübschle, C. B.; Sheldrick G. M.; Dittrich, B. ShelXle: a Qt graphical user interface for SHELXL, *J. Appl. Cryst*., **2011**, *44*, 1281–1284.
7. De Mello, J. C.; Wittmann, H. F.; & Friend, R. H.; An improved experimental determination of external photoluminescence quantum efficiency. *Adv. Mater.* **1997**, *9*, 230–232.
8. Fallon, K. J.; Budden, P.; Salvadori, E.; Ganose, A. M.; Savory, C. N.; Eyre, L.; Dowland, S.; Ai, Q.; Goodlett, S.; Risko, C.; Scanlon, D. O.; Kay, C. W. M.; Rao, A.; Friend, R. H.; Musser, A. J.; Bronstein, H. Exploiting Excited-State Aromaticity to Design Highly Stable Singlet Fission Materials. *J. Am. Chem. Soc.* **2019**, *141* (35), 13867–13876.
9. Rao, A.; Chow, P. C. Y.; Gélinas, S.; Schlenker, C. W.; Li, C. Z.; Yip, H. L.; Jen, A. K. Y.; Ginger, D. S.; Friend, R. H. The Role of Spin in the Kinetic Control of Recombination in Organic Photovoltaics. *Nature* **2013**, *500* (7463), 435–439.
10. *Ophiropt Laser Power Density Calculator*. https://www.ophiropt.com/laser--measurement/power-density-calculator (accessed 2022-04-05).
11. Gélinas, S.; Paré-Labrosse, O.; Brosseau, C. N.; Albert-Seifried, S.; McNeill, C. R.; Kirov, K. R.; Howard, I. A.; Leonelli, R.; Friend, R. H.; Silva, C. The Binding Energy of Charge-Transfer Excitons Localized at Polymeric Semiconductor Heterojunctions. *J. Phys. Chem. C* **2011**, *115* (14), 7114–7119.
